# Stimulation of Local Cytosolic Calcium Release by Photothermal Heating for Studying Intra‐ and Intercellular Calcium Waves

**DOI:** 10.1002/adma.202008261

**Published:** 2021-05-05

**Authors:** Dingcheng Zhu, Lili Feng, Neus Feliu, Andreas H. Guse, Wolfgang J. Parak

**Affiliations:** ^1^ Fachbereich Physik CHyN Universität Hamburg Luruper Chaussee 149 22761 Hamburg Germany; ^2^ College of Material, Chemistry and Chemical Engineering Hangzhou Normal University Yuhangtang road 2318 Hangzhou 311121 China; ^3^ CAN Fraunhofer Institut Grindelallee 117 20146 Hamburg Germany; ^4^ Department of Biochemistry and Molecular Cell Biology University Medical Center Hamburg‐Eppendorf Martinistraße 52 20246 Hamburg Germany; ^5^ National Engineering Center for Nanotechnology (NECN) Shanghai Jiao Tong University Dongchuan road 800 Shanghai 200240 China

**Keywords:** gold nanoparticles, intracellular calcium, lysosomal calcium release, photothermal heating

## Abstract

A methodology is described that allows for localized Ca^2+^ release by photoexcitation. For this, cells are loaded with polymer capsules with integrated plasmonic nanoparticles, which reside in endo‐lysosomes. The micrometer‐sized capsules can be individually excited by near‐infrared light from a light pointer, causing photothermal heating, upon which there is a rise in the free cytosolic Ca^2+^ concentration ([Ca^2+^]_i_). The [Ca^2+^]_i_ can be analyzed with a Ca^2+^ indicator fluorophore. In this way, it is possible to excite local lysosomal Ca^2+^ release in a desired target cell.

## Graphical abstract

A methodology is reported to trigger intra‐ and intercellular calcium waves by photoexcitation. Polymeric capsules with integrated plasmonic nanoparticles inside lysosomes are individually excited by near‐infrared light to transiently release small amounts of Ca^2+^, which can evoke abundant Ca^2+^ release from the endoplasmic reticulum, leading to further propagating intra‐ and intercellular calcium waves.

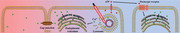

## 1 Introduction

Ca^2+^ signaling is an important pathway of intra‐ and intercellular communication, which directly controls several biological processes including metabolism, secretion, fertilization, proliferation, smooth muscle contraction,^[^ ^1^ ^]^ neuron signal transmission,^[^ ^2^ ^]^ and differentiation of stem cells.^[^ ^3^ ^]^ Dysregulation of Ca^2+^ signaling contributes to Alzheimer's disease,^[^ ^4^ ^]^ bipolar disorder, cardiac disease, schizophrenia,^[^ ^5^ ^]^ and cancer.^[^ ^6^ ^]^ Due to its importance, several experimental techniques have been developed to study Ca^2+^ signaling. To observe local cytosolic Ca^2+^ concentrations, typically Ca^2+^ indicator fluorophores are used, which allow laterally resolved interrogation of local Ca^2+^ levels based on fluorescence microscopy.^[^ ^7^ ^]^ Excitation of Ca^2+^ release into the cytosol can be triggered by electrical, chemical, mechanical, or optical stimuli. Controlled electrical and chemical stimulation, in general, requires the presence of a local device, such as an electrode to apply an electric potential,^[^ ^8^ ^]^ or a microperfusion system to apply neurotransmitters, etc.,^[^ ^9^ ^]^ and thus it is technically challenging to apply simultaneously multiple stimuli, in particular inside the tissue. Similar arguments are true for mechanical stimulation.^[^ ^10^ ^]^ On the other hand, optical stimulation can be for example based on caged‐Ca^2+^ compounds, where upon a local light trigger Ca^2+^ is released from its “cage” into the cytosol.^[^ ^11^ ^]^ As light triggers can be applied with a microscope with an integrated light pointer with a focus of a few micrometers, the site of stimulation can be easily selected by moving the light pointer, and by scanning and modulating the light pointer Ca^2+^ can be released at different sites quasi‐simultaneously. In principle, there is also the option of making the stimulation at several cells simultaneously by using a defocused laser.^[^ ^12^ ^]^ Caged Ca^2+^ compounds however have some limitations. The released Ca^2+^ will be recomplexed by unloaded and unphotolyzed cages. Chemical remnants of the cage may interfere with the endogenous Ca^2+^ toolkit, for example, by activation or inhibition of Ca^2+^ channels or Ca^2+^ pumps. Mg^2+^ can compete with Ca^2+^ to bind to some types of compounds, which also contributes to a slow increase in free cytosolic Ca^2+^ concentration ([Ca^2+^]_i_). Those compounds may be uncaged in the near‐ultraviolet range, while uncaging is quite inefficient by visible light. The photolysis upon illumination is also irreversible.^[^ ^11^, ^13^ ^]^

Recently it has been shown that locally applied heat pulses by photoinduced temperature changes of nanoparticles (NPs) close to cells can trigger Ca^2+^ release.^[^ ^14^ ^]^ It has also been discussed that [Ca^2+^]_i_ can be increased from intracellular Ca^2+^ stores through the d‐*myo*‐inositol 1,4,5‐triphosphate (IP_3_) signaling pathway.^[^ ^15^ ^]^ While this also involves light‐excitation as a trigger, there is a fundamental difference compared to caged Ca^2+^. For caged Ca^2+^ compounds there is release from complexed Ca^2+^ (i.e., the caged Ca^2+^ compounds) which has to be pre‐loaded into cells and cannot be triggered for a second time after Ca^2+^ release. For the plasmonic NPs, Ca^2+^ release happens from intracellular Ca^2+^ stores, which means that upon repetitive light triggering subsequent Ca^2+^ release can be initiated. It is important not to confuse this with photothermal therapy, by which light‐triggered heating of plasmonic NPs is used to initiate cell apoptosis.^[^ ^16^ ^]^ For the light‐triggered release of stored Ca^2+^, the created heat pulse must not affect cell viability, but only initiate Ca^2+^ release, which is possible by choosing the right parameters.^[^ ^17^ ^]^ For the release of stored Ca^2+^ upon photothermal heating of NPs, cells first have to be pre‐loaded with plasmonic NPs. For gold NPs this is possible over weeks without interfering with cell viability.^[^ ^18^ ^]^ Plasmonic NPs are endocytosed and then located in vesicular compartments (i.e., endosomes, lysosomes) around the cell nucleus.^[^ ^19^ ^]^ Due to their small size and intracellular distribution, the plasmonic NPs are however complicated to be directly visualized with optical microscopy, in particular for cells inside 3D cell cultures such as spheroids. However, many plasmonic NPs can be embedded inside a matrix, such as in polymer capsules.^[^ ^20^ ^]^ These capsules can be made of non‐degradable polymers, which retain their integrity after endocytosis. In contrast to the same amount of free endocytosed NPs, NPs endocytosed within a carrier capsule remain located within the micrometer‐sized capsule. Thus, despite the same amount of intracellular plasmonic NPs, there is a higher local concentration of plasmonic NPs (at the site of the capsule). This makes these NPs easier to be visualized with optical microscopy, and it requires lower laser powers for photothermal heating (as there is a collective effect). Last‐but‐not‐least local intracellular excitation is facilitated at the location of the illuminated capsule, that is, the site of excitation can be chosen with micrometer precision. In addition, it is feasible to create a subpopulation of cells with endocytosed capsules and others which do not have capsules by controlling the amount of fed capsules per cell, whereas NPs would due to their much smaller size inevitably be taken up into all cells. Having outlined these potential advantages, the goal of our study was to demonstrate the possibility of photothermal heating of endocytosed polymer capsules with integrated plasmonic NPs as a tool to study the intra‐ and inter‐cellular spreading of calcium waves in 2d and 3d cell (co‐)cultures.

## 2 Results and Discussion

Before using endocytosed capsules with star‐shaped NPs for exciting Ca^2+^ release via photothermal heating, a number of control experiments were carried out. First, the ability to record intra‐ and inter‐cellular Ca^2+^ waves with Ca^2+^ indicator Fluo‐4 (loaded as Fluo‐4 AM) was verified. A very crude approach was used to increase [Ca^2+^]_i_. MCF‐7 cells at low (i.e., isolated cells) and at high (i.e., contact between adjacent cells) density were loaded with Fluo‐4 AM first and incubated with capsules containing undissolved CaCO_3_ cores, which were endocytosed.^[^ ^21^ ^]^ Inside endolysosomes the CaCO_3_ particles were partially dissolved due to the acidic environment, which continuously released Ca^2+^. There is also lysosomal swelling due to increased osmotic pressure^[^ ^22^ ^]^ and transient cavitation by generated CO_2_ bubbles,^[^ ^23^ ^]^ leading to rupture of the lysosomal membrane and thus release of Ca^2+^ into the cytosol. This was observed by time‐lapse imaging using Fluo‐4 fluorescence within individual cells, see Figures S8 and S9, Supporting Information. Elevated [Ca^2+^]_i_ were also observed in cells that had no internalized CaCO_3_ particles, but were positioned adjacent to a cell with Ca^2+^ levels elevated by lysosomal Ca^2+^ due to dissolution of endocytosed CaCO_3_ particles, see Figures S10 and S11, Supporting Information. This can be explained by the diffusion of Ca^2+^ via gap junctions. Clearly, this method of introducing an increase in [Ca^2+^]_i_ via dissolving CaCO_3_ particles has its cell‐biological limits, as apart from Ca^2+^ also the lysosomal content, involving digestive enzymes like proteases and lysozyme and other destructive enzymes, are released into the cytosol, which may trigger several specific and non‐specific effects.^[^ ^24^ ^]^ As this process happened spontaneously it was also uncontrollable.

Therefore, a more refined method was used. Adenosine 5′‐triphosphate (ATP) was used as a purinergic agonist and directly added to either MCF‐7 or HeLa cells. The addition of ATP caused a rapid release of Ca^2+^ from the endoplasmic reticulum (ER),^[^ ^25^ ^]^ cf. Figures S22 and S23, Supporting Information. Interestingly, all MCF‐7 cells responded to ATP, but some HeLa cells had no response. We also loaded ATP into the capsules by the post‐loading and heat‐shrinking method.^[^ ^26^ ^]^ The capsules were anchored on the outer surface of HeLa cells by reducing the incubation time to 1–2 h. Photothermal heating on the capsules released ATP, and its diffusion evoked elevated [Ca^2+^]_i_ in some nearby cells (Figures S25 and S26, Supporting Information). Note that the cavity of the capsules loaded with ATP has micrometer dimensions. While thermal degradation of some ATP close to the capsule walls with integrated Au NPs upon photothermal heating cannot be excluded, in previous work it was shown that there is a fraction of released biological molecules which can be released via photothermal heating upon retaining their biological functionality.^[^ ^20^, ^26^, ^27^ ^]^

As an additional control, the ability to stimulate the release of stored Ca^2+^ upon photothermal heating of plasmonic NPs^[^ ^15^ ^]^ was demonstrated. For this, cells were incubated with 40 µg mL^−1^ of star‐shaped Au NP (without capsules) for 24 h. After endocytosis of the NPs and Fluo‐4 AM loading, laser irradiation caused photothermal heating of the NPs, and raising Fluo‐4 fluorescence, which indicated the increase of [Ca^2+^]_i_, cf. Figures S27 and S28, Supporting Information. Live/dead viability assays based on calcein AM and ethidium homodimer‐1 staining^[^ ^17^ ^]^ were carried out to demonstrate that upon this excitation process. The vast majority of cells remain viable, cf. Figures S20 and S21, Supporting Information. While the local temperature rise upon photothermal heating can only be estimated,^[^ ^28^ ^]^ the temperature increase warranting for the opening of capsules is highly localized, which has been demonstrated by the retained biological activity of encapsulated molecules such as proteins.^[^ ^20^, ^26^ ^]^

**Figure 1:**
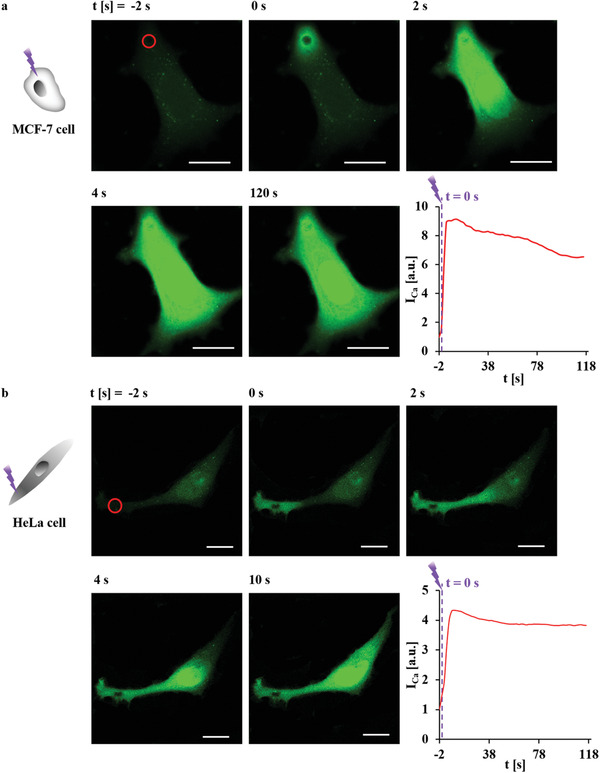
At time *t* = 0 s one capsule with embedded star‐shaped Au NPs, as endocytosed by anMCF‐7 cell(a) and HeLa cell (b) and, as indicated by the red circle, was excited with an 830 nm laser spot of *A*_laser_ = 12.56 µm^2^ spot size (20× objective, LSM880) at *P*_laser_ = 28.5 mW (at the illumination spot) for Δ*t*_laser_ = 0.039 s. Images were taken every 2 s. The scale bars represent 20 µm. The integrated fluorescence intensity of the calcium indicator Fluo‐4,*I*_Ca_, over the cross‐section of the whole cell area was normalized to that before irradiation (*t* = −2 s). *I*_Ca_ relates to the [Ca^2+^]_i_ is plotted versus time *t*. Note, *I*_Ca_ refers to fluorescence intensity and not to an ionic current. More information on this Figure is shown in Figures S29–S31, Supporting Information.

Having demonstrated Ca^2+^ release based on photothermal heating, in the following the versatility of this methodology was demonstrated. For this, cells were incubated with the capsules (with star‐shaped Au NPs in their walls) at densities which resulted in typically only 0–2 endocytosed capsules per cell. Cells were then loaded with Fluo‐4 AM and individual capsules were light‐excited (i.e., the focus of the light pointer has approximately the size of one capsule and thus capsules could be excited one by one).^[^ ^20^, ^26^, ^29^ ^]^ Experiments were first conducted by using a widefield microscope, but we found an artifact of increased Fluo‐4 fluorescence by the strong excitation light of the widefield microscope used for imaging. Thus, all the experiments were repeated using a laser scanning microscope (LSM880), where the artifact did not occur, cf. Sections S4.1,S4.2, Supporting Information. Then the local [Ca^2+^]_i_ were mapped by fluorescence imaging of Fluo‐4. **Figure** **1** and Figures S29 and S31, Supporting Information, demonstrate star‐shaped Au NP‐evoked Ca^2+^ release and its cytosolic spatiotemporal spread in a single MCF‐7 or HeLa cell. The increased fluorescence started from the irradiated region and spread to the other parts of the cell. This can be attributed to the diffusion of released free Ca^2+^, or Ca^2+^ induced Ca^2+^ release^[^ ^30^ ^]^ in other part of the cell, or mixed effects. The decay of [Ca^2+^]_i_ of a stimulated cell to its baseline has been reported to happen within 1 min,^[^ ^31^ ^]^ which was much slower in our results. Of note, the photobleaching effect under continuous imaging can be ignored by using the LSM880 set‐up due to its low excitation power (Table S1, Supporting Information). Photothermal excitation can be used multiple times, that is, it is possible to stimulate the repetitive release of Ca^2+^ by subsequent photoexcitations (Figures S30 and S32, Supporting Information).

We note that the light intensity used for photothermal heating in this study for exciting star‐shaped Au NPs was similar to that of cases where photothermal heating was used for the opening of endocytosed capsules and the surrounding lysosomal membranes for releasing encapsulated molecular cargo to the cytosol.^[^ ^26^ ^]^ A slight lowering of cytosolic pH upon photothermal heating was observed right after irradiation (Figures S33 and S34, Supporting Information), due to the release of protons from the lysosomes to the cytosol, and the pH kept constant afterwards. Meanwhile, the pH indicator SNARF‐1 gradually diffused into the irradiated lysosomes (as identified by the location of the irradiated capsules, which was clearly visible in the microscopy images), and the pH of the irradiated lysosomes remained highly acidic (Figures S33, Supporting Information), proving that photothermal opening of the lysosomal membrane is transient.^[^ ^20a^ ^]^ Also, transient changes in pH around the photoexcited capsules are a local phenomenon with a decay length of a few micrometers (Figures S34 and S37, Supporting Information). One still could argue that the change in Fluo‐4 fluorescence upon photothermal heating might be due to small changes in the cytosolic pH, as Fluo‐4 is pH‐responsive. Furthermore, change in Fluo‐4 intensity might be also due to temperature increase upon photothermal heating. However, lowering pH or raising the temperature decreased the fluorescence intensity of Fluo‐4 (Figure S7, Supporting Information). Thus, the increase in Fluo‐4 signal upon photothermal heating as shown in Figure 1 cannot be explained by pH or temperature effects, and thus clearly is attributed to the release of Ca^2+^.

**Figure 2:**
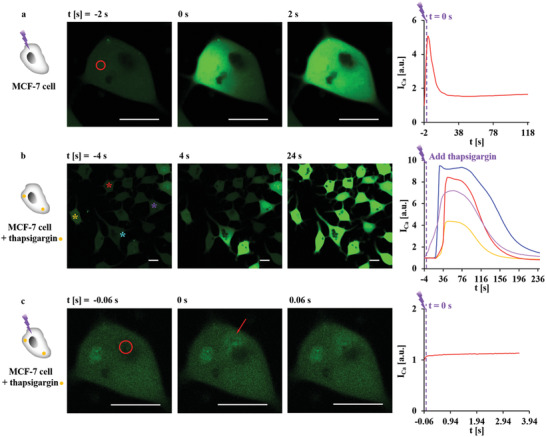
a) At time *t* = 0 s, one capsule with embedded star‐shaped Au NPs, as endocytosed by an MCF‐7 cell and as indicated by the red circle, was irradiated at 830 nm with an irradiation area of ca. 12.56 µm^2^ (20× objective, LSM880) at *P*_laser_ = 28.5 mW (at the illumination spot) for Δ*t*_laser_ = 0.039 s. Images were taken every 2 s. The integrated fluorescence intensity of the calcium indicator Fluo‐4,*I*_Ca_, (shown in green) over the cross‐section of the whole cell area was normalized to that before irradiation *t* = −2 s, and is plotted versus time *t*. *I*_Ca_ relates to the [Ca^2+^]_i_ is plotted versus time *t*. b) Thapsigargin in Ca^2+^‐free PBS was rapidly added to the cells at a final concentration of 500 × 10^−9^ m, and the change in transient [Ca^2+^]_i_ was observed. Images were taken every 4 s. The cell indicated by the red box is the same cell shown in (a) and (c). c) Another capsule in the same cell was irradiated at 830 nm with an irradiation area of ca. 12.56 µm^2^ (20× objective, LSM880) at *P*_laser_ = 57 mW (at the illumination spot) for Δ*t*_laser_ = 0.004 s. Images were taken every 0.06 s. *I*_Ca_ (shown in green) over the cross‐section of the whole cell area was normalized to that before irradiation *t* = −0.06 s, and is plotted versus time *t*. The scale bars represent 20 µm. Note that the increase in [Ca^2+^]_i_ is in general weak and transient, as Ca^2+^ released from lysosomes is rapidly diluted by the surrounding medium. More images are shown in Figure S38, Supporting Information.

As the intracellular concentration of Fluo‐4 is not known, and as its fluorescence intensity *I*_Ca_ depends on the local Fluo‐4 concentration, absolute quantification of increased [Ca^2+^]_i_ was not possible. However, the magnitude of the increase in [Ca^2+^]_i_ can be compared to the increase in [Ca^2+^]_i_ upon the addition of ca. 0.165 × 10^−3^ m ATP (cf. Figures S22 and S23, Supporting Information). The massive increase of [Ca^2+^]_i_ in the whole volume of the cytosol hardly can be explained by Ca^2+^ released upon the transient burst of the photoexcited lysosomes (which has a volume much smaller than that of the cytosol). While in this report, the focus is on demonstrating the application potential of our technology, but still, some experiments trying to unravel the mechanism of the global intracellular Ca^2+^ waves have been performed. First, photoexcitation was carried out in Ca^2+^‐free extracellular medium. The same response as in Ca^2+^ medium was obtained (Figure S35, Supporting Information), and thus the increase in [Ca^2+^]_i_ upon photoexcitation is not due to influx of Ca^2+^ by ion channels in the plasma membrane or through a damaged membrane after photothermal heating. A likely source of Ca^2+^ thus is the ER. The addition of thapsigargin causes immediate Ca^2+^ depletion of the ER,^[^ ^32^ ^]^ which could be observed by increased [Ca^2+^]_i_ (Figures S36 and S38b, Supporting Information). In the next experiment, several manipulations were carried out with the same cells: i) first, a photoexcited increase in [Ca^2+^]_i_ in Ca^2+^‐free extracellular medium was observed, followed by ii) emptying of ER Ca^2+^ stores by SERCA inhibitor thapsigargin, and finally iii) photoexcitation generated very weak and transient increases in [Ca^2+^]_i_ around the lysosomes (**Figure** **2**, Figure S38, Supporting Information). This suggests that photoinduced initial release of very low amounts of Ca^2+^ from lysosomes further triggered the robust release of Ca^2+^ from the ER by Ca^2+^ induced Ca^2+^ release. A relevant mechanism has been discussed in a review:^[^ ^33^ ^]^ “A convincing view emerging from recent studies is that NAADP functions as a trigger to elicit an initial Ca^2+^ signal that is then amplified by cADPR‐ or IP3‐ dependent responses (or both) through CICR”.^[^ ^33^ ^]^ NAADP in many cells releases Ca^2+^ from lysosomes, thereby creating local Ca^2+^ signals that are amplified by Ca^2+^ release from the ER, which is discussed in a review.^[^ ^34^ ^]^ This review describes how the ER and acidic Ca^2+^ stores physically and functionally interact to generate and shape global and local Ca^2+^ signals, with particular emphasis on the two‐way dialogue between these two organelles.

**Figure 3:**
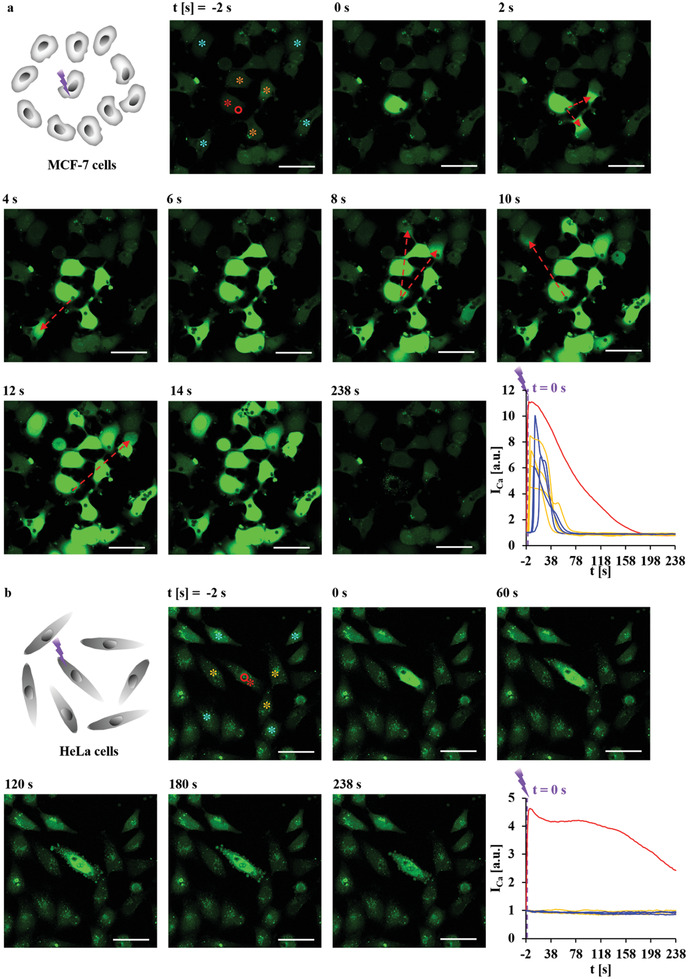
a) MCF‐7 and b) HeLa cells were seeded at densities in which cells were not in direct contact with each other. At time *t* = 0 s, one endocytosed capsule with embedded star‐shaped Au NPs, as indicated by the red circle, was irradiated at 830 nm with an irradiation area of *A*_laser_ = 12.56 µm^2^ (20× objective, LSM880) at *P*_laser_ = 71.25 mW (at the illumination spot) for Δ*t*_laser_ = 0.039 s. The scale bars represent 50 µm. Images were taken every 2 s. The integrated fluorescence intensity of the calcium indicator Fluo‐4,*I*_Ca_, over the cross‐section of the whole cell area was normalized to that before irradiation (*t* = −2 s), which relates to the [Ca^2+^]_i_, is plotted versus time *t*. The colors of the curves indicate the cells in which the Fluo‐4 intensities were measured, as given by the color of the stars labeling the respective cells. Red stars indicate irradiated cells. Yellow or blue stars indicate cells close to or far away from the irradiated cell. Red arrows indicate the calcium spread direction from irradiated cells to adjacent cells. More information on this Figure is shown in Figure S41, Supporting Information.

After having demonstrated a photoexcited increase in [Ca^2+^]_i_ at the single‐cell level, we subsequently studied the effect of exciting local Ca^2+^ release in individual cells on adjacent cells. For this, in an ensemble of cells, the Ca^2+^ level in one cell was increased by photothermal heating, while the [Ca^2+^]_i_ in adjacent cells were measured via recording the Fluo‐4 intensities in the respective cells. First, cells were seeded that there was no physical contact between adjacent cells. For MCF‐7 and HeLa cells, after excitation at low power *P*_laser_ = 34.2 mW for Δ*t*_laser_ = 0.039 s, no response in the adjacent cells was observed (Figure S40, Supporting Information). Interestingly, after excitation at high power *P*_laser_ = 71.25 mW for Δ*t*_laser_ = 0.039 s, the photothermal release of Ca^2+^ in one MCF‐7 cell, as mapped by the increase in the Fluo‐4 intensity, also caused a delayed response in adjacent cells (**Figure** **3**a and Figure S41a, Supporting Information). The Fluo‐4 intensity of those cells rose within a few to tens of seconds after the photothermal excitation. The delayed response clearly rules out that Ca^2+^ in adjacent cells (which also had internalized capsules) had been released due to photothermal heating effects, as this would have caused an instantaneous response. The response was both distance‐ and direction‐dependent. Generally, cells closer to excited cells responded earlier. For those cells with a similar distance to the excited cell, the response was first detected in cells that were located opposite to the excited region, in agreement with other reports.^[^ ^31a^ ^]^ We also noticed that the part of responding cells close to the irradiated cells became bright first, and later fluorescence became homogeneous in the whole cells, as indicated by the red arrow in Figure 3a and Figure S41a, Supporting Information. Thus, the most likely reason is intercellular communication by secreted chemical messenger molecules. Indeed, it is known that cells may secrete ATP upon stimulation,^[^ ^31^, ^35^ ^]^ which acts as a paracrine transmitter of intercellular Ca^2+^ waves to excite increased [Ca^2+^]_i_ in other cells,^[^ ^36^ ^]^ which has been also demonstrated here in the above described control experiments, cf. Figures S22 and S23, Supporting Information. To analyze the role of ATP (there are also other paracrine messengers which might be involved) stimulation was performed in the presence of 60 U mL^−1^ apyrase, an ATP hydrolyzing enzyme. Figures S42 and S43, Supporting Information, show that in this case, much less adjacent cells (i.e., only 0–2 cells) responded, demonstrating that ATP is the major paracrine mediator under our experimental conditions. Thus, the explanation of the findings in Figure 3 is that the photoexcited cells release ATP, which then triggers the release of Ca^2+^ in adjacent cells. Interestingly, only 0–1 adjacent HeLa cell responded (Figure 3b, Figure S41b, Supporting Information) at *P*_laser_ = 71.25 mW, suggesting that MCF‐7 was more sensitive to ATP than HeLa cells. ATP release is reported to be significantly affected by gap junctions, as extensive expression of gap junctions after gene transfection in gap junction‐deficient cell can significantly enhance ATP release to the 5–15 folds.^[^ ^35^ ^]^ HeLa cell is a gap junction‐deficient cell line,^[^ ^37^ ^]^ but MCF‐7 forms complete gap junctions,^[^ ^38^ ^]^ which may explain the difference in the response of surrounding cells.

**Figure 4:**
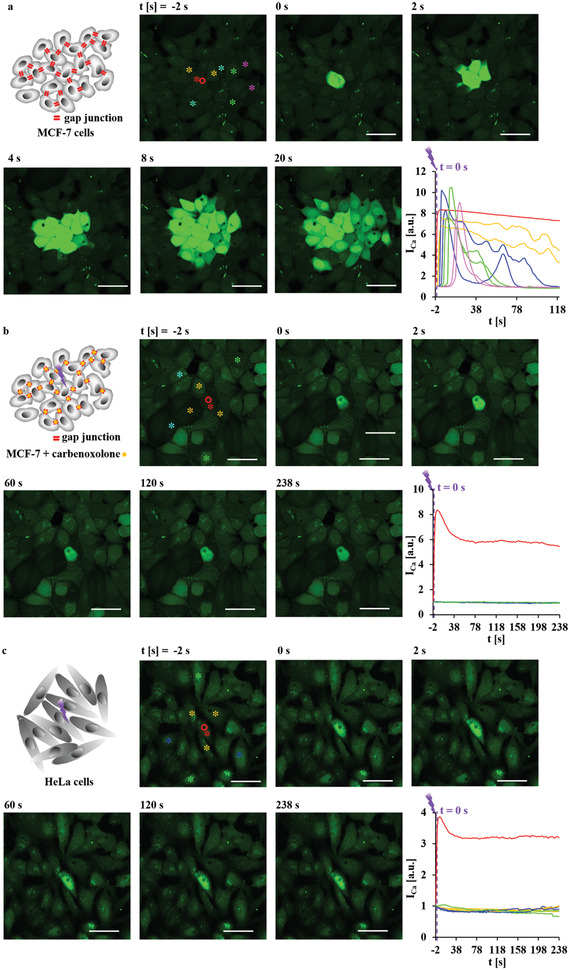
a,b) MCF‐7 and c) HeLa cells were seeded at densities in which cells formed an almost confluent layer, enabling physical contact in‐between adjacent cells. At time *t* = 0 s, one endocytosed capsule with embedded star‐shaped Au NPs, as indicated by the red circle, was excited with an 830 nm laser spot of *A*_laser_ = 12.56 µm^2^ spot size (20× objective, LSM880) at *P*_laser_ = 34.2 mW (at the illumination spot) for Δ*t*_laser_ = 0.039 s. MCF‐7 cells were imaged in absence (a) or presence (b) of 200 × 10^−6^ m carbenoxolone, which blocks gap junctions. The scale bars represent 50 µm. The integrated fluorescence intensity of the calcium indicator Fluo‐4,*I*_Ca_, over the cross‐section of the whole cells labeled with stars was normalized to that before irradiation (*t* = −2 s). It relates to the [Ca^2+^]_i_ in these cells, here plotted versus time *t*. The colors of the curves indicate the cells in which the Fluo‐4 intensity was measured, as given by the color of the stars labeling the respective cells. Red stars indicate irradiated cells. Yellow, blue, green, and pink stars indicate cells with increasing distance from the irradiated cell. More information on this Figure is shown in Figures S44–S47, Supporting Information.

Whereas in Figure 3 intercellular signaling by diffusing messenger molecules (i.e., ATP) was mapped, in **Figure** **4**a, Figures S44a and S46a, Supporting Information, intercellular signaling by direct cell‐to‐cell Ca^2+^ signaling via gap junctions is shown. One MCF‐7 cell was photoexcited at low power *P*_laser_ = 34.2 mW for Δ*t*_laser_ = 0.039 s to avoid ATP secretion. There was an almost instantaneous Ca^2+^ spread also in adjacent cells, spreading across almost the whole cell layer. The spread was time‐dependent, that is, cells further away were excited later, and the spreading time was comparable to the one in Figure 3a. Confluent MCF‐7 cells are known to form gap junctions, which allow for direct diffusion of Ca^2+^ from one cell to another.^[^ ^39^ ^]^ To corroborate this hypothesis, carbenoxolone or octanol, which are known to temporally block gap junctions,^[^ ^39^, ^40^ ^]^ was added. As shown in Figure 4b, Figures S45, S46b, and S47, Supporting Information, the addition of carbenoxolone or octanol avoided the fast spreading of the Ca^2+^ wave that was initiated by photothermal heating in one cell to adjacent cells. Additional control experiments were performed with HeLa cells, which do not have gap junctions.^[^ ^37^ ^]^ In Figure 4c, Figures S44b and S46c, Supporting Information, Ca^2+^ spreading via gap junctions was not observed.

**Figure 5:**
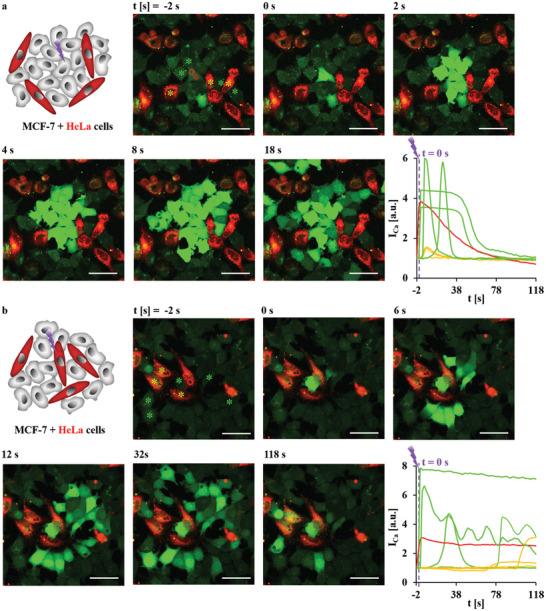
MCF‐7 cells were seeded in co‐culture with HeLa cells at densities in which cells formed an almost confluent layer, enabling physical contact in‐between adjacent cells. The HeLa cells were labeled with CellTracker Deep Red, and thus they were red fluorescent. a,b) At time *t* = 0 s, one endocytosed capsule with embedded star‐shaped Au NPs in anMCF‐7 cell (a) or HeLa cell (b) as indicated by the red circle was excited at 830 nm with an irradiation area of *A*_laser_ = 12.56 µm^2^ (20× objective, LSM880) at *P*_laser_ = 71.25 mW (at the illumination spot) for Δ*t*_laser_ = 0.039 s. Images were taken every 2 s. The scale bars represent 50 µm. The integrated fluorescence intensity of the calcium indicator Fluo‐4,*I*_Ca_, over the cross‐section of the whole cells labeled with stars was normalized to that before irradiation (t = −2 s), and relates to the [Ca^2+^]_i_ in these cells. The fluorescence intensity of Fluo‐4 is plotted versus time *t*. The colors of the curves indicate the cells in which the Fluo‐4 intensities were measured, as given by the color of the stars labeling the respective cells. The red star indicates the irradiated cell. Green stars indicate adjacent MCF‐7 cells. Yellow stars indicate the adjacent HeLa cells. More information on this Figure is shown in Figures S48a and S49, Supporting Information.

**Figure 6:**
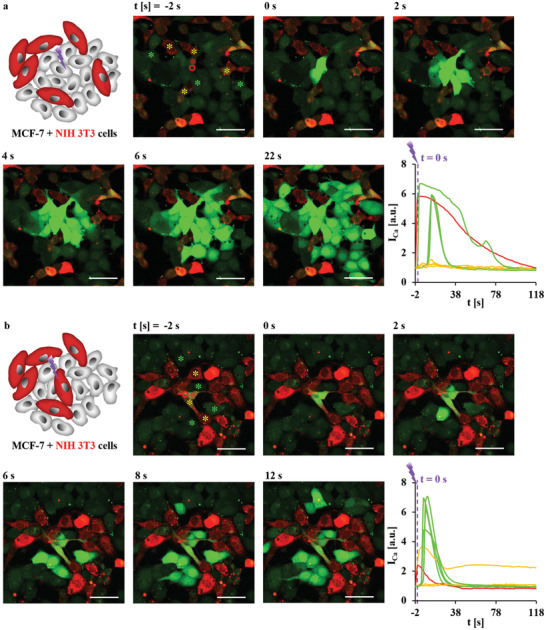
MCF‐7 cells were seeded in co‐culture with NIH 3T3 cells at densities in which cells formed an almost confluent layer, enabling physical contact in‐between adjacent cells. The NIH 3T3 cells were labeled with CellTracker Deep Red, and thus they were red fluorescent. a,b) At time *t* = 0 s one endocytosed capsule with embedded star‐shaped Au NPs in anMCF‐7 cell (a) or NIH 3T3 cell (b), as indicated by the red circle was excited at 830 nm with an irradiation area of *A*_laser_ = 12.56 µm^2^ (20× objective, LSM880) at power *P*_laser_ = 71.25 mW (at the illumination spot) for Δ*t*_laser_ = 0.039 s. Images were taken every 2 s. The scale bars represent 50 µm. The integrated fluorescence intensity of the calcium indicator Fluo‐4,*I*_Ca_, over the cross‐section of the whole cells labeled with stars was normalized to that before irradiation (*t* = −2 s), and is plotted versus time *t*. The colors of the curves indicate the cells in which the Fluo‐4 intensities were measured, as given by the color of the stars labeling the respective cells. The red star indicates the irradiated cell. Green stars indicate adjacent MCF‐7 cells. Yellow stars indicate adjacent NIH 3T3 cells. More information on this Figure is shown in Figures S48b and S50, Supporting Information.

Figures 1, 2, 3, 4 display that with our methodology it is possible to locally excite the release of Ca^2+^ by photothermal heating at a subcellular level, and that the effect of this on [Ca^2+^]_i_ of adjacent cells can also be observed. This methodology can be applied to more complicated cell culture systems as the so far described 2D monocultures, that is, it can be extended to cell co‐cultures and also to 3D cell cultures. As displayed in **Figure** **5**, mixtures of two different cell lines have been seeded on cell culture substrates to form 2D co‐cultures. To identify the types of individual cells within co‐cultures, one cell line was loaded by intracellular labels.^[^ ^41^ ^]^ Here, we labeled HeLa (epithelial cell line) and NIH 3T3 cells (fibroblast cell line) with CellTracker Deep Red, whereas MCF‐7 cells were not labeled. Cells fluorescence in red (and after photothermal excitation also in green) thus corresponded to HeLa or NIH 3T3 cells, whereas cells that were only fluorescent after photothermal excitation in green were identified as MCF‐7 cells. In Figure 5, **Figure** **6**, and Figures S48–S50, Supporting Information, co‐cultures of MCF‐7/HeLa and MCF‐7/NIH 3T3 cells are shown, in which Ca^2+^ release was triggered in one cell by photothermal heating at high power *P*_laser_ = 71.25 mW for Δ*t*_laser_ = 0.039 s. Consistent with Figures 3 and 4, the spreading of a Ca^2+^ wave occurred in adjacent MCF‐7 cells, but seldomly in HeLa or NIH 3T3 cells. When MCF‐7 cells were excited (Figures 5a and 6a, Figures S48, S49a, and S50a, Supporting Information), Ca^2+^ signaling could be spread to adjacent MCF‐7 cells via gap junctions and secreted ATP. MCF‐7 cells cannot form gap junctions with either HeLa cells, which do not express connexins, or with NIH 3T3 cells, because gap junctions cannot be formed between epithelial and fibroblast cell lines.^[^ ^42^ ^]^ Therefore, direct Ca^2+^ spread in different types of cells through gap junctions was not possible. Only 0–2 HeLa or NIH 3T3 cells responded weakly, which was attributed to the diffusion of secreted ATP from the excited MCF‐7 cell. Interestingly, when HeLa or NIH 3T3 cells were excited (Figures 5b and 6b, Figures S49b and S50b, Supporting Information), similar results were observed. Again, the diffusion of secreted ATP from excited HeLa or NIH 3T3 cells triggered Ca^2+^ increase in adjacent MCF‐7 cells. Only a few HeLa or NIH 3T3 cells responded weakly. This data also indicates that MCF‐7 cells are more sensitive to ATP than HeLa and NIH 3T3 cells, consistent with the results described above (Figure 3b, Figure S41b, Supporting Information).

**Figure 7:**
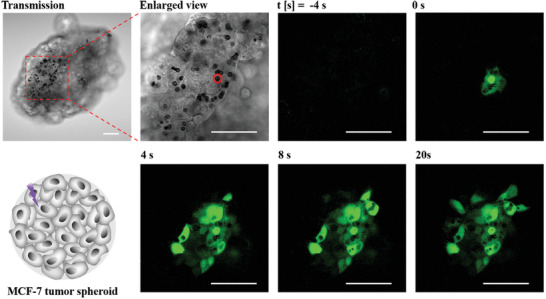
MCF‐7 cells with capsules with embedded star‐shaped Au NPs were seeded in culture to form 3D cell spheroids. At time *t* = 0 s, one capsule as indicated by the red circle was excited at 830 nm with an irradiation area of *A*_laser_ = 43.6 µm^2^ (20× objective, LSM880) at the power of *P*_laser_ = 114 mW (at the illumination spot) for Δ*t*_laser_ = 0.175 s. Images were taken every 4 s. The spread of the calcium wave to adjacent cells can be observed by the increase in Fluo‐4 fluorescence of the respective cells. The scale bars represent 50 µm. More information on this Figure is shown in Figures S52–S55, Supporting Information.

Controlled Ca^2+^ spread in tumor spheroids has been seldomly reported.^[^ ^8b^ ^]^ Controlled excitation with single‐cell precision inside tumor spheroids without disturbing the nearby cells cannot be realized by conventional electrical, chemical, and mechanical approaches. In contrast, **Figure** **7**, and Figures S52–S55, Supporting Information, show that our methodology is also applicable for 3D cell cultures. Cell spheroids were formed out of MCF‐7 cells,^[^ ^43^ ^]^ cf. Section S7.1, Supporting Information. Hereby the cells were loaded with the capsules before forming the spheroids. Because of the limited staining of Fluo‐4 in the center of the spheroids, only cells not very deep inside the spheroid were excited. At the desired time, one single capsule can be irradiated with a light pointer, leading to the photothermally stimulated release of Ca^2+^. The spread of this Ca^2+^ wave to adjacent cells can then be observed by imaging of the Fluo‐4 fluorescence in those cells, which are similar to the results of physically contacted cells.

## 3 Outline

Heat‐triggered Ca^2+^ signaling has also been reported by others as an alternative to optogenetics. Alternating magnetic field heating of iron oxide NPs,^[^ ^44^ ^]^ photothermal heating of polymeric NPs,^[^ ^45^ ^]^ or plasmonic NPs^[^ ^46^ ^]^ could stimulate thermosensitive cation channels, that is, the transient receptor potential vanilloid type 1 (TRPV1), on the outer surface of neuronal cells. Local heating of TRPV1 will transiently induce exogenous Ca^2+^ influx from the surrounding medium. In those cases, NPs need to bind to the cell membrane close to the ion channels via electrostatic or hydrophobic interaction, or even need to specifically bind to TRPV1 by conjugating them with corresponding antibodies. Our strategy here is to release Ca^2+^ from endogenous stores, and thus this method could be applied to TRPV1‐negative cells, for example, HeLa cells.^[^ ^45^ ^]^

Furthermore, light‐triggered Ca^2+^ signaling can also be realized by the release of biomolecules (e.g., ip_3_) from plasmonic liposomes.^[^ ^42^, ^47^ ^]^ However, the undesired premature release of biomolecules from liposomes^[^ ^47^ ^]^ imposes hurdles for long‐term experiments, in which probably Ca^2+^ signaling would be evoked without laser irradiation via leaching. The release is also irreversible. Non‐degradable polymeric capsules are highly stable in cells at least for several days^[^ ^48^ ^]^ and leakage is not our concern as no biological molecules are encapsulated here, which makes this approach potentially suitable for long‐term monitoring.

One potential application of our approach would be a combination with Ca^2+^ mediated gene expression. Ca^2+^‐sensitive promoters could be placed upstream of reporter genes.^[^ ^44^, ^49^ ^]^ After cells are transfected by those genes, the increase of [Ca^2+^]_i_ can active gene expression. Therefore, it would be possible to active gene expression in multiple cells upon one single irradiation on the capsules in one single cell. In this way, a single input signal would be cascaded and amplified in a biological system.

This platform could be also interesting for drug screening. Ca^2+^ signaling, and especially Ca^2+^ spreading via gap junctions, is used in tissues under physiological conditions, for example, in cardiac ventricular myocytes, to orchestrate contraction of heart ventricles. In cancer cells, Ca^2+^ signaling has emerged as a process that enhances proliferation and tumor development.^[^ ^50^ ^]^ Thus, the method developed here might be used as a screening system to detect compounds interfering with Ca^2+^ spreading via gap junctions, thereby generating new therapeutic possibilities for cancer cells depending on cell‐to‐cell Ca^2+^ signaling. This is in particular interesting since Ca^2+^ signaling in Fluo‐4‐loaded cells can be imaged in 96‐ and 386‐well plates using high content imaging applications, for example, the PerkinElmer Opera system.

One limitation of our approach is that capsules can hardly penetrate into a preformed biological organ/tissue. For example, capsules can only be endocytosed by the cells on the surface of a preformed tumor spheroid. To make capsules deep inside the tumor spheroid, the cells must take up capsules before forming 3d structure. Thus, delivery of the capsules into tumors would not be possible by classical targeting, but might be possible by preloading of capsules into stem cells which then would act as tumor‐homing transporters.^[^ ^51^ ^]^ While reaching locations deep inside tissue with the capsules is an issue, the optical stimulation allows for near‐infrared stimulation inside tissue.

## 4 Conclusions

We have demonstrated a controllable and easy‐to‐operate approach to probe intra‐ and inter‐cellular spreading of Ca^2+^ waves in 2d and 3d cell (co‐)cultures through photothermal heating of endocytosed polymer capsules integrated with plasmonic NPs. Capsules can be directly visualized with optical microscopy, allowing for precise laser irradiation at defined positions. We characterized the Ca^2+^ spread in irradiated cells and nearby cells at different cell densities, upon irradiating only one cell. For MCF‐7 cells, Ca^2+^ can rapidly spread to interconnected cells by gap junctions or in those cells without direct contact by responding to ATP from stimulated cells. For HeLa cells, in most cases, an increase in [Ca^2+^]_i_ only occurred in irradiated cells, probably due to their low ATP sensitivity. Heterocellular Ca^2+^ signaling between MCF‐7/HeLa or MCF‐7/NIH 3T3 happened mostly in MCF‐7 cells, and to a very low extent in HeLa or NIH 3T3 cells, no matter which types of cells were excited. Ca^2+^ can also spread in a 3D tumor spheroid of MCF‐7 cells.

## 5 Experimental Section

Capsules with star‐shaped plasmonic NPs integrated into their polymer shell were synthesized by adopting previously published protocols.^[^ ^29^ ^]^ The protocols to optimize the physicochemical and photothermal properties of these capsules, as well as their biocompatibility, are given in Sections S1.7 and S4.3, Supporting Information. Due to their micrometer size, these capsules could be conveniently imaged with optical microscopy. 2D and 3D (co‐) cell cultures were done with MCF‐7, HeLa, and NIH 3T3 cells, see Sections S5,S6,S7.1, Supporting Information. Once cells were exposed to capsules, they were internalized via endocytosis and were thus located in endosomes/lysosomes, which has been demonstrated in previous studies by: i) transmission electron microscopy, ii) confocal microscopy together with immunostaining of endosomes/lysosomes, iii) recording the acidic pH of endosomes/lysosomes around the capsules after internalization, and iv) using molecular blockers for endocytosis.^[^ ^21^, ^52^ ^]^ Then, after capsule internalization, [Ca^2+^]_i_ was analyzed by use of the Ca^2+^ indicator fluorophore Fluo‐4 AM,^[^ ^47^ ^]^ see Section S3.2, Supporting Information. Photothermal heating, as well as imaging of the cells and the [Ca^2+^]_i_ was performed with Zeiss Axiovert 200 M wide‐field microscope equipped with a Rapp OptoElectronic DL‐830 laser generator,^[^ ^20^, ^29^ ^]^ and with a Zeiss confocal laser scanning microscope LSM880, see Section S4.1, Supporting Information.^[^ ^26^ ^]^

## Conflict of Interest

The authors declare no conflict of interest.

## Supporting information

Supporting Information

## Data Availability

All data are presented in the supporting information.
