# Supplementary material for: Stimulation of Local Cytosolic Calcium Release by Photothermal Heating for Studying Intra‐ and Intercellular Calcium Waves
Source: Adv Mater. 2021 May 5;33(24):2008261. doi: 10.1002/adma.202008261 (PMC11469046; doi:10.1002/adma.202008261)
Supplement: Supplementary file 1 — Supporting Information [file ADMA-33-2008261-s001.pdf]

© 2021 Wiley-VCH GmbH

ADVANCED MATERIALS

Supporting Information

for *Adv. Mater.,* DOI: 10.1002/adma.202008261

Stimulation of Local Cytosolic Calcium Release by Photothermal Heating for Studying Intra- and Intercellular Calcium Waves

*Dingcheng Zhu, Lili Feng, Neus Feliu, Andreas H. Guse, and Wolfgang J. Parak**

Stimulation of local cytosolic calcium release by photothermal heating for studying intra- and inter-cellular calcium waves.

Dingcheng Zhu[1,2], Lili Feng[1], Neus Feliu[1,3], Andreas Guse[4], Wolfgang J. Parak[1,5*]

[1]Fachbereich Physik, CHyN, Universität Hamburg, Hamburg, Germany
[2]College of Material, Chemistry and Chemical Engineering, Hangzhou Normal University, Hangzhou, PR China
[3]CAN, Fraunhofer Institut, Hamburg, Germany
[4]Universitätsklinikum Hamburg Eppendorf (UKE), Hamburg, Germany
[5]National Engineering Center for Nanotechnology (NECN), Shanghai, China
[*]corresponding author: wolfgang.parak@uni-hamburg.de

Supporting Information

1. Synthesis and characterization of Au NPs, $CaCO_3$ cores, and polymer capsules with integrated Au NPs 3
 1.1 Synthesis protocols of Au NPs 3
 1.2 Structural characterization of Au NPs 4
 1.3 Photophysical characterization of Au NPs 4
 1.4 Colloidal characterization of Au NPs 5
 1.5 Concentration determination of Au NPs 6
 1.6 Synthesis protocol of $CaCO_3$ cores 7
 1.7 Synthesis procedure of polymer capsules with integrated Au NPs 7
 1.8 Structural characterization of polyelectrolyte capsules with integrated star-shaped Au NPs 8
 1.9 Colloidal characterization of capsules with integrated Au NPs 10
2. Cell models 11
 2.1 Cell culture 11
 2.2 Seeding geometries 11
3. Calcium detection by Fluo-4 13

3.1 Response of Fluo-4 to calcium, pH, and temperature ... 13
3.2 Protocol for detection of cytosolic calcium concentrations with Fluo-4 ... 14
3.3 Measuring cytosolic calcium increase upon delivery of $CaCO_3$ containing capsules in an isolated single cell model ... 15
3.4 Measuring intercellular calcium signaling upon delivery of capsules containing Cy5-$CaCO_3$ cores in interconnected cells ... 19
4. Photothermal heating of capsules ... 20
4.1 Microscopy set-up ... 20
4.2 Photothermal heating of internalized capsules ... 26
4.3 Probing for damage to cells upon photothermal heating ... 31
5. Triggering cytosolic calcium release ... 35
5.1 Triggering cytosolic calcium release by adding extracellular ATP ... 35
5.2 Triggering cytosolic calcium release by local photothermal heating of encapsulated star-shaped Au NPs ... 40
6. Measuring cytosolic calcium increase upon photothermal heating of polymer capsules with integrated star-shaped Au NPs ... 44
6.1 Cytosolic calcium increase in isolated single MCF-7 or HeLa cells ... 44
6.2 pH change in cytosol and lysosome in isolated single MCF-7 cells after photothermal heating ... 49
6.3 Preliminary experiments for investigating the origin of cytosolic calcium increase upon photothermal heating ... 55
6.4 Cytosolic calcium increase in MCF-7 or HeLa cells which were not in direct contact with each other ... 63
6.5 Cytosolic calcium increase in MCF-7 or HeLa cells which were physically in contact with adjacent cells ... 71
6.6 Cytosolic calcium increase in co-cultured MCF-7/HeLa or MCF-7/NIH 3T3 cells which were physically in contact with adjacent cells ... 79
7. Measuring cytosolic calcium increase in three-dimensional tumor spheroids ... 85
7.1 Spheroid culture ... 85
7.2 Cytosolic calcium increase in tumor spheroids after photothermal heating ... 86
8. MATLAB code ... 93
9. References ... 96

1. Synthesis and characterization of Au NPs, $CaCO_3$ cores, and polymer capsules with integrated Au NPs

1.1 Synthesis protocols of Au NPs
1.2 Structural characterization of Au NPs
1.3 Photophysical characterization of Au NPs
1.4 Colloidal characterization of Au NPs
1.5 Concentration determination of Au NPs
1.6 Synthesis protocol of $CaCO_3$ cores
1.7 Synthesis procedure of polymer capsules with integrated Au NPs
1.8 Structural characterization of polymer capsules with integrated Au NPs
1.9 Colloidal characterization of polymer capsules with integrated Au NPs

1.1 Synthesis protocols of Au NPs

Star-shaped Au nanoparticles (NPs) were synthesized following a reported procedure by a seed-mediated growth method with a minor modification [1]. To prepare the seed Au NPs, 15 mL of 1% (w/v) tri-sodium citrate dihydrate (#3580.4, Carl Roth) aqueous solution was added to 96 mL of boiling water under vigorous stirring. 4 mL of 25 mM $HAuCl_4 \cdot 3H_2O$ (#520918-5G, Sigma Aldrich) was added immediately. After 15 min of boiling, the solution was cooled down to room temperature by removing the heating mantle, and it was then filtered by a 0.22 µm Millipore membrane.

To prepare star-shaped Au NPs , 2 mL of 25 mM $HAuCl_4 \cdot 3H_2O$, 120 µL of 1 M HCl (#4625.1, Carl Roth), and 950 µL of the above citrate-stabilized seed solution was added to 99 mL of MilliQ water at room temperature. After stirring for 1 min, 1 mL of freshly prepared 2 mM $AgNO_3$ (#204390, Sigma Aldrich) and 1 mL of freshly prepared 100 mM ascorbic acid (#33034, Sigma Aldrich) were added simultaneously. The solution was stirred for 30 s, and its color rapidly turned from light red to dark blue. The solution was cooled in an ice bath to stop the

reaction. 2.0 mL of 10 mg/mL $HSC_2H_4CONH\text{-}PEG_{3k}\text{-}OC_3H_6COOH$ (M_w = 3,000 Da, #13300-4-32, Rapp Polymer) and 250 µL of 2 M NaOH (#6771.1, Carl Roth) aqueous solution was added for ligand exchange. After 40 min incubation in the ice bath, the mixture was reacted at room temperature overnight. The solution was centrifuged at 8,000 rpm for 5 min, the supernatant was discarded, and 40 mL of MilliQ water was added and the Au NPs were redispersed. This process was repeated for 3 times. In the last step, the star-shaped Au NPs were suspended in 15 mL (not 40 mL) of water and were stored at 4 °C before use. Of note, the star-shaped Au NPs from different batches may have different plasmon absorption wavelengths maxima at different wavelength λ_{max}, even though the same protocol was used. The amount of seed can be adjusted to tune λ_{max}.

1.2 Structural characterization of Au NPs

To prepare samples for transmission electron microscopy (TEM) measurements, 50-mesh copper grids were immersed in star-shaped Au NP solution for 2 min, and the excess solution was removed by filter paper. Grids were dried in the air overnight before observation. Images were taken with a JEM-1200EX TEM. The geometry and the shape of the star-shaped Au NPs is shown in **Figure SI.1.**

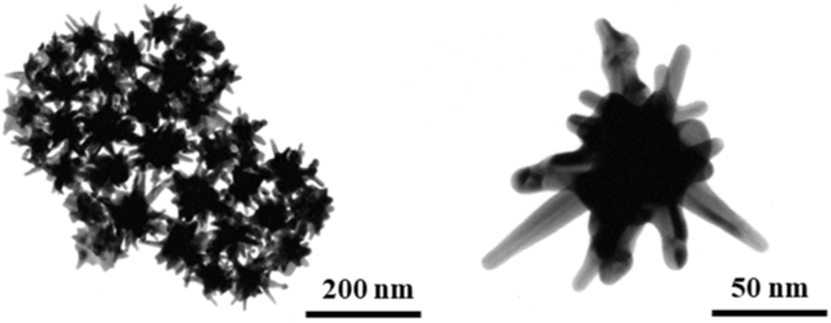

Figure SI.1 TEM images of PEGylated star-shaped Au NPs .

1.3 Photophysical characterization of Au NPs

UV-Vis absorption spectra of the Au seeds and star-shaped Au NPs in aqueous solution are shown in **Figure SI.2**.

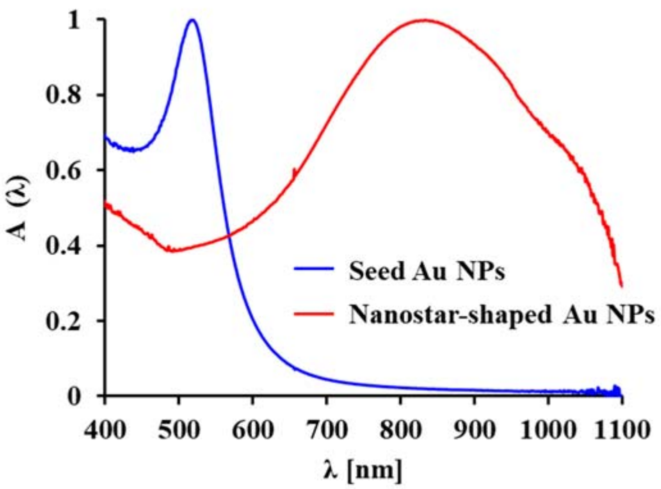

Figure SI.2 Normalized UV-Vis absorption spectra A(λ) of seed Au NPs and star-shaped Au NPs in aqueous solution. The seed Au NPs had the absorption peak at λ_{max} = 518 nm. The surface plasmon resonance peaks of the here shown star-shaped Au NPs was at λ_{max} = 834 nm.

1.4 Colloidal characterization of Au NPs

Hydrodynamic diameters d_h and zeta-potentials ξ of the seed Au NPs and star-shaped Au NPs were measured on a Zetasizer Nano (Malvern Instrument Ltd.) at 25 °C. 500 μL of NP samples were used for size measurement, and 800 μL of NP samples were diluted with 200 μL of Na_2HPO_4-NaH_2PO_4 buffer solution (200 mM, pH 7.4) for zeta-potential measurements. Data were measured for 3 times. The resulting data from one measurement are shown in **Figure SI.3.**

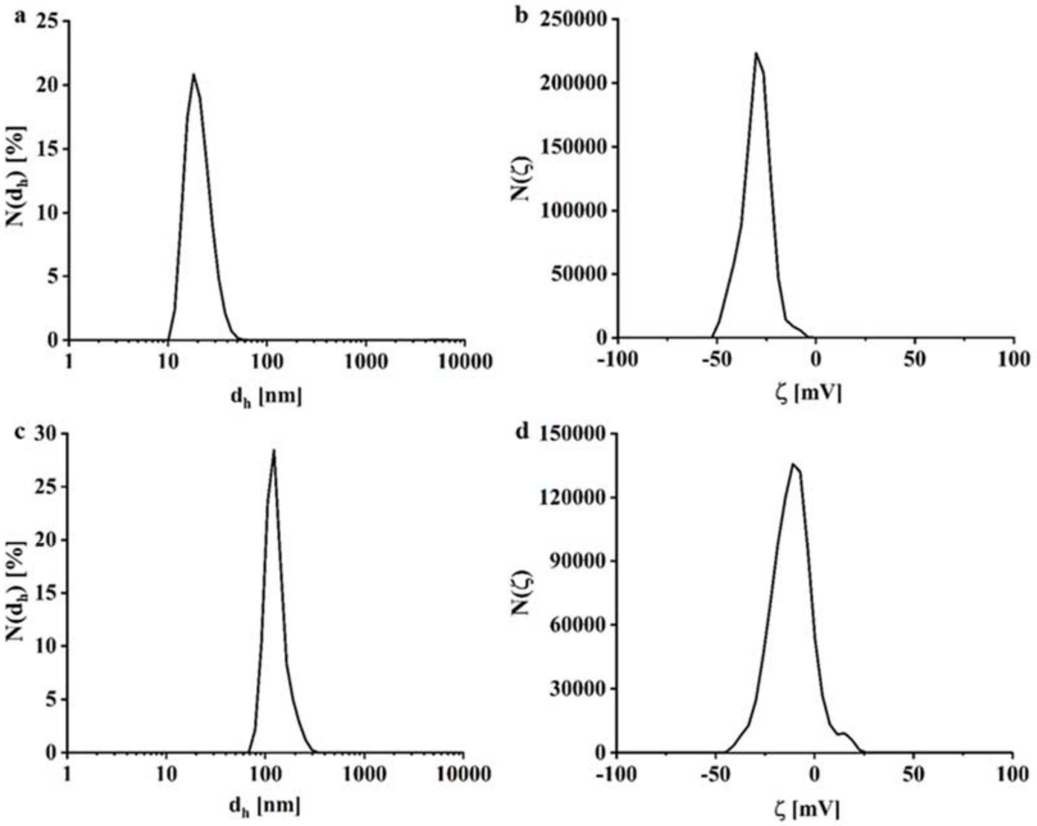

Figure SI.3 Colloidal characterizations of a,b) seed Au NPs and c,d) star-shaped Au NPs. a,c) Number distribution N(d_h) of the hydrodynamic diameter d_h of a) seed Au NPs and c) star-shaped Au NPs as derived from dynamic light scattering (DLS) measurements, recorded in water. The mean hydrodynamic diameters were d_h = 18.2 ± 1.1 nm and 113.4 ± 1.9 nm, respectively. b,d) Zeta-potential distribution N(ξ) of b) seed Au NPs and d) star-shaped Au NPs in 40 mM Na_2HPO_4-NaH_2PO_4 buffer solution (pH 7.4). The mean zeta-potentials were ξ = -30.6 ± 2.6 mV and -14.0 ± 1.9 mV, respectively.

1.5 Concentration determination of Au NPs

The mass concentration of star-shaped Au NPs was determined by calculating the amount of elemental gold using an inductively coupled plasma mass spectrometry (ICP-MS) 7700 setup from Agilent with an integrated auto sampler. 50 μL of diluted Au NP solution (30:1, 60:1, 120:1 and 240:1) of the stock solution were digested in 150 μL of freshly prepared *aqua regia* in a hood at room temperature overnight. Afterwards, 1.8 mL of 0.74 % (w/v) diluted HCl was added. The corresponding calibration curve of elemental gold was obtained by using an elemental gold standard (1 mg/mL) diluted consecutively from 2500 parts per billion (ppb) to

10 ppb. The calculated mass concentration of star-shaped Au NPs (in terms of elemental gold) was 0.244 ± 0.010 mg/mL.

1.6 Synthesis protocol of $CaCO_3$ cores

Spherical porous $CaCO_3$ cores with a diameter of 4.5-5 µm were synthesized by adding 630 µL of $CaCl_2 \cdot 2H_2O$ (0.33 M, #223506, Sigma Aldrich) solution into 630 µL of Na_2CO_3 (0.33 M, #S7795, Sigma Aldrich) solution under stirring at 1,000 rpm for 30 s. Afterwards, the mixture was left undisturbed, and $CaCO_3$ growth was stopped after 2.5 min. The cores were centrifuged, and washed with water for 3 times.

SNARF- or Cy5-labelled dextran containing $CaCO_3$ cores (SNARF-$CaCO_3$ or Cy5-$CaCO_3$) were synthesized as followings. Briefly, 350 µL of 2 mg/mL SNARF-labelled dextran (M_w: 70 kDa, anionic, #D3304, ThermoFisher) or Cy5-labelled dextran (M_w: 10 kDa, anionic. #D22914, ThermoFisher) was added into 630 µL of $CaCl_2 \cdot 2H_2O$ (0.33 M) solution prior to addition of 630 µL of Na_2CO_3 (0.33 M) solution. All the other steps are the same as described above.

1.7 Synthesis procedure of polymer capsules with integrated Au NPs

Non-biodegradable polyelectrolyte microcapsules were prepared by depositing alternating layers of negatively charged poly(sodium 4-styrenesulfonate) (PSS, M_w = 70 kDa, #243051, Sigma Aldrich, 10 mg/mL in 0.05 M NaCl, pH 6.5,) and positively charged poly(allylamine hydrochloride) (PAH, M_w = 56 kDa, #283223, Sigma Aldrich, 10 mg/mL in 0.05 M NaCl, pH 6.5)[2]. $CaCO_3$ cores were first resuspended in 1 mL of PSS solution, sonicated for 3 min, and shaken for 10 min. Afterwards the particles were centrifuged at 2,500 rpm for 5 min, the supernatant was removed, and 2 mL of water was added. This washing step was repeated for 3 times. This procedure was also applied to the PAH layer. After assembly of two bilayers, i.e. $CaCO_3$/(PSS/PAH)$_2$, 1.5 mL of negatively charged star-shaped Au NPs (0.37 mg) were added. The mixture was sonicated for 3 min, shaken for 10 min, and washed 3 times with MilliQ water. The adsorption of star-shaped Au NPs layer was repeated to ensure saturated surface adsorption until the supernatant was dark blue after centrifugation. Finally, capsules made of $CaCO_3$/(PSS/PAH)$_2$/Au NPs/PAH/PSS were obtained.

To remove the $CaCO_3$ core, the capsules were incubated with 1.2 mL of 0.2 M ethylenediaminetetraacetic acid aqueous solution (pH 6.5, EDTA disodium salt, #E5134, Sigma Aldrich) overnight at 4 °C. The hollow capsules were centrifuged at 1600 rpm for 3 min, the supernatant was removed, and 2 mL of water was added. This washing step was repeated for 3 times. Finally, the capsules were stored at 4 °C before use. SNARF-$CaCO_3$ or Cy5-$CaCO_3$ capsules containing undissolved cores whose structure was $CaCO_3$@$(PSS/PAH)_3$ were prepared in the same method as described above in but without the core removal step.

To encapsulate adenosine triphosphate (ATP) into capsules, the post-loading and heat shrinking method was used due to its small molecule weight [3]. Hollow capsules were incubated with 15 mg/mL of ATP aqueous solution (#A1852-1VL, Sigma Aldrich) for 1 h at room temperature. Afterwards, the capsules were heat-shrunk at 70 °C for 1 h [3]. The capsules were centrifuged at 1,600 rpm for 5 min, the supernatant was removed, and 2 mL of water was added. This washing step was repeated for 3 times. To avoid ATP hydrolysis, capsules were used immediately.

In a recent publication, we prepared capsules with the same structure with star-shaped Au NPs, i.e. PSS/PAH/PSS/PAH/Au NPs/PAH/PSS [3a]. In Table SI.4 in this paper, the amount of Au was calculated as 4.87 ± 0.43 pg/capsule. Despite of batch-to-batch differences, the value for the capsules used in the present study will be be similar. We want to point out that loading of cells with this amount of Au is tolerated by cells without acute toxic effects [4].

1.8 Structural characterization of polyelectrolyte capsules with integrated star-shaped Au NPs

The morphology of capsules with integrated star-shaped Au NPs is shown in **Figure SI.4.**

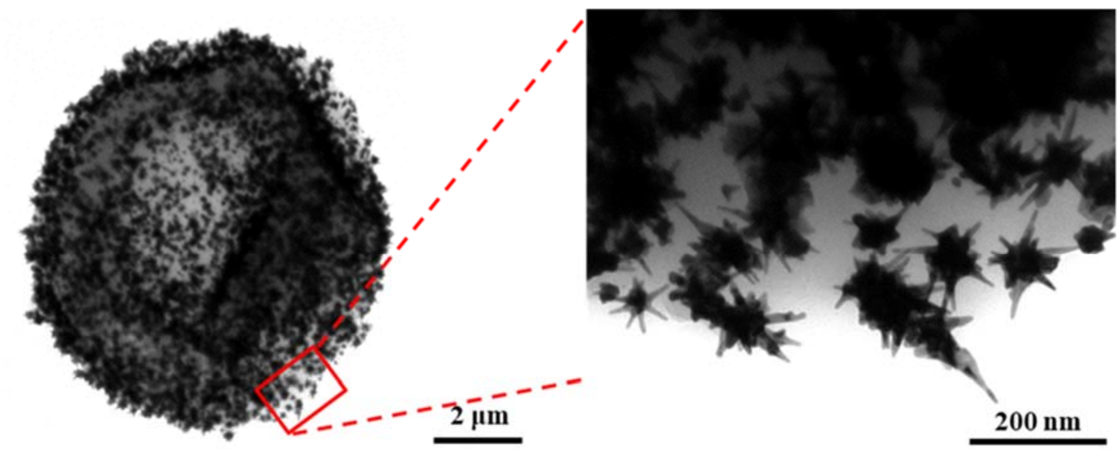

Figure SI.4 TEM images of capsules with integrated star-shaped Au NPs. Their structure was $(PSS/PAH)_2$/Au NPs/PAH/PSS.

1.9 Colloidal characterization of capsules with integrated Au NPs

Hydrodynamic diameters d_h and zeta-potentials ξ of capsules with integrated star-shaped Au NPs were measured on a Zetasizer Nano (Malvern Instrument Ltd.) at 25 °C. Capsules were diluted with water to reach a density of 2×10^6 capsules/mL. 500 µL of samples were used for size measurements, and 800 µL samples were diluted with 200 µL of Na_2HPO_4-NaH_2PO_4 buffer solution (200 mM, pH 7.4) for zeta-potential measurements. Data were measured for 3 times. The resulting data from one measurement are shown in **Figure SI.5.**

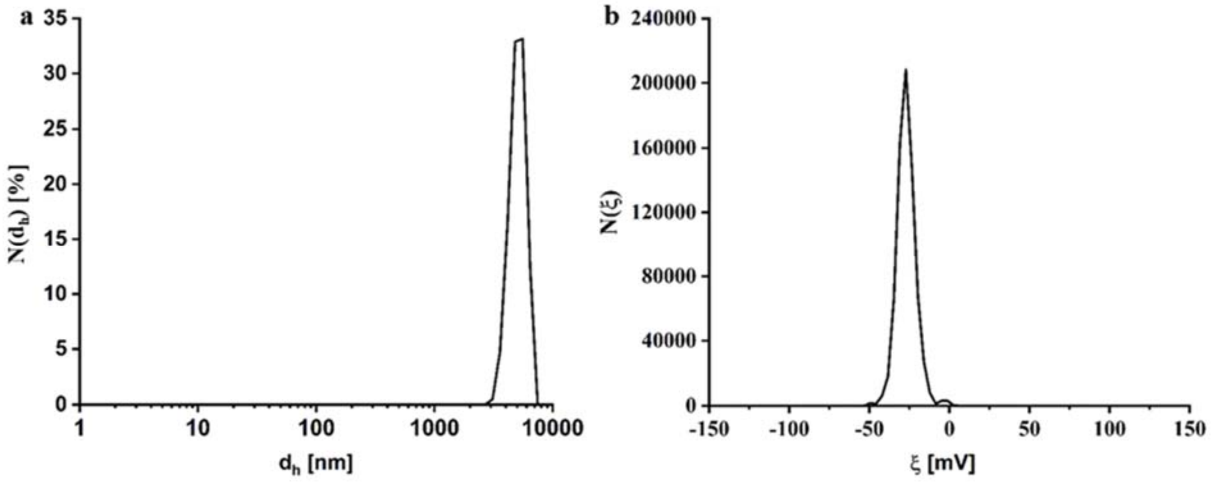

Figure SI.5 Colloidal characterizations of capsules with integrated star-shaped Au NPs. a) Number distribution $N(d_h)$ of the hydrodynamic diameter d_h as derived from dynamic light scattering (DLS) measurements in water. The mean hydrodynamic diameter was $d_h = 5.3 \pm 0.1$ µm. b) Zeta-potential distribution $N(\xi)$ measured in 40 mM Na_2HPO_4-NaH_2PO_4 buffer solution (pH 7.4). The mean zeta-potentials was $\xi = -26.4 \pm 1.2$ mV.

2. Cell models

2.1 Cell culture
2.2 Seeding geometries

2.1 Cell culture

Human cervical cancer cell (HeLa cell), breast cancer cell (MCF-7 cell), and mouse embryo fibroblast cell (NIH 3T3) were cultured in Dulbecco's Modified Eagles Medium (DMEM, #11965092, Thermo Fisher) with 4.5 g/L glucose supplemented with 10% fetal bovine serum (FBS, Biochrom, Germany, #S0615), 1% penicillin/streptomycin (P/S, #15070063, Thermo Fisher Scientific) at 5% CO_2 and 37 °C.

2.2 Seeding geometries

Three seeding geometries were used to study the cytosolic calcium increase and calcium spreading: i) Isolated single cells. There was no communication amongst adjacent cells, so that the response to external triggers only depended on individual cells; ii) Island model. No direct connection existed between cells, but chemical signaling via diffusion (e.g. ATP release) within cells was possible; iii) Interconnected cells. Adjacent cells were in direct contact and thus intercellular signaling (e.g. calcium and endogenous inositol 1,4,5-trisphosphate transport via gap junctions) was possible. The scheme of seeding geometries is shown in **Figure SI.6**.

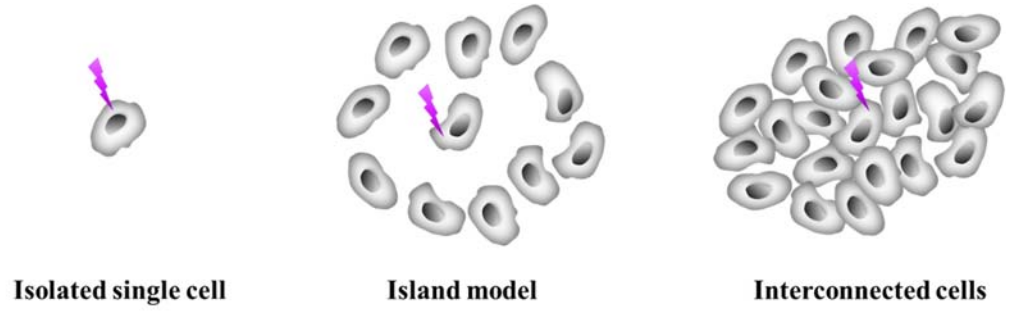

Figure SI.6 Seeding geometries of cells in this study.

The seeding densities for different geometries were as following. Note that the seeding conditions were different for the two different microscopes (widefield, laser scanning

microscope) used in this study. To obtain isolated single cells and island model cells, 15,000 MCF-7 cells or 7,500 HeLa cells per well in 300 μL of cell culture medium with 10% FBS were seeded into an 8-well μ-slide (#80826, I-bidi, 1.0 cm^2 seeding area per well) for widefield microscopy. 150,000 MCF-7 cells or 75,000 HeLa cells in 2 mL of cell culture medium with 10% FBS were seeded into a petri dish (#Z707651, Sigma Aldrich, 9.2 cm^2 seeding area) for laser scanning microscopy (LSM880, Zeiss). Cells were cultured for 24 h before use. To form interconnected cells, 30,000 MCF-7 cells or 15,000 HeLa cells were seeded for widefield microscopy. 300,000 MCF-7 cells or 150,000 HeLa cells were seeded for imaging with the LSM880. Cells were cultured for 48 h before use.

3. Calcium detection by Fluo-4

3.1 Response of Fluo-4 to calcium, pH, and temperature
3.2 Protocol for the detection of cytosolic calcium concentrations with Fluo-4
3.3 Measuring cytosolic calcium increase upon delivery of $CaCO_3$ containing capsules in an isolated single cell model
3.4 Measuring intercellular Ca^{2+} signaling upon delivery of $CaCO_3$ cores in interconnected cells

3.1 Response of Fluo-4 to calcium, pH, and temperature

The membrane-permeant dye Fluo-4 acetoxymethyl (AM) ester can be hydrolyzed to the calcium indicator Fluo-4 by endogenous esterases after diffusing into the cellular cytosol [5]. The fluorescence intensity of Fluo-4 is greatly enhanced upon binding to calcium with its carboxyl groups. One vial of Fluo-4 AM (#F14201, ThermoFisher, 50 µg per vial) was dissolved in 30.4 µL of anhydrous dimethyl sulfoxide (DMSO) (#D2650-5X5ML, Sigma Aldrich) to prepare 1.5 mM stock solution. Of note, according to the manufacturer's manuals, the stock solution of Fluo-4 in DMSO should be stored at -20 °C and used within a week to avoid decomposition and subsequent loss of cell loading capacity. To study the response of Fluo-4 to calcium, pH, and temperature, 15 µL of methanol and 20 µL of 0.5 M NaOH aqueous solution were added into 10 µL of Fluo-4 AM in DMSO, and the mixture was incubated at room temperature for 1 h in dark. Afterwards, 6 µL of hydrolyzed 0.33 mM Fluo-4 solution was diluted with 994 µL of 0.2 M Na_2HPO_4-NaH_2PO_4 (pH 7.5) to obtain 2 µM Fluo-4 in aqueous solution.

To measure the calcium response at pH 7.5 and 37 °C, 1 µL of $CaCl_2$ aqueous stock solution at different concentrations was added to 1 mL of 2 µM Fluo-4 solution to make calcium concentrations ranging from 0 to 6000 nM. To measure the temperature response at pH 7.5, 1 mL of 2 µM Fluo-4 aqueous solution containing 6000 nM calcium was incubated in a heating block (#8887001, ThermoFisher) at different temperatures for 5 min and measured immediately. To measure the pH response at 37 °C, 6 µL of hydrolyzed Fluo-4 (0.33 mM) solution was diluted with 994 µL of 0.2 M Na_2HPO_4-NaH_2PO_4 buffer at pH values ranging from 6.0 to 8.0 or with 994 µL of 0.2 M NaAc-HAc buffer at pH values ranging from 4.0 to 5.5. Buffer solutions were prepared according to the instruction in this web site

https://www.sigmaaldrich.com/life-science/core-bioreagents/biological-buffers/learning-center/buffer-reference-center.html. Afterwards, 1 μL of $CaCl_2$ stock solution was added to make a calcium concentration at 6000 nM.

The fluorescence of Fluo-4 was recorded by a fluorimeter. Fluo-4 was excited at 492 nm (slit: 5 nm) and its emission was collected from 500-700 nm (slit: 5nm). The response of Fluo-4 to calcium, pH, and temperature is shown in **Figure SI.7**.

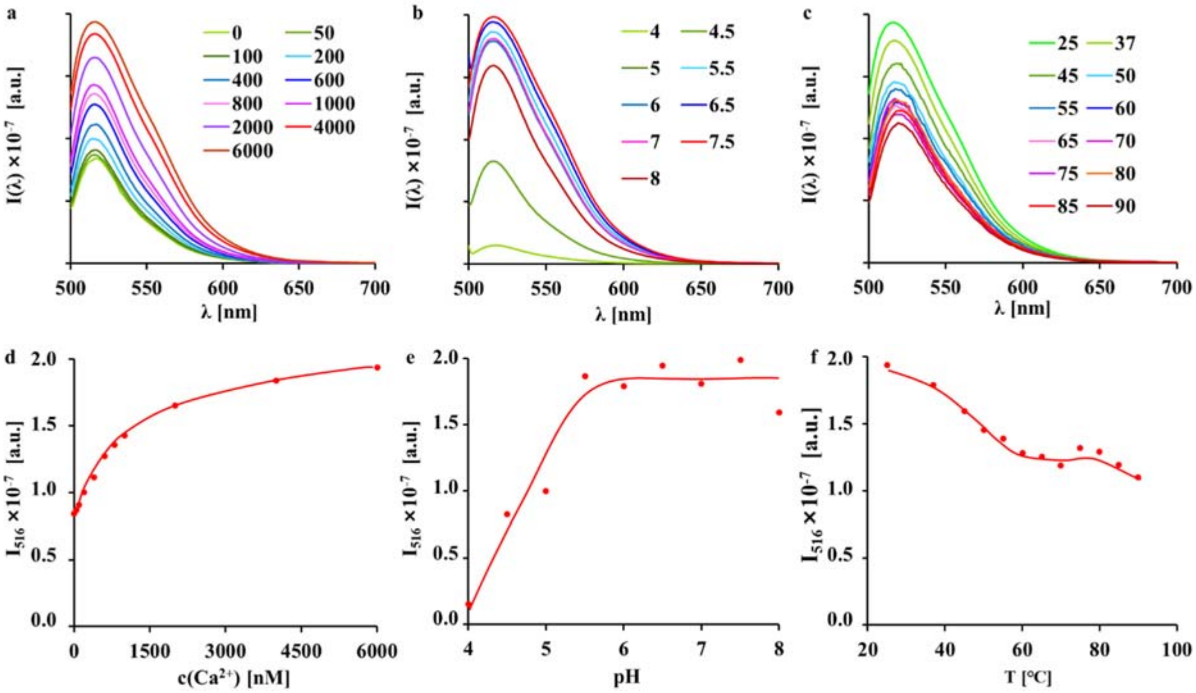

Figure SI.7 Response of 2 μM Fluo-4 to calcium, pH, and temperature. a) Fluorescence of Fluo-4 at calcium concentrations c(Ca^{2+}) ranging from 0 to 6000 ng/mL at pH 7.5 and at 37 °C. b) Fluorescence of Fluo-4 at pH values ranging from 4 to 8 at 37 °C at c(Ca^{2+}] = 6000 nM. c) Fluorescence of Fluo-4 at temperatures ranging from 25 °C to 90 °C at pH 7.5 and c(Ca^{2+}) = 6000 nM. d,e,f) Maximum fluorescence at emission wavelength λ_{em} = 516 nm of Fluo-4 at different d) calcium concentrations, e) pH values, and f) temperatures.

3.2 Protocol for detection of cytosolic calcium concentrations with Fluo-4

1.3 μL of 1.5 mM Fluo-4 in DMSO was diluted with 300 μL of phosphate buffered saline (PBS) to prepare the staining solution for widefield microscopy. 3.9 μL of 1.5 mM Fluo-4 in DMSO was diluted with 900 μL of PBS to prepare the staining solution for imaging with the laser-scanning microscope LSM880. Cells were washed with PBS for 3 times, and incubated with

staining solution for 30 min at room temperature to avoid undesired subcellular compartmentalization. Afterwards, cells were washed with PBS for 3 times by removing the medium and rinsing the cells in 1 mL of PBS. PBS was removed and cells were incubated with cell culture medium containing 10% FBS at 37°C for at least 15 min to allow complete de-esterification of acetoxymethyl (AM) ester of Fluo-4.

3.3 Measuring cytosolic calcium increase upon delivery of $CaCO_3$ containing capsules in an isolated single cell model

150,000 MCF-7 cells were seeded in a petri dish (seeding area 9.2 cm^2) in 2 mL of cell culture medium with 10% FBS and were incubated overnight. Afterwards, capsules containing SNARF-$CaCO_3$ cores were added at a density of 2 capsules/cell, and cells were immediately observed under the laser scanning microscope (here LSM510, not LSM880). Cells were imaged using appropriate laser excitation and detector selection (Fluo-4: λ_{ex} = 488 nm and λ_{em} = 505-540 nm; SNARF-yellow: λ_{ex} = 561 nm and λ_{em} = 565-590 nm; SNARF-red: λ_{ex} = 561 nm and λ_{em} = 600-700 nm). After internalization by cells via endocytosis [6], capsules containing undissolved SNARF-$CaCO_3$ cores were trafficked to lysosomes, where the acidic pH values degraded the cores and release calcium into the cytosol. The local pH values of the capsules were indicated by the red-to-yellow ratio (false colors) of the fluorescence signal I_r/I_y from the SNARF-$CaCO_3$ cores [6-7].

Illustration of image acquisition is shown in **Figure SI.8**. Cells were imaged every 4 min for 175 frames. To acquire the fluorescence of a capsule, 3 images at different lateral positions were acquired: one slice was obtained in the middle of the cell, while other two slices were obtained either 2.5 μm higher or lower. The fluorescence in the different slices was projected into one slice for further analysis. The data were analyzed by a custom-made MATLAB script. More details can be found in a previous report [6].

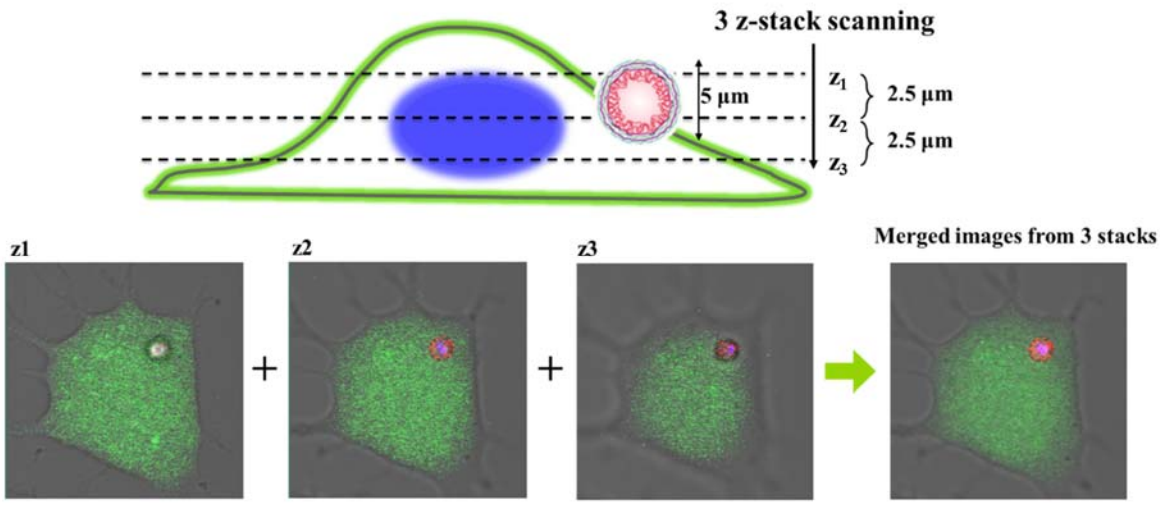

Figure SI.8 Illustration of image acquisition and fluorescence projection from 3 slices in z-axis direction. One slice was obtained in the middle of the cell, while other two slices were obtained either 2.5 µm higher or lower. The fluorescence from 3 slices was merged into one image.

The red-to-yellow ratio of the fluorescence signal I_r/I_y of SNARF and the cytosolic calcium intensity I_{Ca} indicated by Fluo-4 in an isolated single MCF-7 cell is shown in **Figure SI.9.**

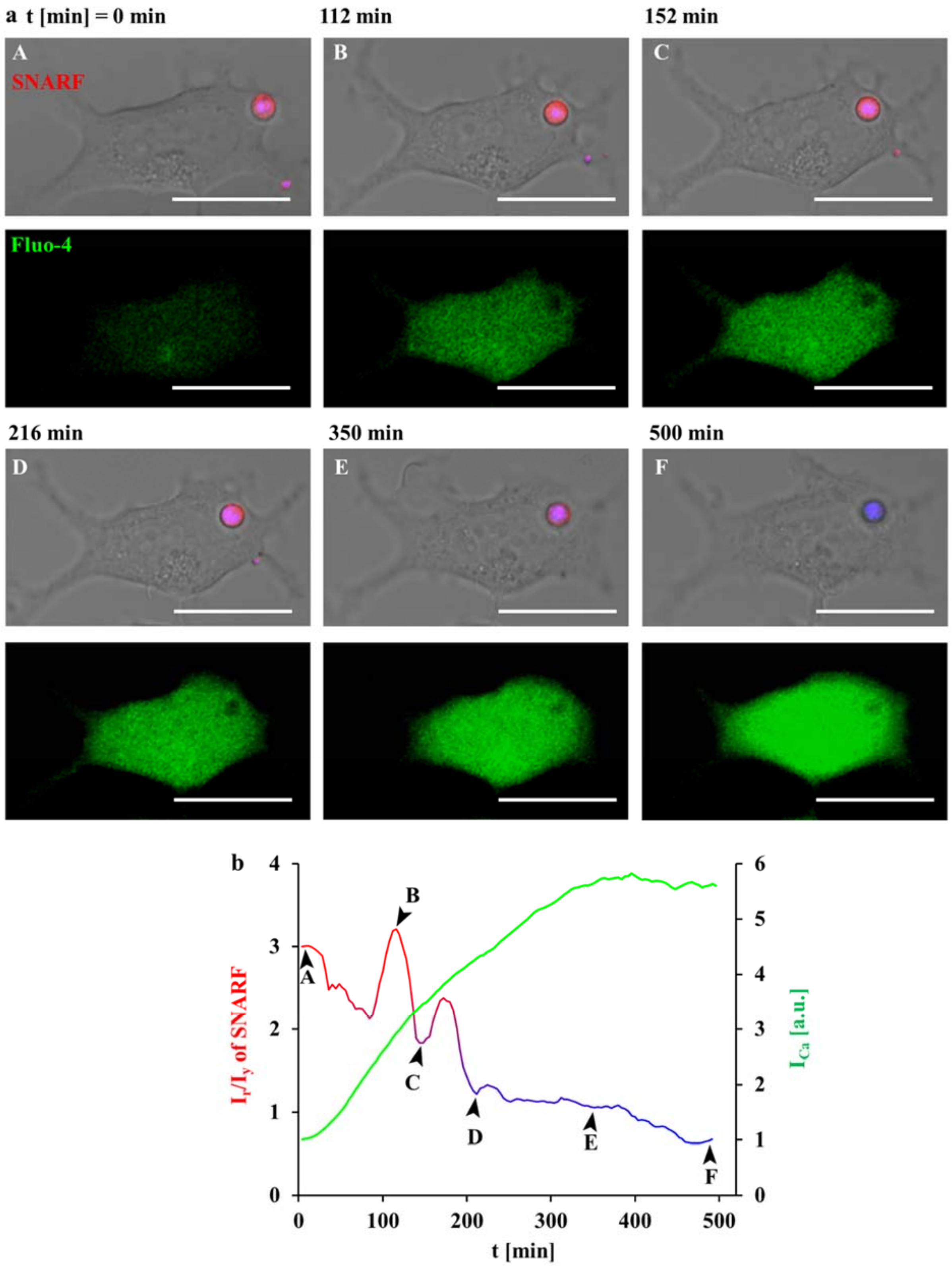

Figure SI.9 The red-to-yellow ratio of the SNARF fluorescence signal I_r/I_y, and the Fluo-4 signal of free cytosolic calcium I_{Ca} in an isolated single MCF-7 cell. The yellow fluorescence channel of SNARF is plotted in blue false colors. a) Representative time-lapse images A-F of capsules containing undissolved SNARF-$CaCO_3$ cores delivered into cells at different time points. Cytosolic calcium indicated by Fluo-4 as shown in green. Images were taken every 4

min. The scale bars represent 20 µm. b) The integrated fluorescence intensity of the calcium indicator Fluo-4 I_{Ca} over the cross section of the whole cell area was normalized to that when capsules were associated on the surface of the cell before endocytosis started (t = 0 min), which relates to the calcium concentration, plotted versus time t. The I_r/I_y ratios of SNARF signals are also plotted versus time t. The arrows indicated the time points corresponding to the A-F images shown in a).

As a control group, cells were stained with Fluo-4 but without capsules. Interestingly, the Fluo-4 fluorescence did not keep constant but decreased rapidly. This is caused by the leakage of Fluo-4 from cells, which is also observed for other calcium indicators in their free acid form [8]. The data are shown in **Figure SI.10**. Comparison between **Figure SI.9** and **Figure SI.10** proves that the increased cytosolic calcium was caused by the release of dissolved $CaCO_3$.

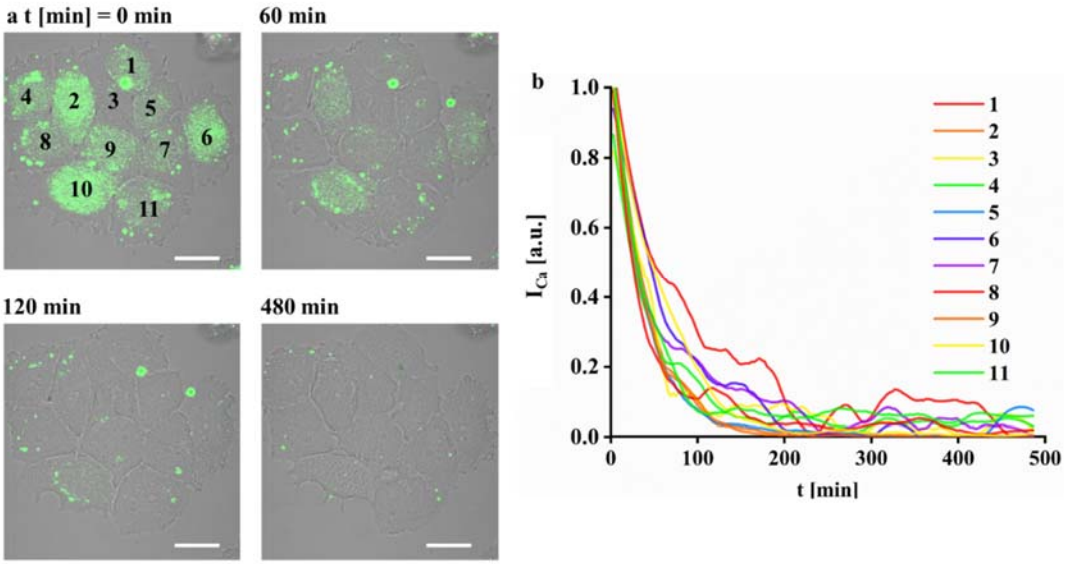

Figure SI.10. Cytosolic calcium increase in interconnected MCF-7 cells without added capsules containing undissolved $CaCO_3$. a) Representative images fo cells at different time points. Cells were imaged every 4 min. The scale bars represent 20 µm. b) The integrated fluorescence intensity of the calcium indicator Fluo-4 I_{Ca} over the cross section of the whole cells labelled with numbers was normalized to that in the beginning (t = 0 min). I_{Ca} which relates to the free calcium concentrations in these cells (plotted here versus time t) decreased here due to loss of Fluo-4 over time.

3.4 Measuring intercellular calcium signaling upon delivery of capsules containing Cy5-$CaCO_3$ cores in interconnected cells

To probe cell-cell communication, interconnected cells were studied. 350,000 MCF-7 cells were seeded per petri dish (9.2 cm^2 seeding area) in 2 mL of cell culture medium containing 10% FBS and were incubated overnight. Afterwards, capsules containing undissolved Cy5-$CaCO_3$ cores were added at a density of 1 capsule/cell, and cells were immediately observed under the laser scanning microscope. Cells were imaged using appropriate laser excitation and detector settings (Fluo-4: λ_{ex} = 488 nm and λ_{em} = 500-550 nm; Cy5: λ_{ex} = 633 nm and λ_{em} = 660-750 nm). Other settings were the same as mentioned above. The data are shown in **Figure SI.11**.

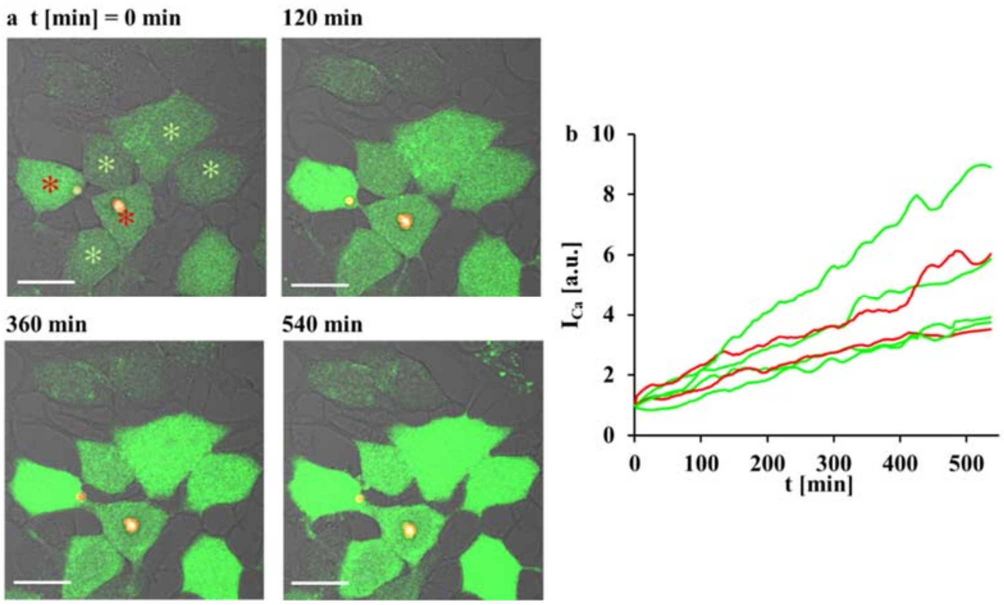

Figure SI.11. Cytosolic calcium increase in interconnected MCF-7 cells. a) Representative images of capsules delivered into cells at different time points. Capsules containing undissolved Cy5-$CaCO_3$ cores (shown in red) were delivered into two cells labelled with red stars, as can be seen by the red Cy5 fluorescence. Cells interconnected with those cells which had internalized capsules are labelled with green stars. Images were taken every 4 min. The scale bars represent 20 µm. b) The integrated fluorescence intensity of the calcium indicator Fluo-4 I_{Ca} over the cross section of the whole cells labelled with stars was normalized to that in the beginning (t = 0 min). I_{Ca} relates to the calcium concentrations in these cells and is plotted versus time t. This image shows that the calcium excitation is passed to the connected cells, but not to other cells.

4. Photothermal heating of capsules

4.1 Microscopy set-ups

4.2 Photothermal heating of internalized capsules

4.3 Probing for damage to cells upon photothermal heating

4.1 Microscopy set-up

Two microscopy set-ups, namely a widefield microscope and a confocal laser scanning microscope (LSM880), were utilized for the photothermal heating of capsules, and the subsequent real time imaging in this study. We first used an inverted widefield microscope, which have been used in several previous publications in our lab [2, 3b, 9]. Later-on a confocal laser scanning microscope came available and experiments were repeated. As there were some differences in the results here in the Supporting Information data as obtained with the 2 different set-up are reported.

Widefield set-up: Concerning the widefield microscope a schematic diagram of the optical set-up and the corresponding light paths in widefield microscope is shown in **Figure SI.12.** A continuous wave laser diode with 830 nm wavelength and with maximum power up to 100 mW generator (Rapp OptoElectronic, Germany) was coupled with an Axiovert 200 M widefield microscope (Zeiss). The transmission illumination together with the laser irradiation spot (from the Rapp OptoElectronic set-up) and fluorescent images were obtained separately from different light paths. As shown in **Figure SI.12**, the transmission light from the top was provided by a 100 W halogen lamp (HAL-100, Zeiss). The laser from the DL-830 laser generator (Rapp OptoElectronic, Germany) was reflected by a short-pass beamsplitter (2P-Beamsplitter 725 DCSPXR, AHF Analysentechnik AG) and focused onto the sample through an oil-immersed objective (Apochromat 63×/1.40 Oil DIC or Apochromat 100×/1.46 Oil DIC). The fluorescence illumination was provided by a HBO-100 lamp (Zeiss). In this case, the fluorescent filter CHROMA 31001 (excitation: 480/30 nm; beamsplitter: long pass 505 nm; emission: 535/40 nm; CHROMA) was used to detect the Fluo-4 signals. The fluorescent filter AHF Cy5 (excitation: 620/60 nm; beamsplitter: long pass 660 nm; emission: 700/75 nm; AHF Analysentechnik) was used to detect the CellTracker Deep Red signals. Images were collected by a CCD camera by the software AxioVision (release 4.6 from Zeiss). To acquire images with both high quality and temporal resolution, the time interval between adjacent images was set

that one image was taken every 4 seconds when 2 channels (transmission and Fluo-4) were used, or every 8 seconds when 3 channels (transmission, Fluo-4 and Celltracker Deep Red) were used.

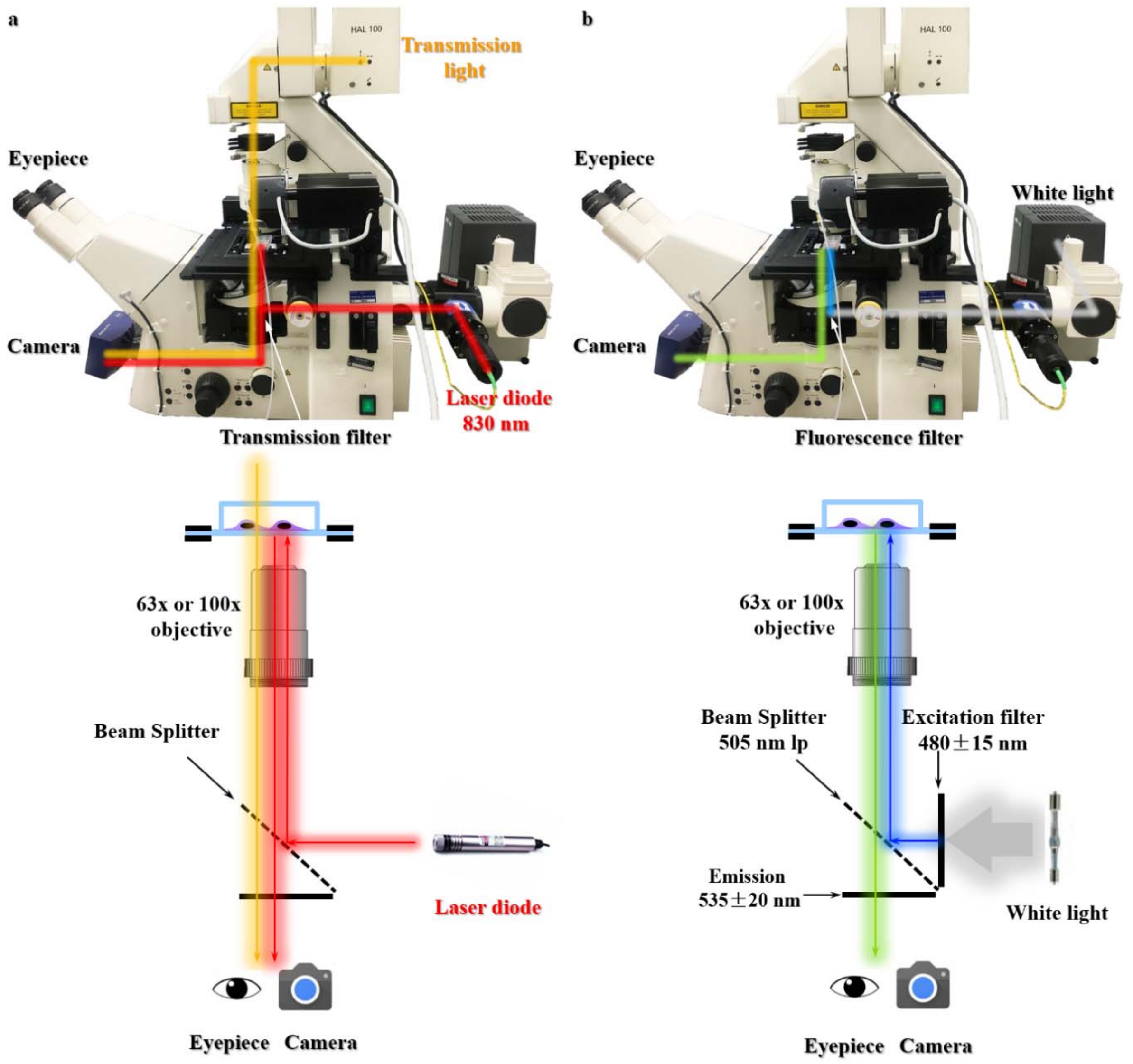

Figure SI.12. Optical set-up and the corresponding light paths of a) transmission and laser spot, and b) excitation and emission.

When fluorescence signals in MCF-7 cells were excited by the widefield microscope, the whole specimen volume was irradiated by the blue light (480/30 nm). Here, the output laser power from the excitation laser was measured at 480 nm by a powermeter (**Figure SI.13**). For each frame, the excitation time was ca. Δt_{laser} = 1 s. To minimize photobleaching and photo-induced cytotoxicity, 33% of the maximum laser power (P_{laser} = 163 μW; E_{laser} = 163 μJ) and 55% of

maximum laser power (P_{laser} = 154 µW, E_{laser} = 154 µJ) were used for excitation under the 63× and 100× objectives, respectively. However, later we found that the excitation laser power can trigger cytosolic Ca^{2+} increase during imaging. This artefact will be discussed in the later section. The high excitation laser power can also cause photobleching of Fluo-4 signals. In contrast, these artifacts were not observed with the laser scanning microscope set-up.

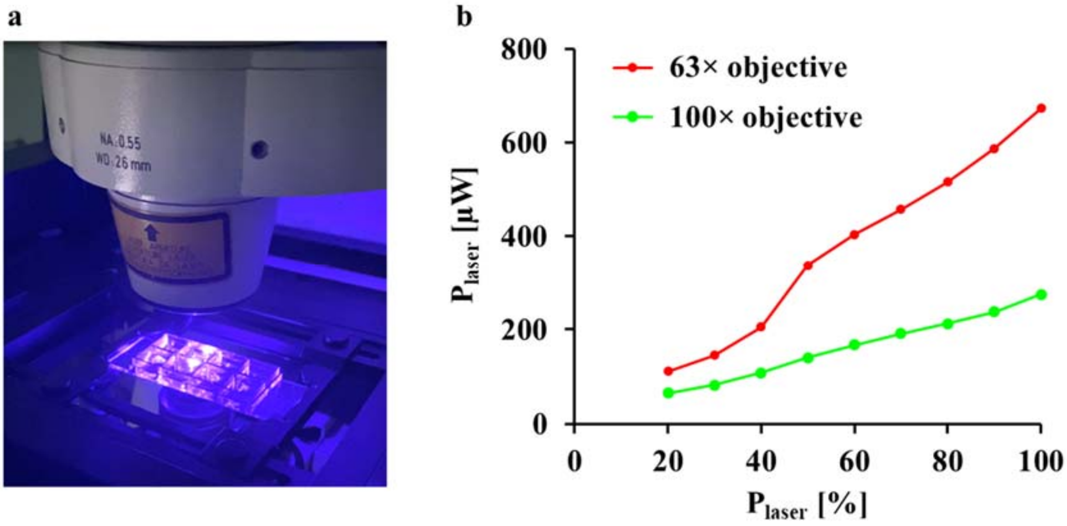

Figure SI.13 a) Representative image of the laser excitation (480/30 nm) through a 63× objective in the widefield microscope. An 8-well µ-slide was illuminated in this case. B) The output laser power measured at 480 nm through 63× and 100 × objectives. P_{laser} [%] is the percentage of maximum laser power from the excitation laser. P_{laser} [µW] is the laser power measured at 480 nm with a powermeter at the focus of the laser.

The laser spots (830 nm) used for photothermal heating at 10% of laser power through the 63× and 100× objectives can be observed directly in the center of the screen, as shown in **Figure SI.14a**. Before use, the laser was calibrated and focused to the focal plane of the samples. The outer rings of the laser spot are caused by diffraction. The areas of the focused spot for the 63× and 100× objectives were A_{laser} = 2.34 and 1.17 µm^2, respectively, as measured by Image J. The output laser power through both objectives was measured by a powermeter (**Figure SI.14b**). The irradiation time was manually controlled to ca. Δt_{laser} = 2 s. Therefore, 33% (P_{laser} = 698 µW, I_{laser} = 2.98 × 10^8 W/m^2; E_{laser} = 1.40 mJ, U_{laser} = 5.96 × 10^8 J/m^2) and 90% (P_{laser} = 622 µW, I_{laser} = 5.32 × 10^8 W/m^2; E_{laser} = 1.24 mJ, U_{laser} = 1.06 × 10^9 J/m^2) of the maximum laser power through the 63× and 100× objectives were used for photothermal heating.

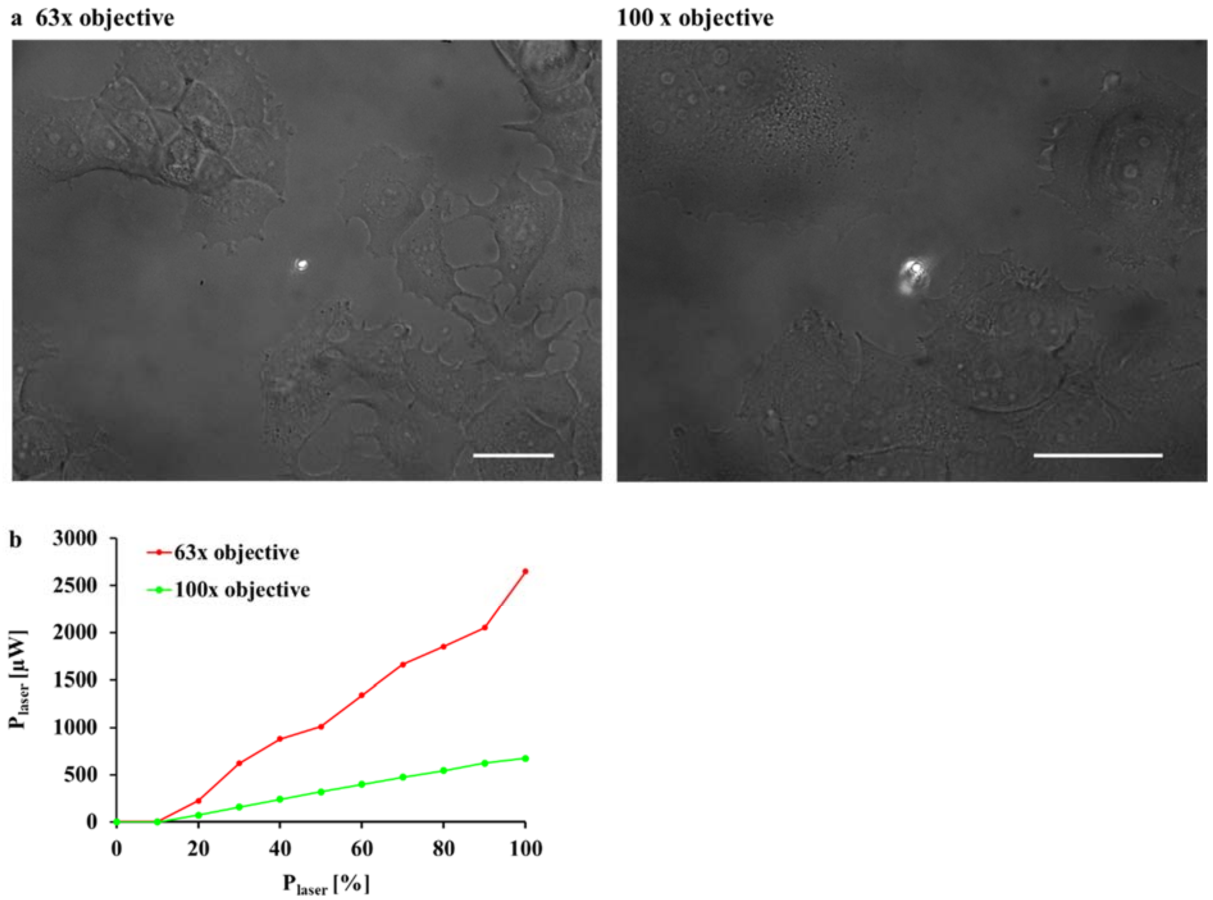

Figure SI.14. a) Laser spots as seen through the 63× and 100× objectives in the widefield microscope. The scale bar corresponds to 20 µm. b) Corresponding laser powers at 830 nm. P_{laser} [%] is the percentage of maximum laser power from the excitation laser. P_{laser} [µW] is the laser power measured at 830 nm with a powermeter at the focus of the laser.

<u>Laser scanning microscopy set-up:</u> Due to the artefact of possible auto-excitation caused by the widefield microscope, we later used a newly purchased LSM880 confocal microscope. The set-up of the LSM880 is shown in **Figure SI.15**. A continuous wave laser ranging from 690-1040 nm was generated from MaiTai wide band, mode-locked Ti:Sapphire laser (Spectra Physics). Both, the excitation laser (488 nm for imaging) and the irradiation laser (830 nm for photothermal heating) were focused through a water immersed objective (W Plan-Apochromat 20×/1.0 DIC D = 0.17 (UV) VIS-IR M27 75mm). As this system is an upright microscope, the objective is immersed in the cell culture medium during measurements. The temperature in the black box (incubator) was kept constantly at 25 °C.

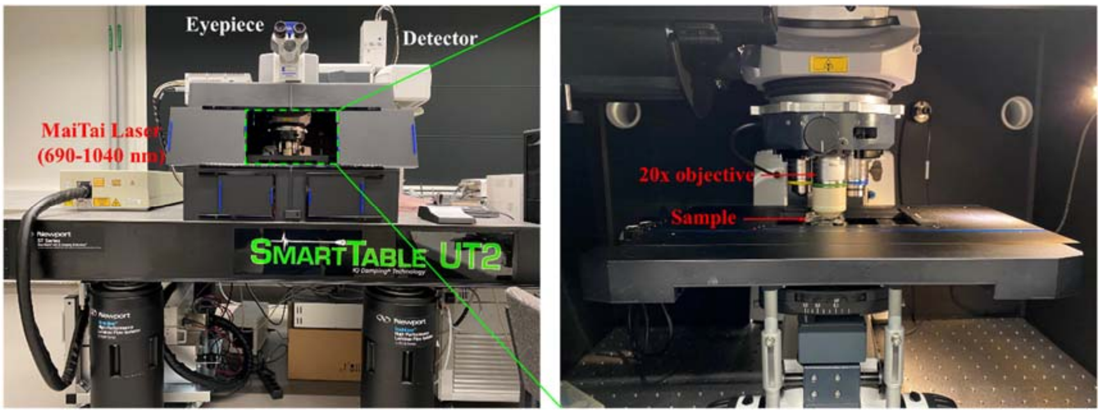

Figure SI.15. Set-up of the LSM880.

When Fluo-4 with or without Celltracker Deep Red was used, the parameters were set as follows: Fluo-4 was excited at 488 nm (with an argon-laser) at 0.6% of maximum laser power, and the emission from 490-600 nm was collected by a GaAsp PMT spectral detector. Celltracker Deep Red was excited at 633 nm (with a HeNe-laser) at 0.8% of maximum laser power, and the emission from 650-700 nm was collected. Because there is no overlap in their emission spectra, images can be excited by two lasers simultaneously. To acquire images with both high quality and temporal resolution, in most cases the resolution of images was set as 512 × 512 pixels, the zoom value was set at 2, and the scanning time for each image was 1.26 s. The time interval between adjacent images was 2 s.

When Fluo-4 and SNARF-1 were used, the parameters were set as follows: Fluo-4 was excited at 488 nm (with an argon-laser) at 0.6% of maximum laser power, and the emission from 490-556 nm ("~~blue~~ green" channel, false color) was collected by a GaAsp PMT spectral detector. SNARF-1 was excited at 561 nm (with a HeNe-laser) at 0.6% of maximum laser power. Its fluorescence was recorded between 570-606 nm ("~~green~~ blue" channel) and 625-750 nm ("red" channel). Due to the overlap in their emission spectra, Fluo-4 and SNARF-1 should be excited separately. To acquire images with both high quality and temporal resolution, the resolution of images was set as 400 × 400 pixels. The zoon was set as large as possible, and the scanning time for each image was 1.97 s. The time interval between adjacent images was 2 s.

When tumor spheroids were imaged, the resolution of images was set as 400 × 400 pixels, the zoom value was set at 3, and the scanning time for each image was 3.85 s. The time interval between adjacent images was 4 s.

Different from the widefield microscope, the laser is focused through a pinhole and subsequently into the sample, leading to a relatively small excitation volume. The output laser power from the excitation laser 488 nm was measured by a powermeter (**Figure SI.16**). The laser power and energy at 0.6% of the maximum laser power is P_{laser} = 15.4 μW and E_{laser} = 19.4 μJ.

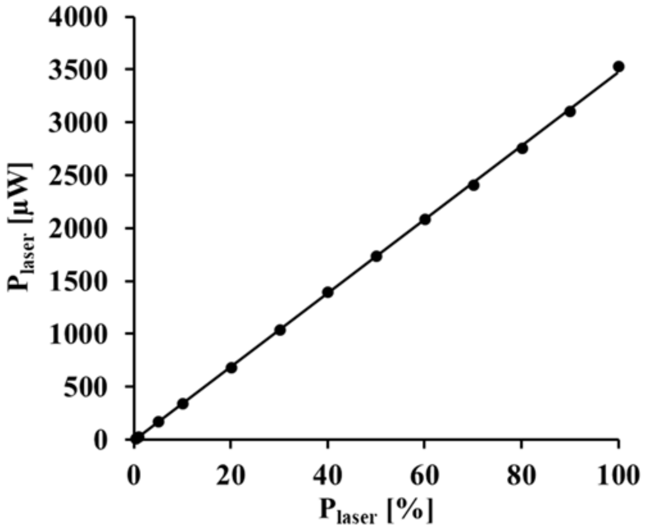

Figure SI.16. Output laser power (488 nm) through a 20× objective. P_{laser} [%] is the percentage of maximum laser power from the excitation laser. P_{laser} [μW] is the laser power measured at 488 nm with a powermeter at the focus of the laser.

For photothermal heating, the “photoblech” and “region” modules in the ZEN 2.3 SP1 software were used to irradiate capsules within a custom made region. Different from the widefield microscope, the laser spot cannot be observed directly from the screen, and only the area in the defined region was irradiated. The maximum laser power at 830 nm was ca. 2850 mW, which can be directly read from the software. In most cases, the irradiation time was ca. Δt_{laser} = 0.039 s and irradiation area was A_{laser} = 12.56 μm^2. 1.2% (P_{laser} = 34.2 mW, I_{laser} = 2.72 × 10^9 W/m^2; E_{laser} = 1.33 mJ, U_{laser} = 1.06 × 10^8 J/m^2) of laser power was used. In the case of MCF-7 cells with interconnected and coculture models, higher laser power up to 2.5% (P_{laser} = 71.25 mW, I_{laser} = 5.67 × 10^9 W/m^2; E_{laser} = 2.78 mJ, U_{laser} = 2.21× 10^8 J/m^2). A comparison between the widefield microscope and the laser scanning LSM 880 microscope is shown in **Table S1**.

		**Widefield microscope**		**LSM880**
**Objective**		63×	100×	20×
**Excitation**	λ_{ex} [nm]	480/30	480/30	488

	Δt_{laser} [s]	1	1	1.26	
	P_{laser} [μW]	163	154	15.4	
	E_{laser} [μJ] = $P_{laser} \cdot \Delta t_{laser}$	163	154	19.4	
**Irradiation**	Δt_{laser} [s]	2	2	0.039	
	A_{laser} [μm^2]	2.34	1.17	12.56	
	P_{laser} [%]	33	90	1.2	2.5
	P_{laser} [mW]	0.698	0.622	34.2	71.25
	I_{laser} [W/m^2]	2.98×10^8	5.32×10^8	2.72×10^9	5.67×10^9
	E_{laser} [mJ] = $P_{laser} \cdot \Delta t_{laser}$	1.4	1.24	1.33	2.78
	U_{laser} [J/m^2]= E_{laser}/A_{laser}	5.96×10^8	1.06×10^9	1.06×10^8	2.21×10^8

Table S1. Comparison between widefield microscope and LSM880. Data show that the deposited energy upon irradiation E_{laser} is very similar for both set-ups. In the case of laser scanning microscope shorter pulses of higher laser power were used.

4.2 Photothermal heating of internalized capsules

Widefield microscopy set-up: First, it was demonstrated that intracellular Ca^{2+} is released by the local irradiation of individual capsules. Irradiation not hitting a capsule did not cause the release of intracellular Ca^{2+}.

15,000 MCF-7 cells were seeded in each well of the 8-well μ-slide in 300 μL of cell culture medium containing 10% FBS and incubated overnight. This density resulted in isolated cells without contact to adjacent cells (isolated cell model). Afterwards, hollow capsules with embedded star-shapd Au NPs were added at a density of 2 capsules/cell, and cells were incubated overnight to ensure capsule internalization.

First, the laser spot was moved to a position ca. 5 μm far away from a capsule, and cells were imaged after 4 s. While in this way the capsule was not directly irradiated, it could be still possible that diffraction of the laser could heat the capsule. However, as shown in **Figure SI.17** no additionally released intracellular Ca^{2+} (as imaged by Fluo-4) could be observed. In contrast, when after 5 min the laser was positioned to directly irradiate the same capsule in the same cell,

and cells were imaged after 4 s, release of intracellular Ca^{2+} could be observed by increase in Fluo-4 fluorescence. Two examples are shown in **Figure SI.17.** Thus, while there is diffraction of the laser spot intracellular Ca^{2+} is only released when capsules are directly irradiated.

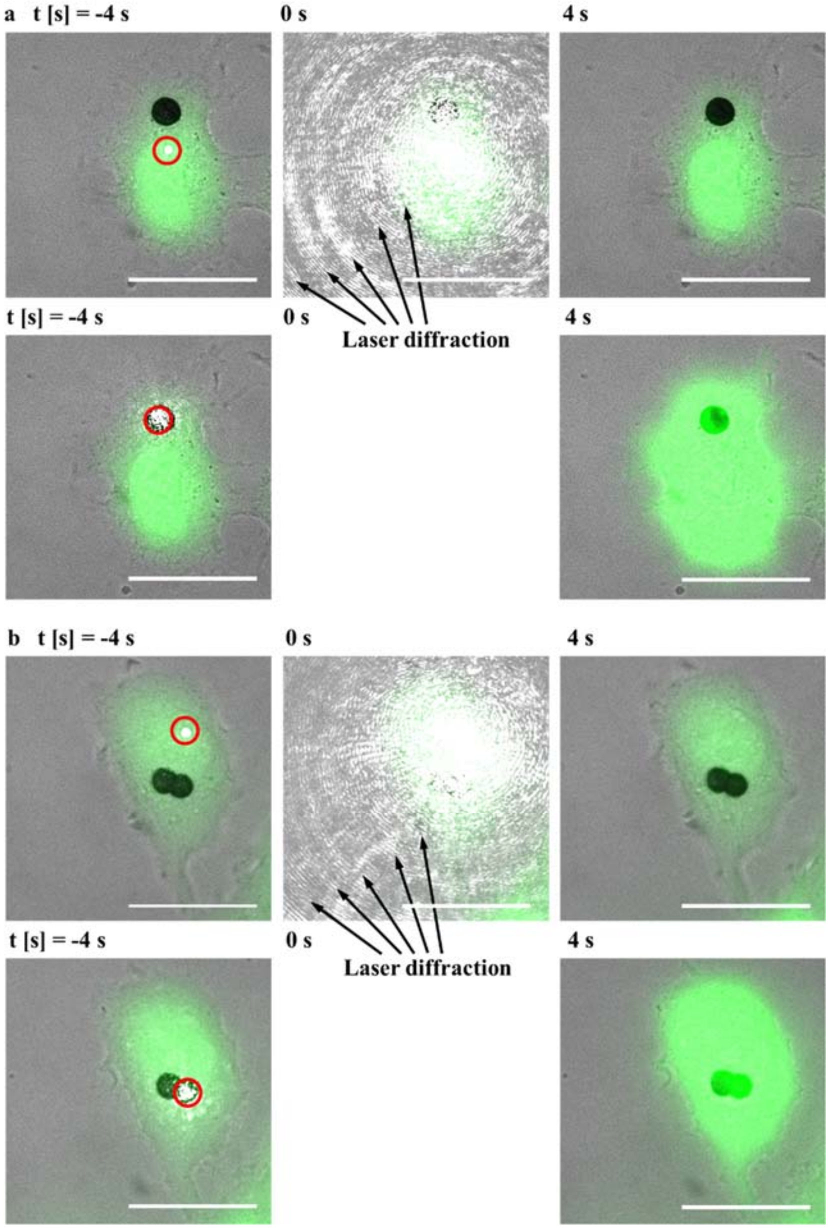

Figure SI.17. Capsules with embedded star-shaped Au NPs were endocytosed by MCF-7 cells, which were loaded by Fluo-4. Top line: At time t = 0 s, a position ca. 5 µm far away from the capsule as indicated by the red circle was excited with an 830 nm laser spot of ca. 1.17 μm^2 (100× objective, widefield microscope). Bottom line: afterwards, the same capsule as indicated by the red circle was directly irradiated at P_{laser} = 0.622 mW (at the illumination spot) for Δt_{laser} = 2 s. Two examples (a and b) are demonstrated. The scar bars represent 20 µm.

However, we later realized that in some cases the excitation light (480/30 nm) for imaging was so strong that cytosolic calcium increase was evoked without laser irradiation at 830 nm. One example is shown in **Figure SI.18**. This is consistent with other findings which reported 2-3 folds increase of resting intracellular fluorescence of Fluo-4 in HEK293 cells and HaCaT keratinocytes after applying a 488 nm laser at ca. 10 µW laser power (0.18 $\mu W/\mu m^2$) at 1 Hz [10]. Therefore, all the data were repeated with the newly purchased LSM880. However, also data obtained from the widefield microscope are reported here in the Supporting Information, in order to allow to directly compare the data from both microscopy set-ups.

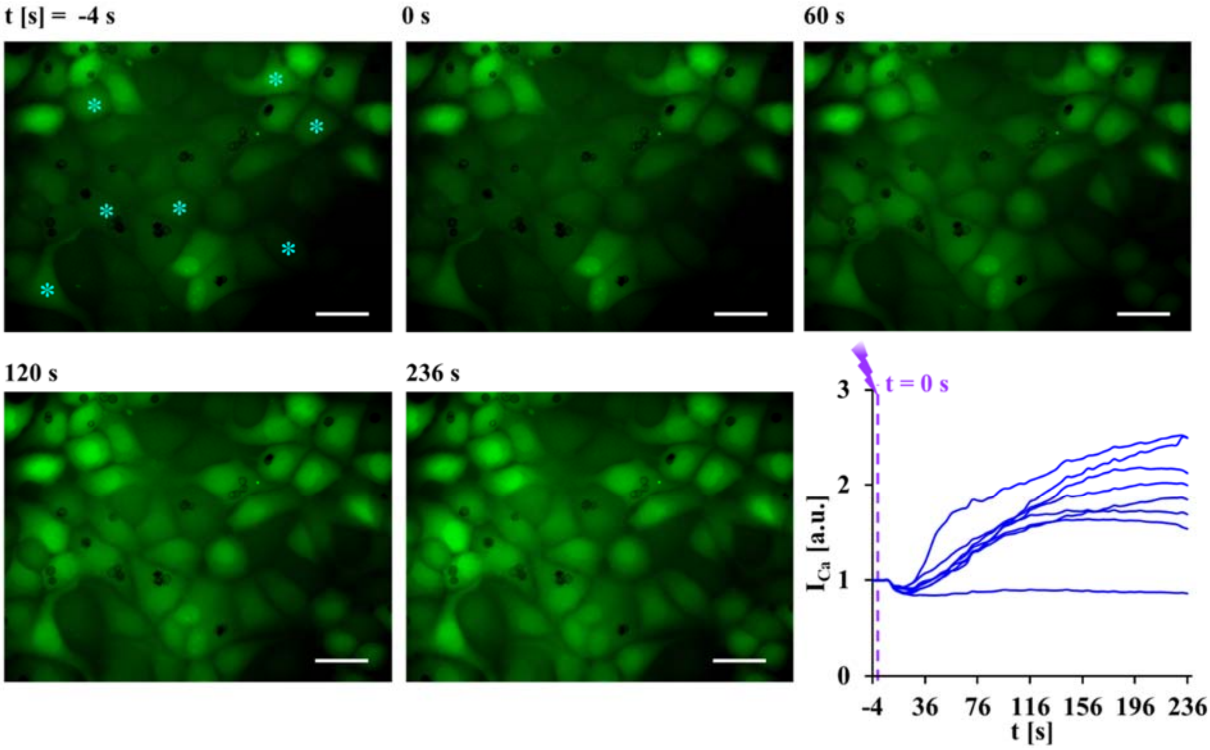

Figure SI.18. MCF-7 cells has endocytosed capsules with integrated star-shaped NPs and have been loaded with Fluo-4. MCF-7 cells were repeatedly imaged at λ_{ex} = 480/30 nm at P_{laser} = 163 µW for Δt_{laser} = 1 s (63× objective, widefield microscope) every 4 s for 60 times. The integrated fluorescence intensity of the calcium indicator Fluo-4 I_{Ca} over the cross section of the whole cell area was normalized to that before irradiation (t = - 4 s), which relates to the Ca^{2+} concentration, and is plotted versus time t. Data demonstrate that the excitation light (at 480/30 nm) during imaging with the widefield microscope can evoke cytosolic calcium release (i.e. raise in the Fluo-4 fluorescence), without having irradiated the capsules at 830 nm. The scar bars represent 20 µm.

Laser-scanning microscopy set-up (LSM880): To test if the light irradiation (830 nm) itself can cause cytosolic calcium increase (e.g. without photothermal heating of a capsule), an isolated single MCF-7 without capsules (control group) was irradiated. 150,000 MCF-7 cells were seeded in a petri dish in 2 mL of cell culture medium containing 10% FBS and were incubated overnight. Afterwards, a single MCF-7 cell was irradiated at P_{laser} = 142.5 mW for Δt_{laser} = 260 ms. Thus, the total irradiation energy E_{laser} = 37.1 mJ. Cells were imaged every 2 s for 60 times. The two examples in **Figure SI.19** show that after laser irradiation at 830 nm, the cytosolic calcium as monitored by the Fluo-4 fluorescence remained unchanged**.** This result proves that the excitation light in the LSM880 set-up can not trigger cytosolic calcium increase, if no capsule is present and irradiated. Thus the artefact observed in the widefield microscope (**Figure SI.17**) is avoided.

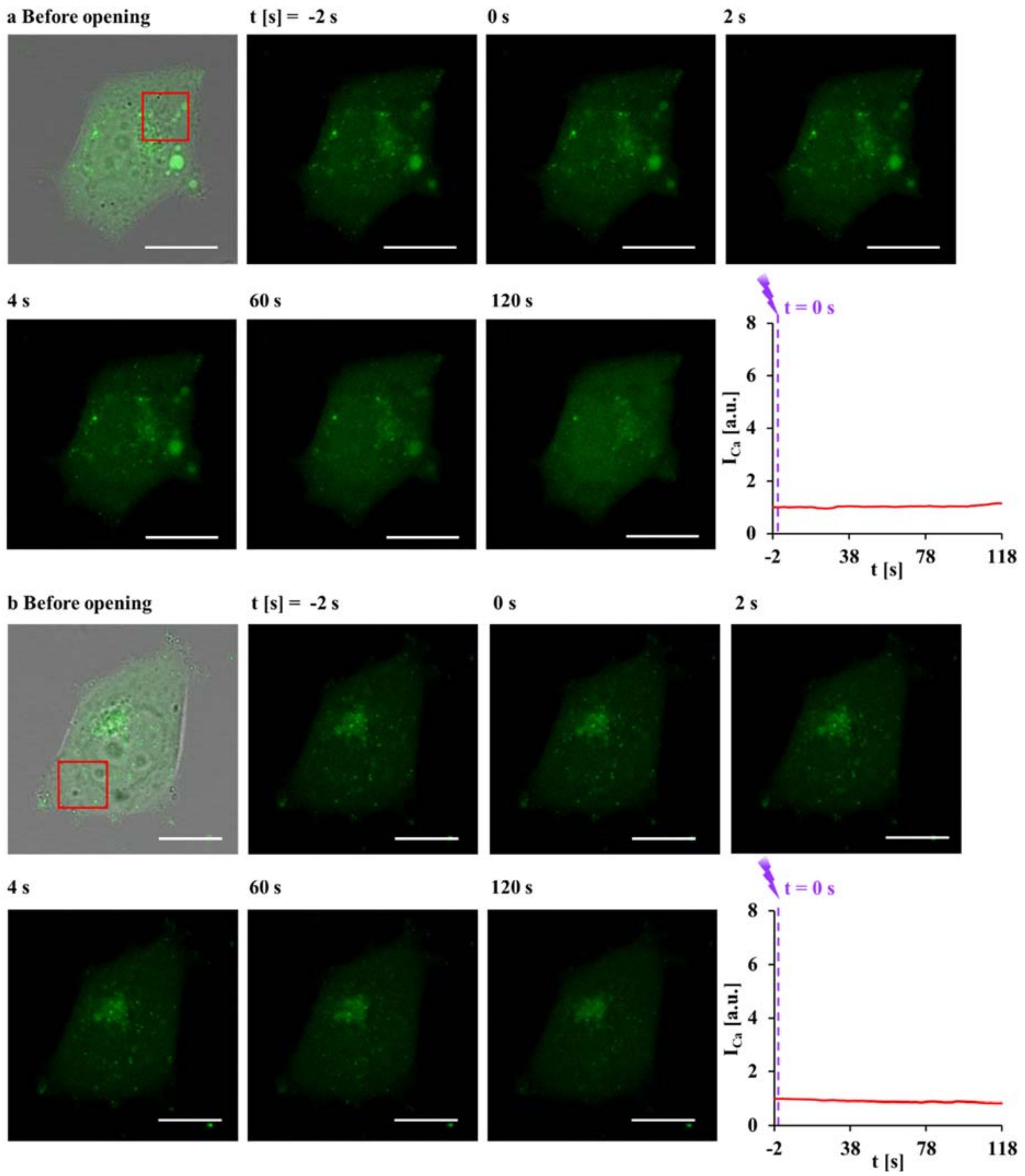

Figure SI.19 At time t = 0 s one single cell without internalizing capsules was irradiated with the 830 nm laser at P_{laser} = 142.5 mW for an average time of Δt_{laser} = 260 ms. The irradiation regions are indicated by the red box. Two examples are shown here. Images were taken every 2 s. The integrated fluorescence intensity of the calcium indicator Fluo-4 I_{Ca} over the cross section of the whole cell area was normalized to that before irradiation (t = - 2 s), which relates to the calcium concentration is plotted versus time t. No increase in Fluo-4 fluorescence is observed upon irradiation at 830 nm.

4.3 Probing for damage to cells upon photothermal heating

Photothermal heating at high laser powers may result in hyperthermia-induced cell necrosis [11]. There is a range of laser power which is sufficient to rupture the walls of the capsules and surrounding endo/lysosomal membrane, while keeping cells alive [12]. Herein, the laser irradiation in widefield microscope was evaluated with the isolated cell model to check if under conditions where photothermal heating can trigger intracellular calcium release there is no reduction in cell viability, i.e. no cell necrosis. Of note, heat originating from the capsules depends on the concentration of the star-shaped Au NPs in the capsules' walls. Inhomogeneous Au NP distributions in the capsules could result in different cell viability even under the same laser excitation power, as in capsules with local high concentrations of Au NPs (i.e. local agglomerates of Au NPs in the capsule walls) lead to higher temperatures upon photothermal heating [12].

In order to probe cell viability upon photothermal heating a LIVE/DEAD Viability/Cytotoxicity Kit (#L3224, Thermo Fisher Scientific) was used [12]. 35,000 MCF-7 cells were seeded in each well of a 8-well μ-slide with grid (#80826-G500, Ibidi) in 300 μL of cell culture medium containing 10% FBS and were incubated overnight. Afterwards, capsules with embedded star-shaped Au NPs were added at a density of 2 capsules/cell and cells were incubated overnight to ensure capsule internalization via endocytosis. As shown in **Figure SI.20**, capsules inside cells at the corner of the grid (e.g. D4, top left corner) were excited with an 830 nm laser spot of ca. A_{laser} = 2.34 μm^2 (63× objective, widefield microscope) at 0.698 mW (at the illumination spot) for Δt_{laser} = 2 s. The irradiated cells were imaged and marked with red circles. The positions of the irradiated cells on the grid were recorded so that cells in the same position could be found later again. After 2 h, the cells were stained with 300 μL of PBS containing 0.2 μL of 4 mM calcein AM in DMSO, and 0.6 μL of 2 mM EthD-1 in DMSO/H_2O=1/1 (v/v) at 37°C for 30 min from the LIVE/DEAD Viability/Cytotoxicity Kit. Live (stained with calcein shown in green) and dead cells (stained with EthD-1 cells shown in red or missing cells) were counted. For each experiment, cells from at least 2 grids (8 corners) were measured. The experiment was repeated for 3 times. More than 120 cells from 3 independent experiments were calculated. The images and statistic data are shown in **Figure SI.21.** Data indicate that at the used laser powers for photothermal heating for initiating release of intracellular Ca^{2+} there was only little reduction in cell viability. We also conducted similar experiment in LSM880 in a recent

publication [3a] and for the laser scanning microscopy set-up there was some reduction in cell viability upon the here used laser powers for photothermal heating.

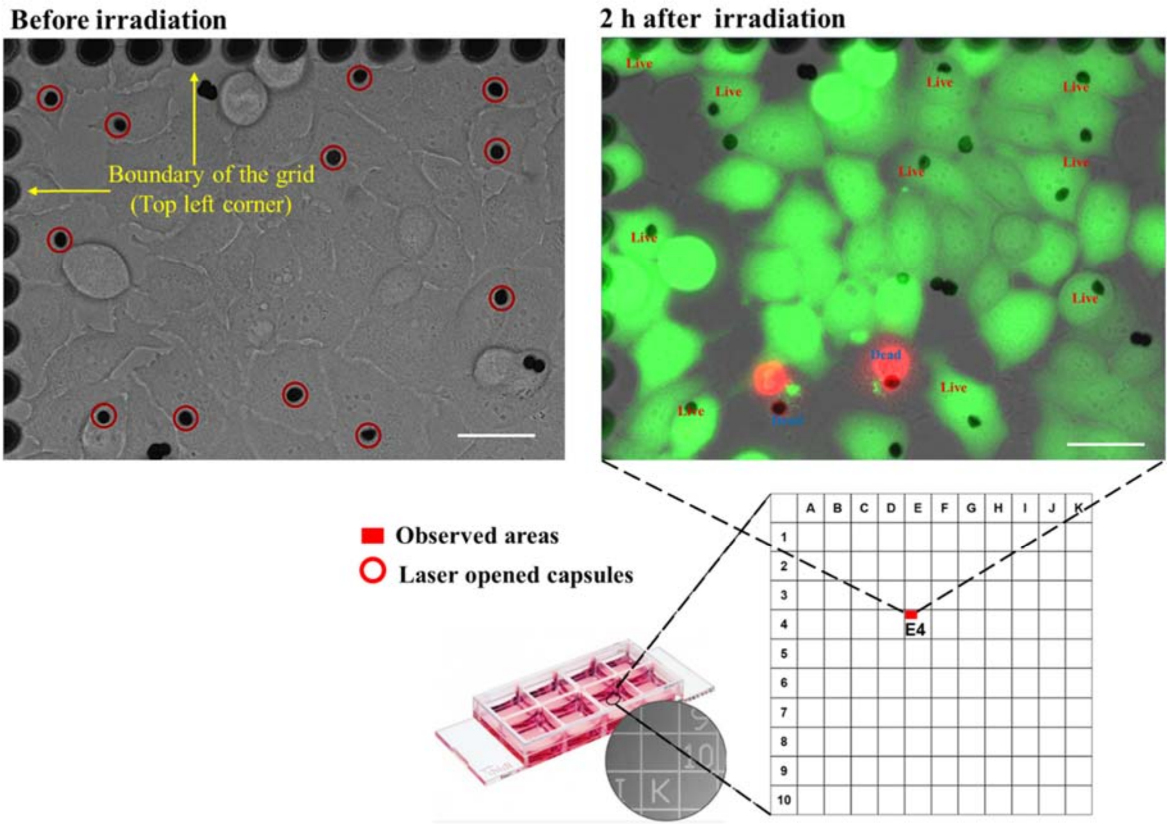

Figure SI.20. Scheme for measuring cell viability after laser irradiation. An 8-well µ-slide with grid was used. Cells with endocytosed capsules with embedded star-shaped Au NPs (the capsules can be seen as dark spots in the cells) were excited with an 830 nm laser spot of ca. 2.34 μm^2 (63× objective, widefield micrscope) at 0.698 mW (at the illumination spot) for Δt_{laser} = 2 s. The irradiation regions are indicated by the red circles. The cell viability was determined by live/dead staining after 2 h irradiation. For this the cells in the same position were observed. Live cells filled with calcein fluoresce in green. Dead cells stained with EthD-1 fluoresce in red. The scale bars represent 20 µm.

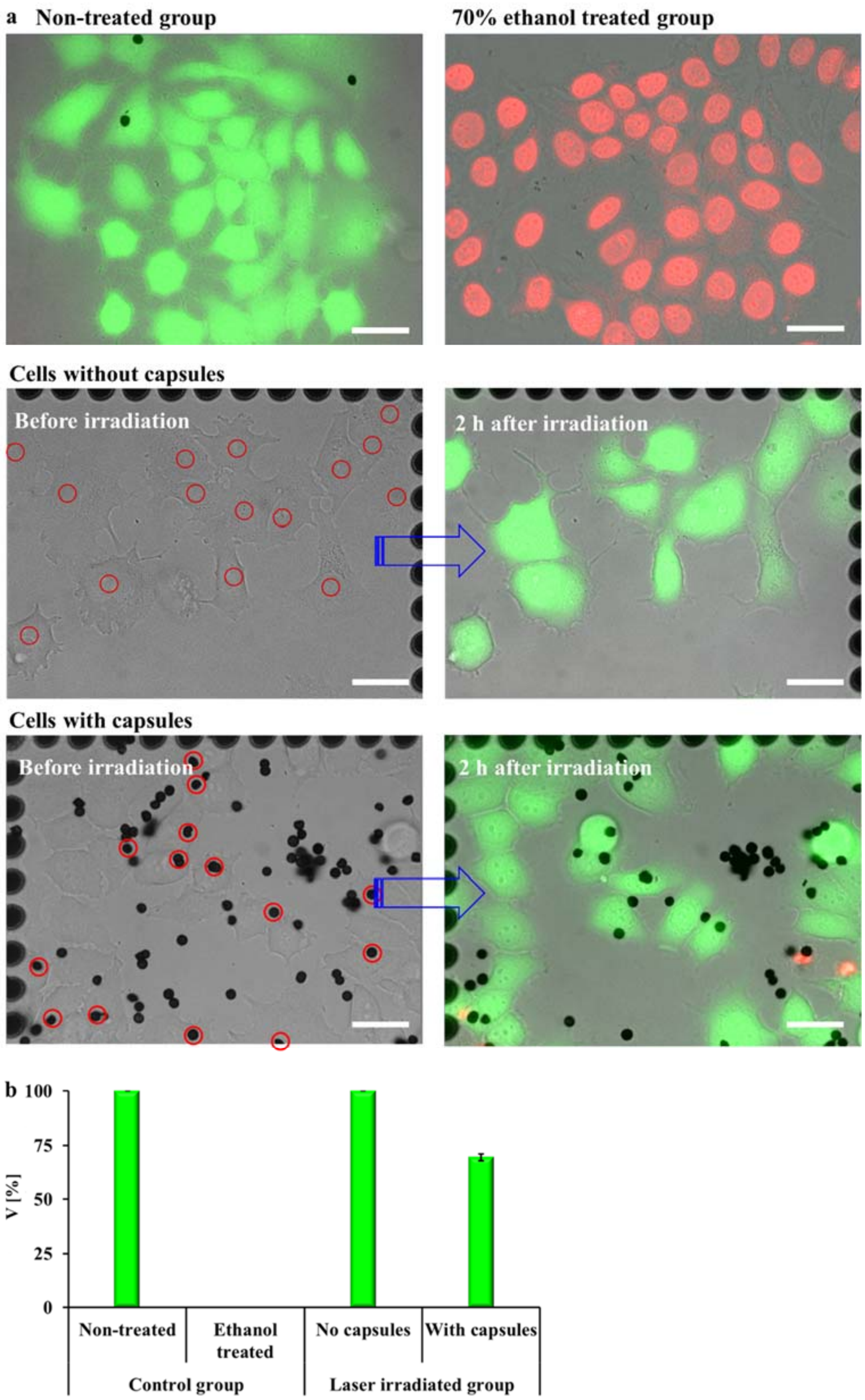

Figure SI.21. Cell viability V after photothermal heating. The experiment was conducted in the widefield microscope set-up. a) Images of non-treated cells, 70% ethanol treated cells, and cells without or with capsules after irradiation. Capsules were excited with an 830 nm laser spot of ca. 2.34 μm^2 (63× objective, widefield microscope) at 0.698 mW (at the illumination spot)

for Δt_{laser} = 2 s. After 2 h, cell viability was determined by live/dead staining. Live cells filled with calcein are shown in green. Dead cells stained with ethidium homodimer-1 are shown in red. The fluorescence channel is overlaid to the bright-field image channel. The red circles indicate irradiated capsules. The scale bars represent 20 μm. b) Cell viability V [%] as mean from three independent experiments from a). More than 120 cells from 3 independent experiments were analyzed.

5. Triggering cytosolic calcium release

5.1 Triggering cytosolic calcium release by adding extracellular ATP

5.2 Triggering cytosolic calcium release by local photothermal heating of star-shaped Au NPs

5.1 Triggering cytosolic calcium release by adding extracellular ATP

Cytosolic calcium can be transiently released by stimulating cells with adenosine 5'-triphosphate (ATP) [13]. We here demonstrated this by directly adding ATP aqueous solution to the cell culture medium during imaging. Briefly, 350,000 MCF-7 or 150,000 HeLa cells were seeded in per petri dish (9.2 cm^2 seeding area) in 2 mL of cell culture medium containing 10% FBS and were incubated overnight. Cells were stained with Fluo-4 as described above in **§3.2** [5]. Afterwards, cells were imaged every 2 s with laser scanning microscopy set-up (LSM880), and 200 µL of 1 mg/mL ATP disodium salt hydrate dissolved in PBS was added directly into the cell culture medium during imaging. The diffusion of ATP to cells was supposed to evoke the cytosolic calcium increase. Two examples from MCF-7 cells and HeLa cells are displayed in **Figure SI.22 and in Figure SI.23**.

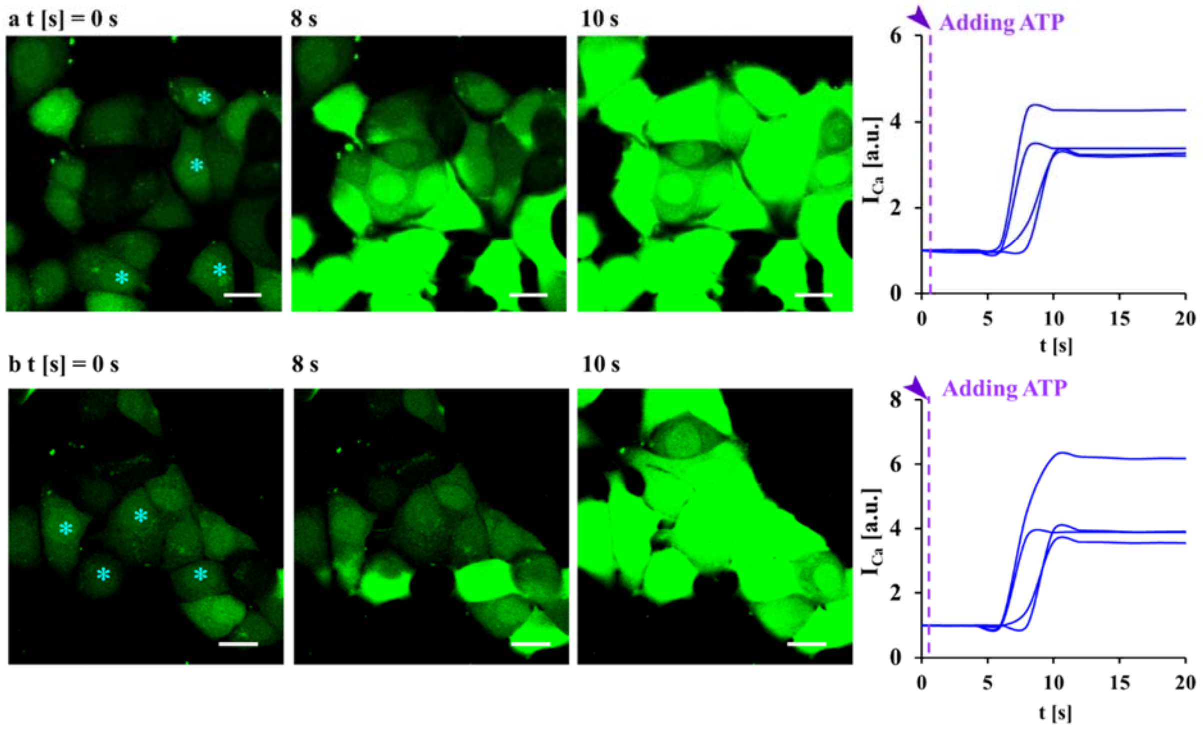

Figure SI.22 ATP triggered cytosolic calcium release in MCF-7 cells. 200 µL of 1 mg/mL ATP in PBS was added directly into 2 mL of cell culture medium during imaging (i.e. the final ATP

concentration was ca. 0.09 mg/mL). Images were taken every 2 s. Two examples are shown. The scale bars represent 20 µm. The integrated fluorescence intensity of the calcium indicator Fluo-4 I_{Ca} over the cross section of the whole cell area was normalized to that before irradiation (t = -2 s). This fluorescence relates to the calcium concentration and is plotted versus time t. The colors of the curves indicate the cells in which the Fluo-4 intensities were measured, as given by the color of the stars labelling the respective cells.

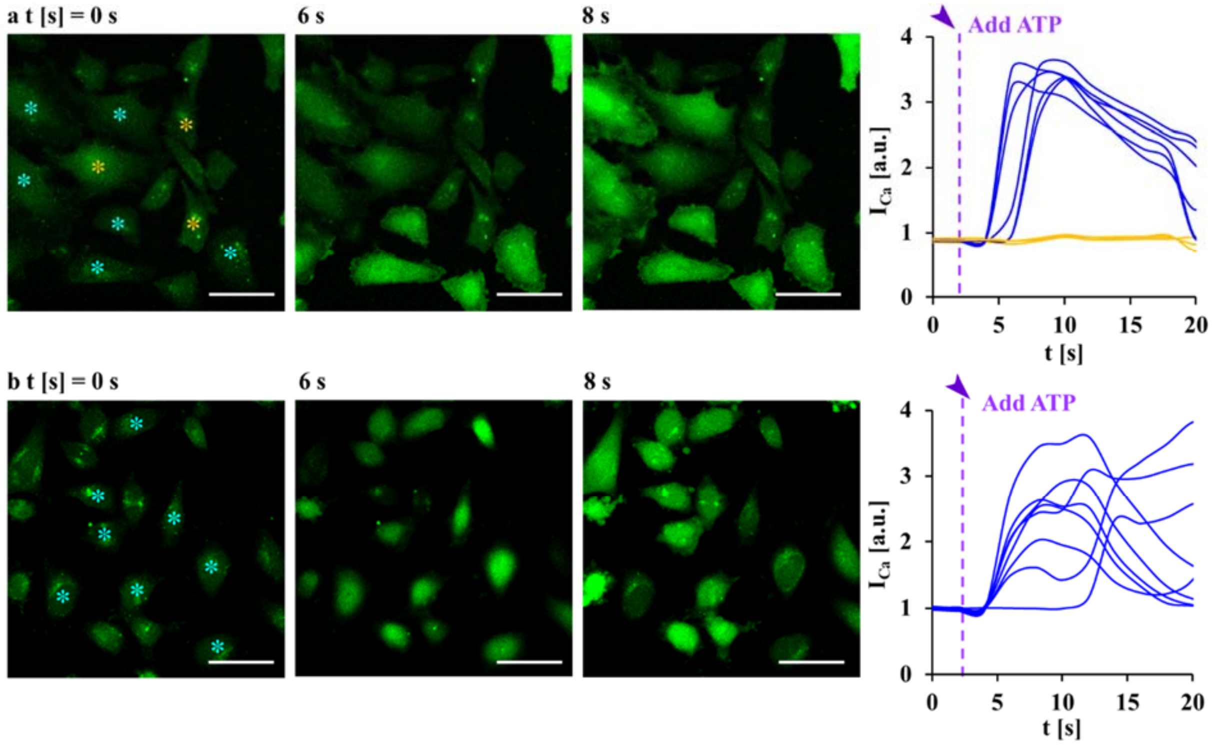

Figure SI.23 ATP triggered cytosolic calcium release in HeLa cells. 200 µL of 1 mg/mL ATP in PBS was added directly into 2 mL of cell culture medium during imaging. Images were taken every 2 s. Two experimental series are shown. The scale bars represent 20 µm. The integrated fluorescence intensity of the calcium indicator Fluo-4 I_{Ca} over the cross section of the whole cell area was normalized to that before irradiation (t = -2 s), and relates to the calcium concentration. The colors of the curves indicate the cells in which the Fluo-4 intensities were measured, as given by the color of the stars labelling the respective cells. Blue stars indicated responsive cells. Yellow stars indicate non-responsive cells.

ATP could also be locally and controlled released from capsules. For this, ATP was encapsulated into the capsules by post-loading and heat-shrinking, see **§1.7**. Upon photothermal heating, capsules open and release ATP. As ATP is required to bind to purinergic receptors on the outer surface of the plasma membrane, capsules should not be endocytosed, which can be achieved by short incubation times. We first irradiated capsules outside cells. Capsules on the

bottom of the petri dish acting as cell substrate were irradiated, but the capsules moved out of focus upon laser irradiation, and thus there was no release of ATP. (**Figure SI.24**). Similar results have been reported before [3a].

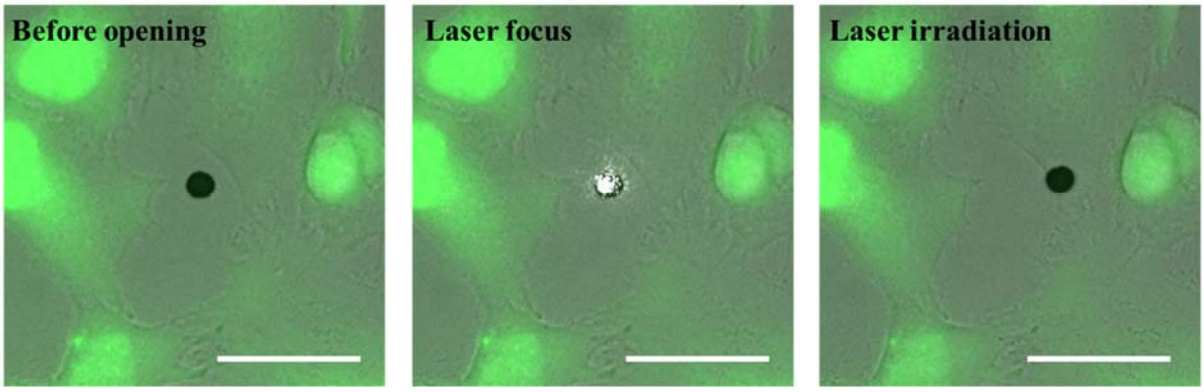

Figure SI.24. Failed photothermal opening of capsules on the bottom of a petri dish upon irradiation by an 830 nm laser spot at P_{laser} = 0.698 mW for Δt_{laser} = 2 s. The capsules moved out of focus upon laser irradiation, and thus there was no release of ATP. The scale bars represent 20 µm. These imaged were recorded with the widefield microscopy set-up.

Hence, the capsules needed to be anchored on the outer surface of the cell membrane to prevent their motion and ensure local ATP release in the extracellular medium. In this case, HeLa cells were used because when one HeLa cell was excited, almost no adjacent HeLa cells responded (**§6.4, §6.5**). For experiments with the widefield microscope set-up, 15,000 HeLa cells were seeded in each well of the 8-well µ-slide (1.0 cm^2 seeding area per well) in 300 µL of cell culture medium containing 10% FBS and were incubated overnight. For the laser scanning microscopy set-up (LSM880), 150,000 HeLa cells were seeded in a petri dish (xx cm^2 seeding area) in 2 mL of cell culture medium containing 10% FBS and were incubated overnight. Afterwards, cells were incubated with freshly prepared capsules encapsulating ATP at a concentration of 2 capsules/cell for 1.5-2 h to allow the association of capsules on the cells. Two examples measured from the widefield microscope and the LSM880 set-up are shown in **Figure SI.25** and **Figure SI.26**, respectively. Data demonstrate that the excitation of intracellular Ca^{2+} release as quantified by Fluo-4 fluorescence is due to the released ATP, and not due to the photothermal heating itself. Photothermal heating of capsules itself initiates release of intracellular Ca^{2+} in the cell where the capsule is located, see for example **Figure SI.17**. However, in adjacent cells, which are not connected to the stimulated cells, there is no release of intracellular Ca^{2+} on the same time scale, and thus no effect of photothermal heating.

There may be a slower response in those cells, which however is to ATP signalling, see for example **Figure SI.41**.

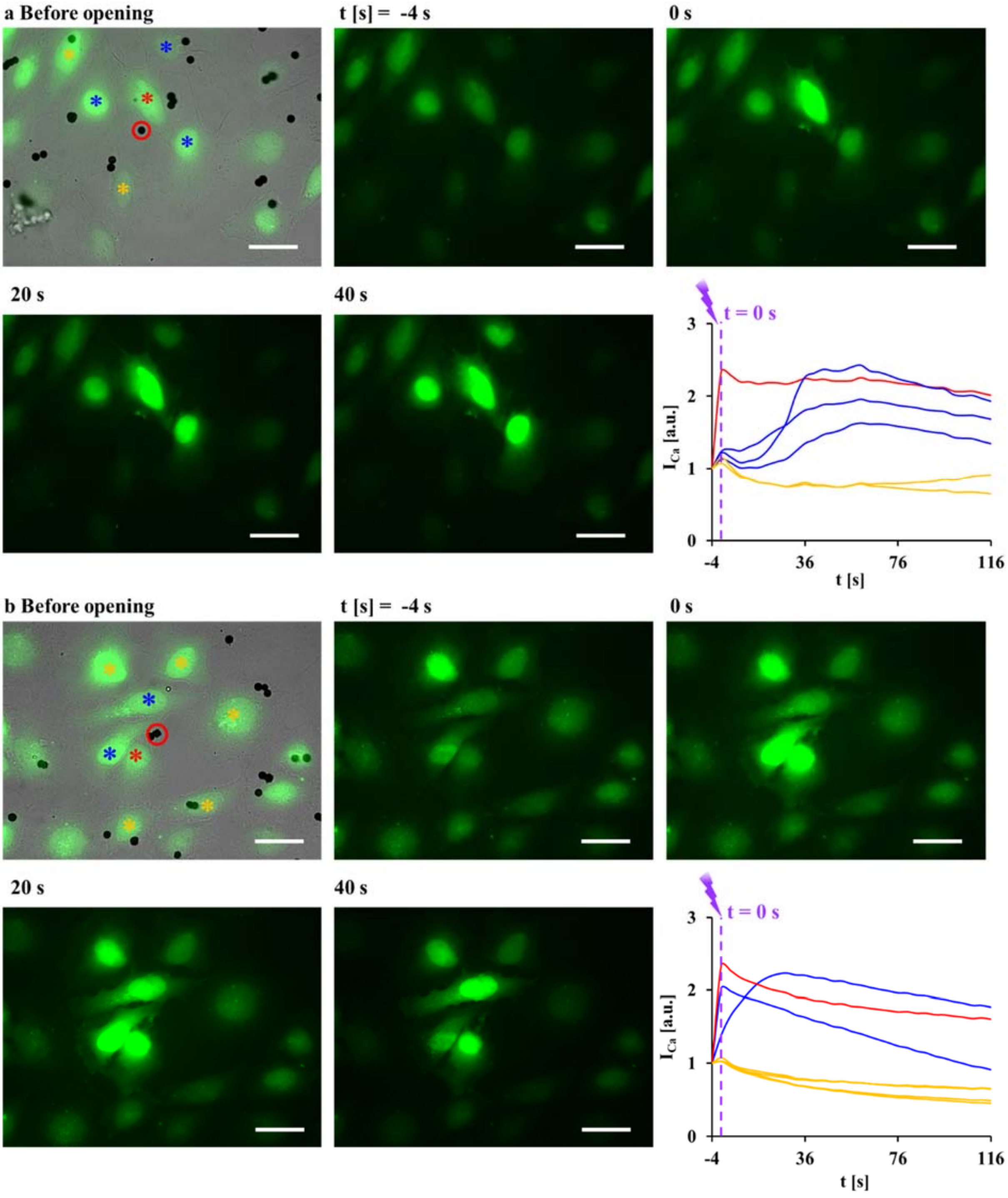

Figure SI.25 At time t = 0 s one capsule associated on the outer surface of a HeLa cell was irradiated with the 830 nm laser (63× objective, widefield microscope set-up) at P_{laser} = 0.698 mW for Δt_{laser} = 2 s. The irradiation regions are indicated by the red circles. Two examples are shown. Images were taken every 4 s. The scale bars represent 20 µm. The integrated fluorescence intensity of the calcium indicator Fluo-4 I_{Ca} over the cross section of the whole

cell area was normalized to that before irradiation (t = -4 s), and is plotted versus time t. The colors of the curves indicate the cells in which the Fluo-4 intensities were measured, as given by the color of the stars labelling the respective cells. The red star indicates the cell with associated capsules. Blue stars indicate responsive cells. Yellow stars indicate non-responsive cells.

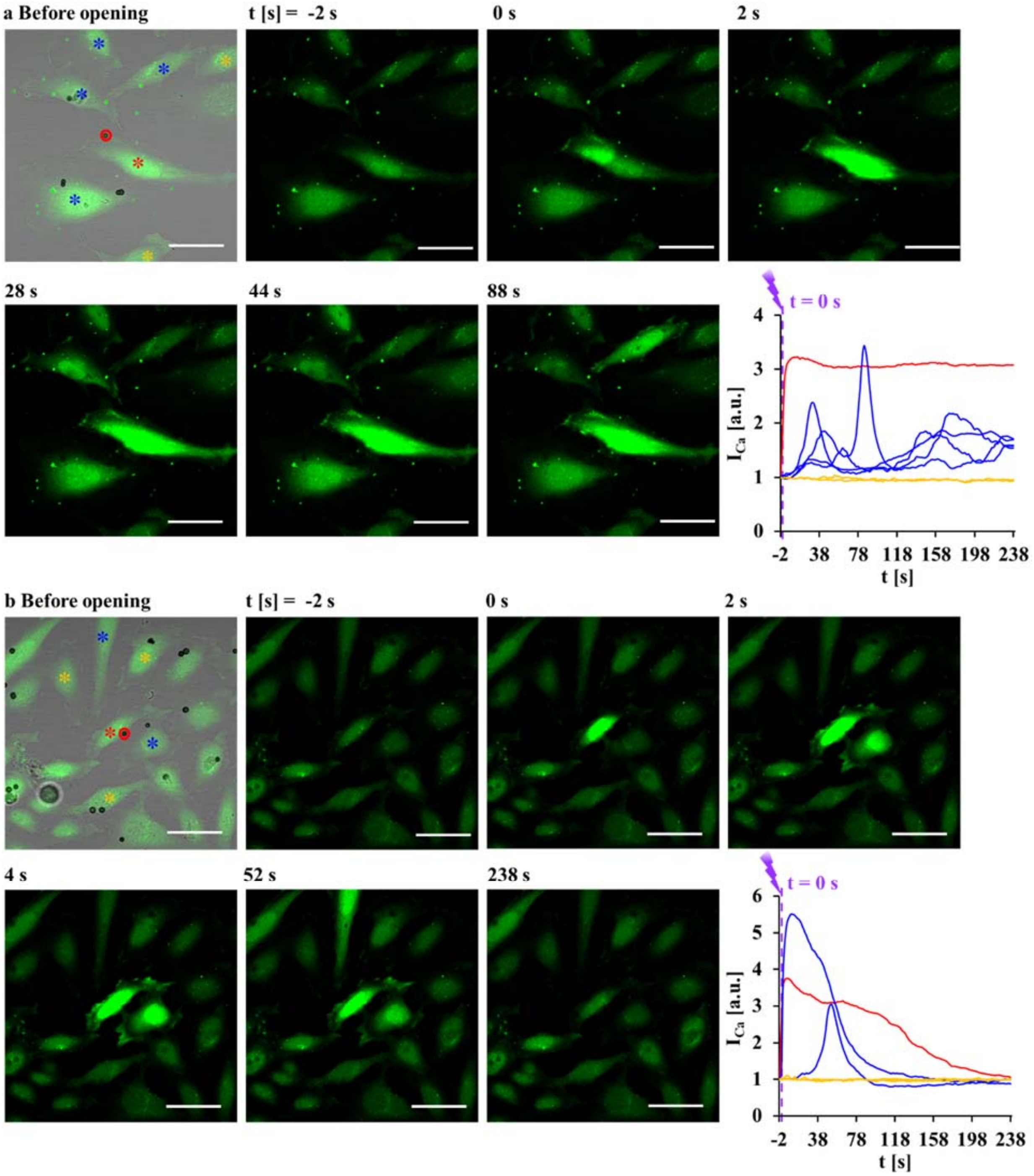

Figure SI.26 At time t = 0 s one capsule associated on the outer surface of a HeLa cell was irradiated with a 830 nm laser (20× objective, LSM880 set-up) at P_{laser} = 28.5 mW for Δt_{laser} = 0.039 s. The irradiated regions are indicated by the red circles. Two examples are shown.

Images were taken every 2 s. The scale bars represent 20 µm. The integrated fluorescence intensity of the calcium indicator Fluo-4 I_{Ca} over the cross section of the whole cell area was normalized to that before irradiation (t = -2 s), and is plotted versus time t. The colors of the curves indicate the cells in which the Fluo-4 intensities were measured, as given by the color of the stars labelling the respective cells. The red star indicates the cell with associated capsules, which was irradiated by the laser. Blue stars indicate responsive cells. Yellow stars indicate non-responsive cells.

5.2 Triggering cytosolic calcium release by local photothermal heating of encapsulated star-shaped Au NPs

Photothermal heating of endocytosed Au NPs has been reported to induce cytosolic calcium release without reducing cell viability [14]. Herein, we demonstrate that photothermal heating of internalized star-shaped Au NPs can evoke transient increase in cytosolic calcium.

Widefield microscopy set-up: For experiments 15,000 MCF-7 cells were seeded in each well of a 8-well µ-slide (1.0 cm^2 seeding area per well) in 300 µL of cell culture medium containing 10% FBS and were incubated overnight. Afterwards, 60 µL of star-shaped Au NPs (0.244 mg/mL) were added to each well at a final concentration of 40 µg/mL, and cells were incubated overnight. Note, here plain Au NPs were added, not encapsulated Au NPs. Cells were stained with Fluo-4 as described above in **§3.2**. In **Figure SI.27** date for an isolated single cell are shown which was irradiated at P_{laser} = 2.65 mW (63× objective, widefield microscope) for Δt_{laser} = 5 s. The irradiation energy E_{laser} = 13.25 mJ was much higher as compared to the one which was used for the photothermal heating of encapsulated Au NPs. Of note, only in few cases cytosolic calcium increase was observed, probably due to the inefficient irradiation energy.

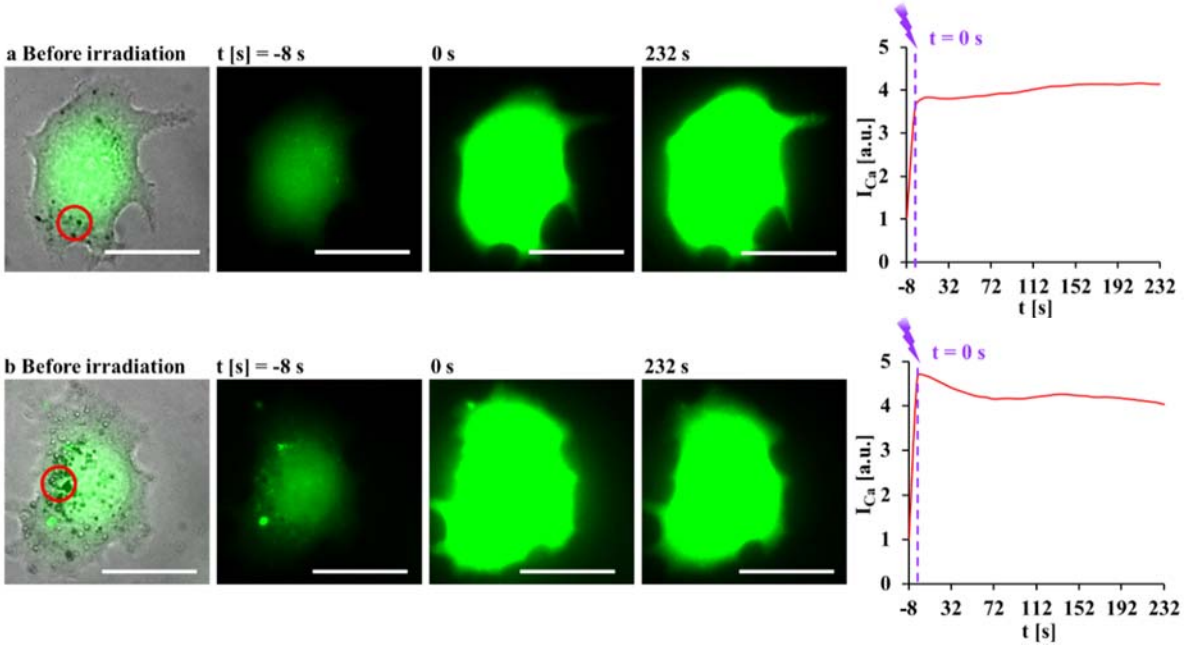

Figure SI.27. At time t = 0 s one single MCF-7 cell with internalized star-shaped Au NPs was irradiated with a 830 nm laser spot whose area was 2.34 μm^2 (63× objective, widefield microscope set-up) at P_{laser} = 2.65 mW for Δt_{laser} = 5 s. The irradiation regions are indicated by red circles. Two examples are shown. Images were taken every 8 s. The scale bars represent 20 μm. The integrated fluorescence intensity of the calcium indicator Fluo-4 I_{Ca} over the cross section of the whole cell area was normalized to that before irradiation (t = -8 s), and is plotted versus time t.

<u>Laser-scanning microscopy set-up (LSM880):</u> The same experiment was carried out also with the LSM880 set-up. 150,000 MCF-7 cells were seeded in a petri dish (9.2 cm^2 surface area) in 2 mL of cell culture medium containing 10% FBS and were incubated overnight. Afterwards, 400 μL of star-shaped Au NPs (0.244 mg/mL) were added to each well at a final concentration of 40 μg/mL, and cells were incubated overnight. Cells were stained with Fluo-4. In **Figure SI.28**, irradiation of an isolated single cell at P_{laser} = 142.5 mW for an average time Δt_{laser} = 270 ms is shown. Thus, the total irradiation energy E_{laser} = 38.5 mJ. Of note, the parameters were comparable to that of the control group in **Section 4.2.2**. Cells were imaged every 2 s for 60 times. A comparison of irradiation parameters between capsules with integrated star-shaped Au NPs and star-shaped Au NPs is shown in **Table S2**. Of note, almost all the irradiated cells responded with an increase in free cytosolic calcium, which is different from the situation observed in widefield microscope. This is because an almost 2.9 times higher energy E_{laser} was applied in the LSM880 compared to the widefield microscope set-up.

	Capsules with star-shaped Au NPs		Star-shaped Au NPs
Δt_{laser} [s]	0.039	0.039	0.27[a]
A_{laser} [μm^2]	12.56	12.56	88.88[a]
P_{laser} [%]	1.2	2.5	5
P_{laser} [mW]	34.2	71.25	142.5
I_{laser} [W/m^2]	2.72×10^9	5.67×10^9	1.60×10^9
E_{laser} [mJ]	1.33	2.78	38.48
U_{laser} [J/m^2]	1.06×10^8	2.21×10^8	4.33×10^8

Table S2. Comparison of irradiation parameters between capsules and star-shaped Au NPs. [a]: The averaged irradiation area and time are shown here. These two parameters are slightly different in different cases.

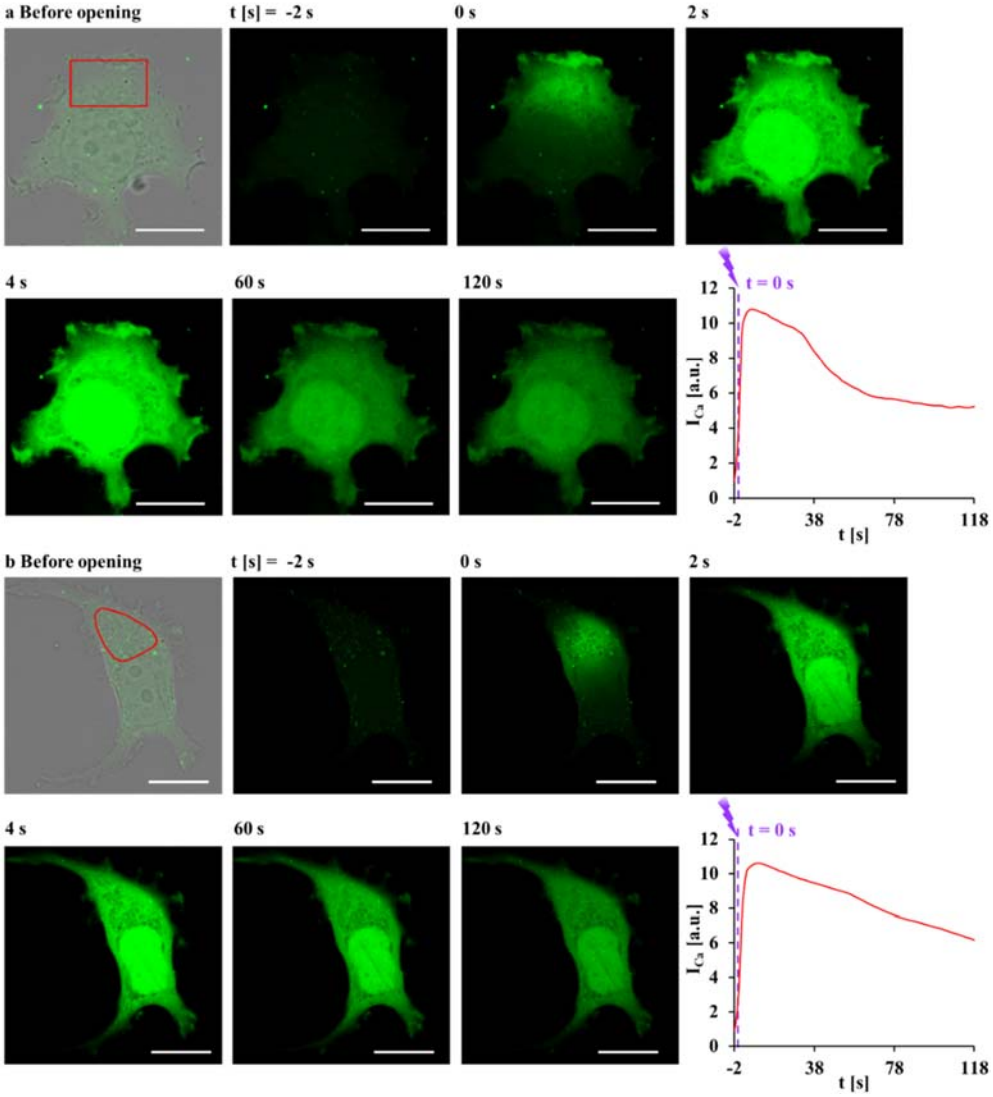

Figure SI.28. At time t = 0 s one single MCF-7 cell which had internalized star-shaped Au NPs was irradiated with a 830 nm laser (20× objective, LSM880 set-up) at P_{laser} = 142.5 mW for

Δt_{laser} = 0.265 s. The irradiated regions are indicated by the red boxes. Images were taken every 2 s. The scale bars represent 20 μm. The integrated fluorescence intensity of the calcium indicator Fluo-4 I_{Ca} over the cross section of the whole cell area was normalized to that before irradiation (t = -2 s), and is plotted versus time t.

6. Measuring cytosolic calcium increase upon photothermal heating of polymer capsules with integrated star-shaped Au NPs

6.1 Cytosolic calcium increase in isolated single MCF-7 or HeLa cells
6.2 pH change in cytosol and lysosome in isolated single MCF-7 cells after photothermal heating
6.3 Preliminary experiments for investigating the origin of cytosolic calcium increase upon photothermal heating
6.4 Cytosolic calcium increase in MCF-7 or HeLa cells which were not in direct contact with each other
6.5 Cytosolic calcium increase in MCF-7 or HeLa cells which were physically in contact with adjacent cells
6.6 Cytosolic calcium increase in co-cultured MCF-7/HeLa or MCF-7/NIH 3T3 cells which were physically in contact with adjacent cells

6.1 Cytosolic calcium increase in isolated single MCF-7 or HeLa cells

Widefield microscopy set-up: 15,000 MCF-7 cells or 7,500 HeLa cells were seeded in each well of a 8-well µ-slide (1.0 cm^2 seeding area per well) in 300 µL of cell culture medium containing 10% FBS and were incubated overnight. On the next day, hollow capsules with embedded star-shaped Au NPs were added at a density of 2 capsules/seeded cell, and cells were incubated overnight. Cells were stained with Fluo-4. For photothermal heating the target capsules were irradiated at P_{laser} = 0.622 mW for Δt_{laser} = 2 s. Images were taken every 8 s for 30 times using the 100× objective in widefield microscope. Data are shown in **Figure SI.29**.

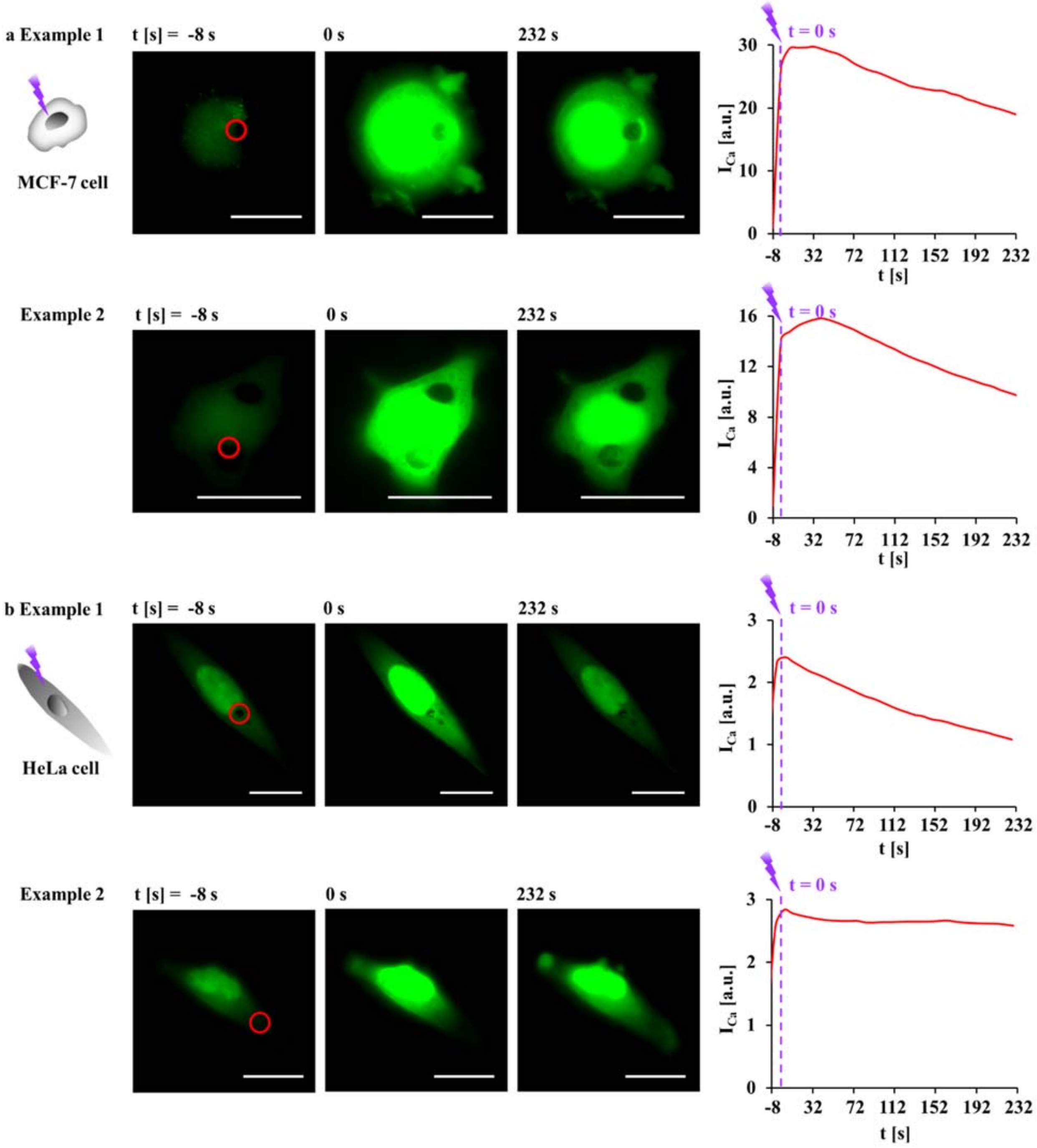

Figure SI.29 At time t = 0 s one capsule with embedded star-shaped Au NPs as indicated by the red circle, as endocytosed by a a) MCF-7 and b) HeLa cell was exited with an 830 nm laser spot of A_{laser} = 1.17 μm^2 (100× objective, widefield micrscope) at 0.622 mW (at the illumination spot) for Δt_{laser} = 2 s. Images were taken every 8 s. Two examples are demonstrated for each cell line. The scale bars represent 20 μm. The integrated fluorescence intensity of the calcium indicator Fluo-4 I_{Ca} over the cross section of the whole cell area was normalized to that before irradiation (t = -8 s), and relates to the calcium concentration. I_{Ca} is plotted versus time t.

For repetitive stimulation, the same isolated single MCF-7 cell was repetitively excited 3 times. Cells were stained with Fluo-4, and excited with an 830 nm laser spot of ca. 1.17 μm^2 (100× objective, widefield micrscope) at 0.622 mW (at the illumination spot) for Δt_{laser} = 2 s at t = 0 s, 780 s and 1560 s. Images were taken every 15 s for 10 frames using the 100× objective in widefield microscope. Data are shown in **Figure SI.30**.

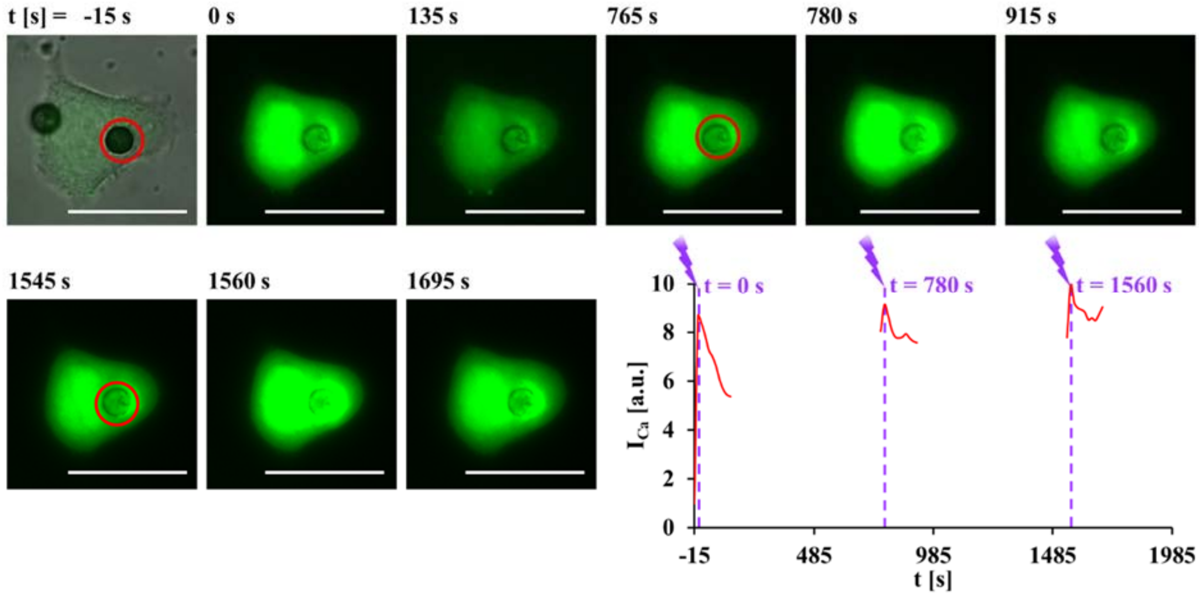

Figure SI.30 At times t = 0 s, 780 s, and 1560 s one capsule with embedded star-shaped Au NPs, as endocytosed by a MCF-7 cell and as indicated by the red circle, was excited with an 830 nm laser of ca. 1.17 μm^2 spot size (100× objective, widefield microscope) at P_{laser} = 0.622 mW (at the illumination spot) for Δt_{laser} = 2 s. Images were taken every 15 s. The scale bars represent 20 μm. The integrated fluorescence intensity of the calcium indicator Fluo-4 I_{Ca} over the cross section of the whole cell area was normalized to that before irradiation (t = -15 s), and is plotted versus time t.

Laser-scanning microscopy set-up (LSM880): 150,000 MCF-7 cells or 75,000 HeLa cells were seeded in a petri dish (9.2 cm^2 seeding area) in 2 mL of cell culture medium containing 10% FBS and were incubated overnight. On the next day, hollow capsules embedded with star-shaped Au NPs were added at a density of 2 capsules/cell, and cells were incubated overnight. Cells were stained with Fluo-4, and excited at 830 nm with an irradiation area of ca. 12.56 μm^2 (20× objective, LSM880) at P_{laser} = 34.2 mW (at the illumination spot) for Δt_{laser} = 0.039 s. Images were taken every 2 s for 60 times using a 20× objective in the LSM880 set-up. Data are shown in **Figure SI.31**.

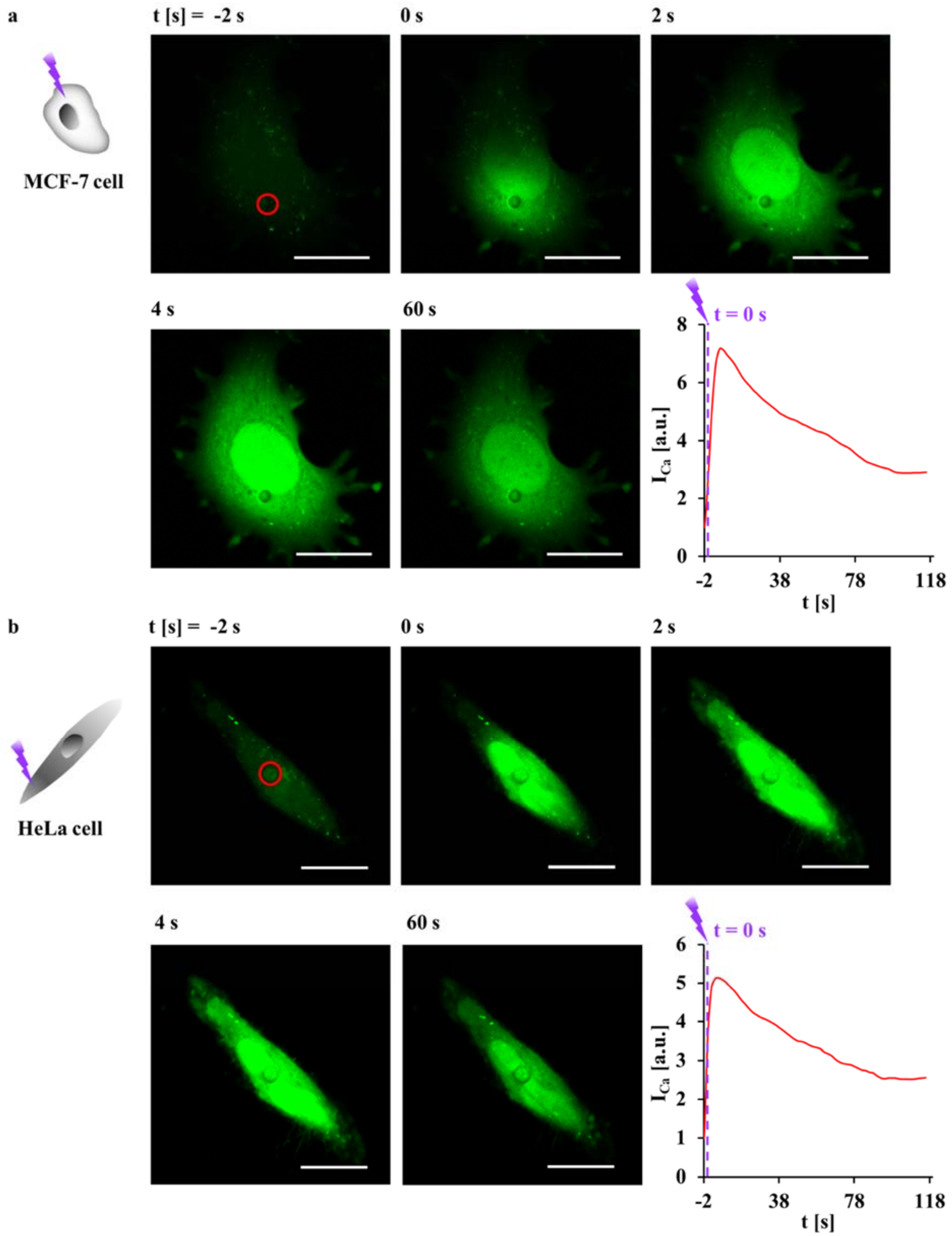

Figure SI.31. At time t = 0 s one capsule with embedded star-shaped Au NPs, as endocytosed by a a) MCF-7 and b) HeLa cell and as indicated by the red circle was excited at 830 nm with and irradiation area of ca. 12.56 µm^2 (20× objective, LSM880) at P_{laser} = 34.2 mW (at the illumination spot) for Δt_{laser} = 0.039 s. Images were taken every 2 s. The scale bars represent 20 µm. The integrated fluorescence intensity of the calcium indicator Fluo-4 I_{Ca} over the cross

section of the whole cell area was normalized to that before irradiation (t = -2 s), and is plotted versus time t.

For repetitive stimulation, the same isolated single MCF-7 cell was repetitively excited for 3 times. Cells were stained with Fluo-4 and excited at 830 nm with an irradiation area of ca. 12.56 μm^2 (20× objective, LSM880) at P_{laser} = 34.2 mW (at the illumination spot) for Δt_{laser} = 0.039 s. Images were taken every 2 s (**Figure SI.32 a**), or every 4 s (**Figure SI.32 b**) for 150 times using the 20× objective in the LSM880. Two capsules in different positions were irradiated in **Figure SI.32a** (one capsule was irradiated in each time), while in **Figure SI.32b** the same capsule was irradiated for three times.

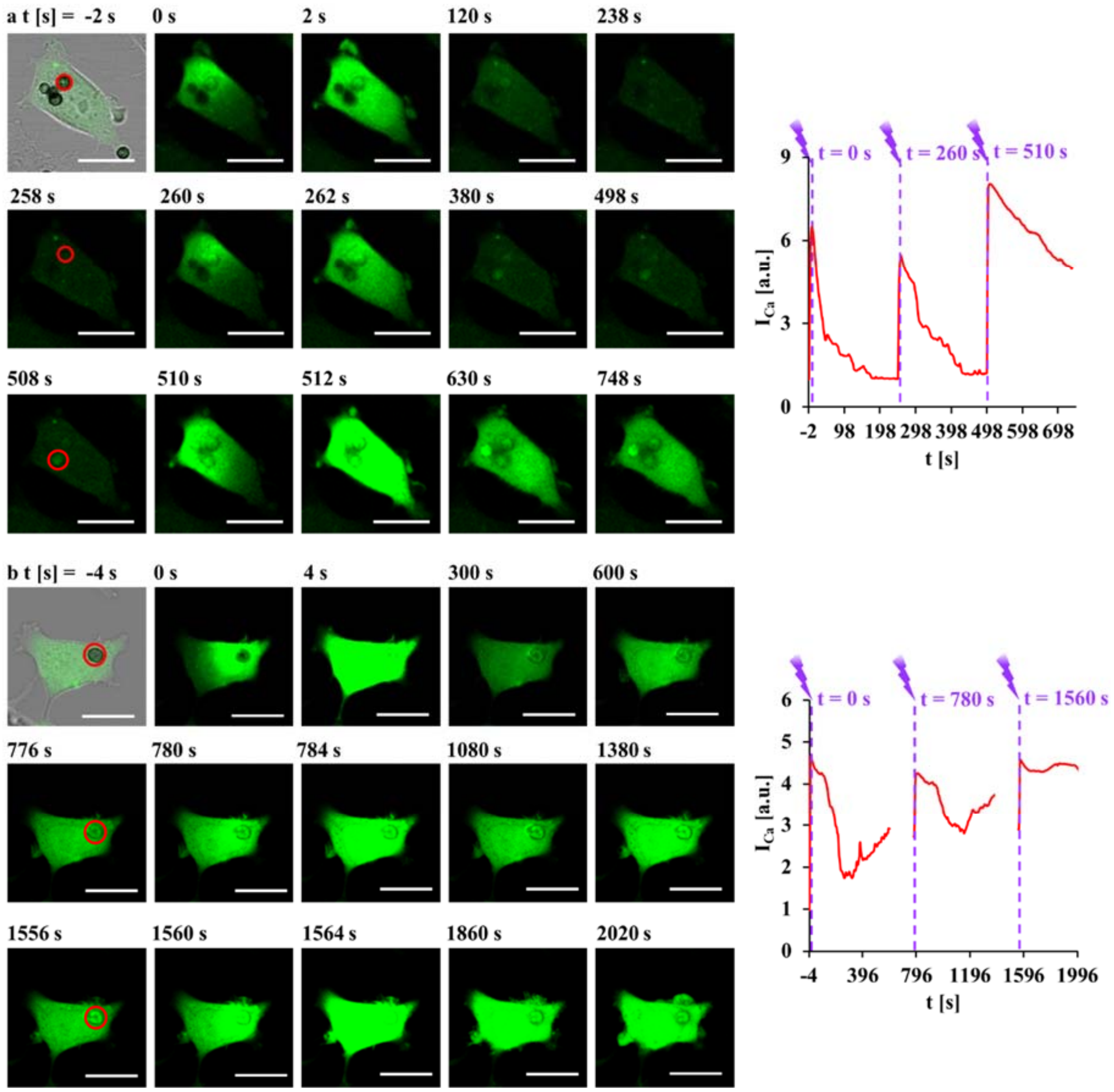

Figure SI.32. At time t = 0 s one capsule with integrated star-shaped Au NPs (as indicated by the red circle), which had been endocytosed by either a) a MCF-7 or b) a HeLa cell was irradiated at 830 nm with irradiation area of ca. 12.56 μm^2 (20× objective, LSM880) at P_{laser} = 34.2 mW (at the illumination spot) for Δt_{laser} = 0.039 s. Images were taken every a) 2 s or b) 4 s. Two examples are demonstrated here. The scale bars represent 20 μm. The integrated fluorescence intensity of the calcium indicator Fluo-4 I_{Ca} over the cross section of the whole cell area was normalized to that before irradiation (a) t = -2 s, b) t = -4 s) is plotted versus time t.

6.2 pH change in cytosol and lysosome in isolated single MCF-7 cells after photothermal heating

The perforation of lysosomes surrounding the endocytosed capsules upon photothermal heating may release H_3O^+ from the highly acidic lysosomes into the cytosol, resulting in decreased cytosolic pH. The pH in the lysosomes may also increase at the same time due to dilution with the neutral solution of the cytosol. To check this, an isolated single MCF-7 cell was stained with Fluo-4 (which is a Ca^{2+} indicator) and SNARF-1 (which is a pH indicator) simultaneously, and change in Ca^{2+} and pH upon photothermal heating was probed. 150,000 MCF-7 cells were seeded in a petri dish (9.2 cm^2 seeding area) in 2 mL of cell culture medium containing 10% FBS and were incubated overnight. On the next day, hollow capsules with integrated star-shaped Au NPs were added at a density of 2 capsules/cell and cells were incubated overnight to ensure capsule internalization. One vial of 5-(and-6)-carboxy SNARF™-1 acetoxymethyl ester (SNARF-1, #C1272, ThermoFisher) containing 50 μg SNARF-1 was dissolved in 29.3 μL of anhydrous DMSO to prepare a 3 mM stock solution. The stock solution was stored at -20 °C and was protected from light. Before microscopy measurements cells were washed with PBS for 2 times, and incubated with working solution at room temperature for 30 min, which contained 3 μL of 3 mM SNARF-1, 3.9 μL of 1.5 mM Fluo-4 AM and 900 μL of PBS. Afterwards, cells were washed with PBS for 2 times, and incubated at 37 °C in 2 mL of cell culture medium containing 10% FBS for 15 min before use.

For photothermal excitation cells were irradiated at 830 nm with a irradiation area of A_{laser} = 12.56 μm^2 (20× objective, LSM880) at P_{laser} = 34.2 mW (at the illumination spot) for Δt_{laser} = 0.039 s. Images were taken every 2 s for 120 times using the 20× objective in the LSM880 set-up. The imaging parameters are discussed in **Section 4.1**. Data are shown in **Figure SI.33.** The

ration of the red to yellow fluorescence intensity (I_r/I_y) of the SNARF-1 fluorescence reflects the pH value and a high I_r/I_y ratio indicates high pH [6-7].

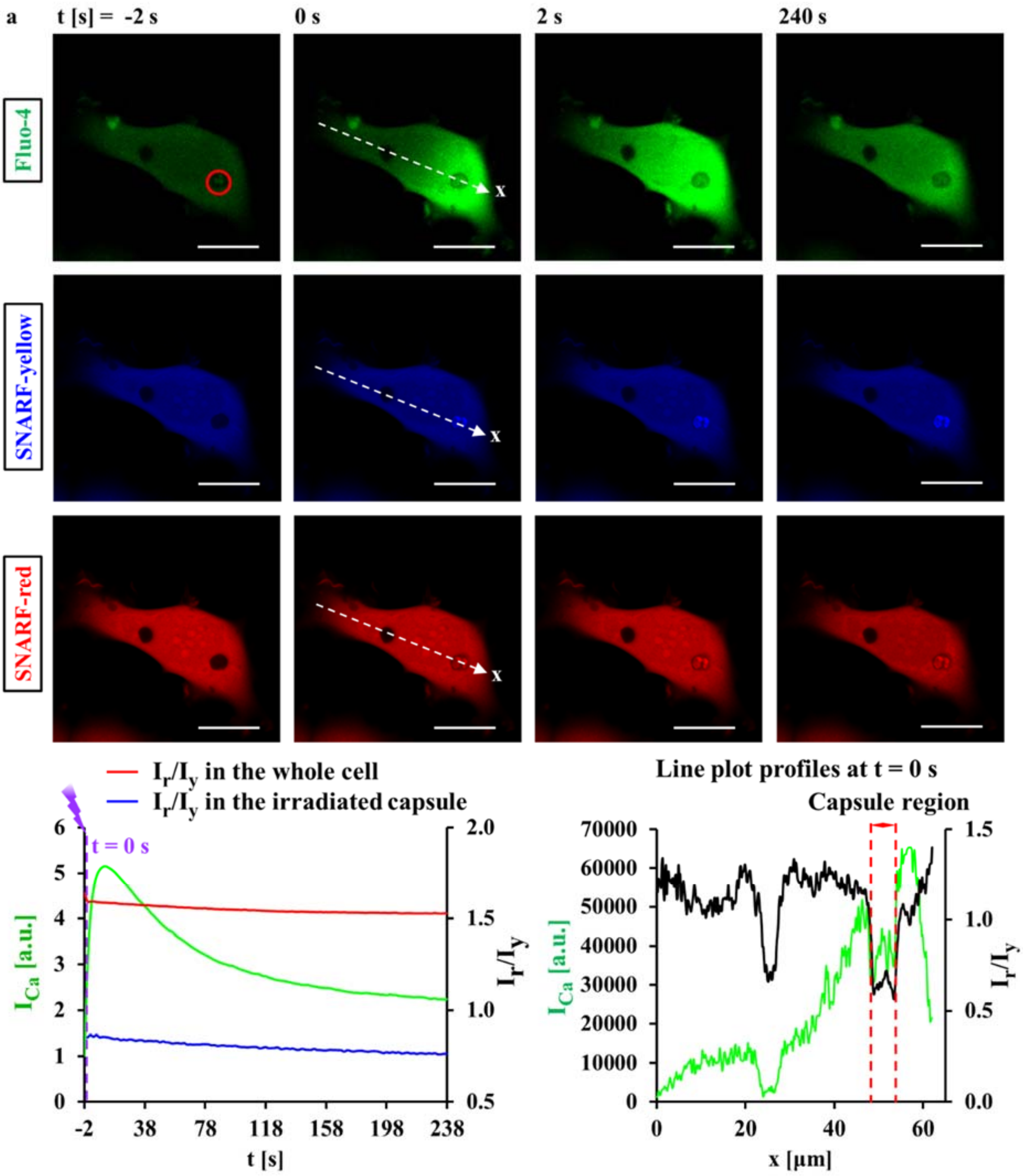

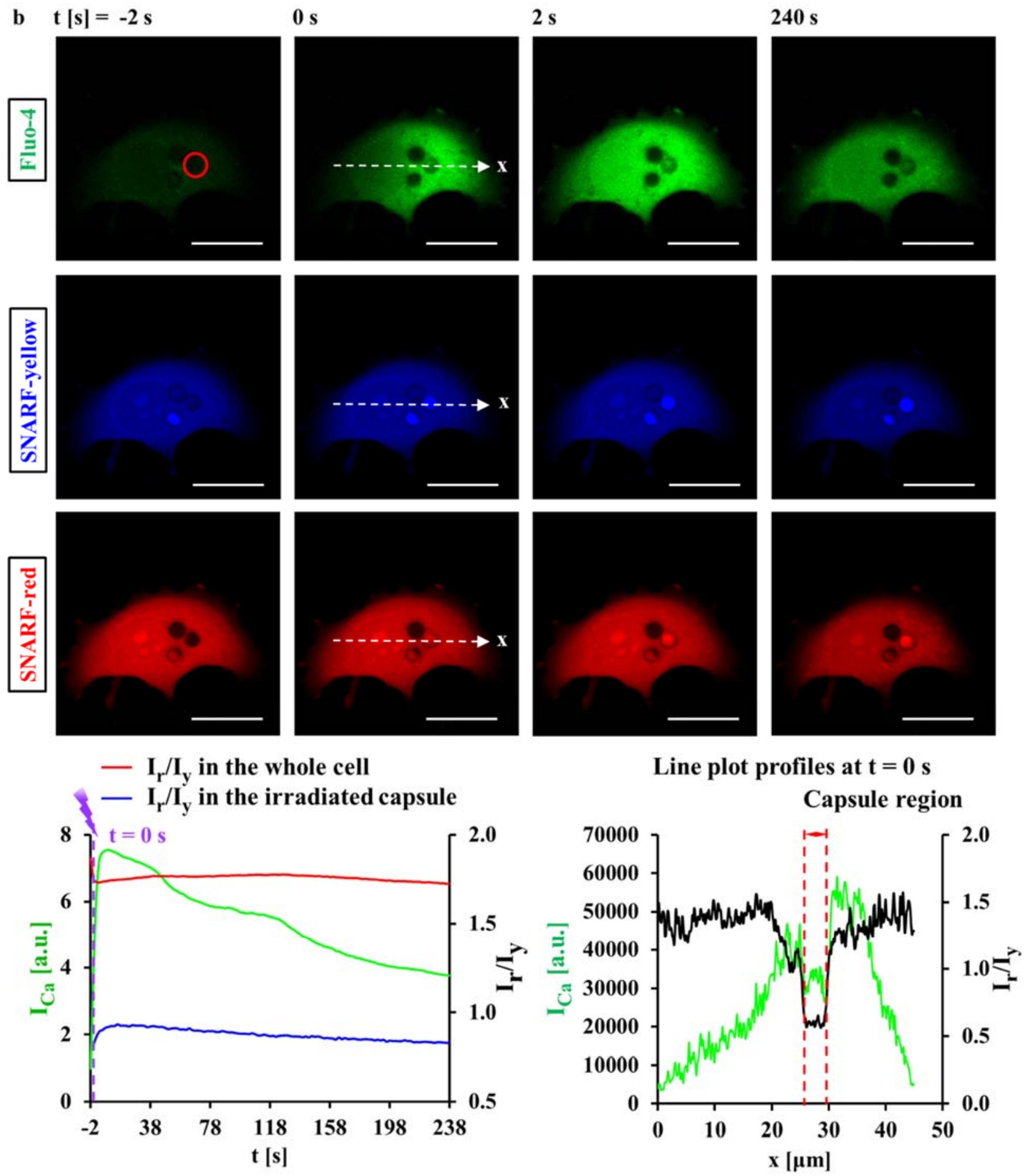

Figure SI.33. At time t = 0 s one capsule with embedded star-shaped Au NPs, as endocytosed by a MCF-7 cell and as indicated by the red circle was irradiated at 830 nm with an irradiation area of ca. 12.56 µm^2 (20× objective, LSM880) at P_{laser} = 34.2 mW (at the illumination spot) for Δt_{laser} = 0.039 s. Two examples are demonstrated here. Images were taken every 2 s. The scale bars represent 20 µm. The integrated fluorescence intensity of the calcium indicator Fluo-4 I_{Ca} (shown in green) over the cross section of the whole cell area was normalized to that before irradiation t = -2 s, and is plotted versus time t. The red-to-yellow ratio of the SNARF-1 fluorescence signals I_r/I_y from the location of the cellular cytosol and the irradiated capsule are

also plotted versus time t. These intensities indicate the local pH values. Upon photothermal excitation the pH in the cytosol does not drop significantly, and thus the change in Fluo-4 intensity cannot be attributed to a pH change but must be due to a change in free Ca^{2+}.

In additional experiments cells were excited at 830 nm with an irradiation area of ca. 12.56 μm^2 (20× objective, LSM880) at P_{laser} = 57 mW (at the illumination spot) for Δt_{laser} = 0.004 s (E_{laser} = 0.228 mJ). Of note, Δt_{laser} is here much shorter than in the experiments reported in **Figure SI.33** (Δt_{laser} = 0.004 s versus Δt_{laser} = 0.039 s), which is caused by the much faster scanning (60 ms versus 1.26 s), and the irradiation energy is also reduced (E_{laser} = 0.228 mJ versus E_{laser} = 1.338 mJ). This condition was also sufficient to trigger intracellular Ca^{2+} release. Images were taken every 0.06 s for 120 times using a 20× objective in the LSM880 set-up. 2 examples are demonstrated in Figure **SI.34**. Directly after photothermal excitation the I_r/I_y ratio closely around the excited capsule/lysosome dropped, which indicates raise in pH, e.g. the local region of the opened lysosome is acidified. This local temporal acidification as quantified by $I_r/I_y(x)$ decays on the lengthscale of a few micrometers, i.e. the pH in the bulk of the lysosome is not affected. On the timescale of a few seconds $I_r/I_y(t)$ the acidification close to the opened lysosome also has relaxed back to the neutral lysosomal pH.

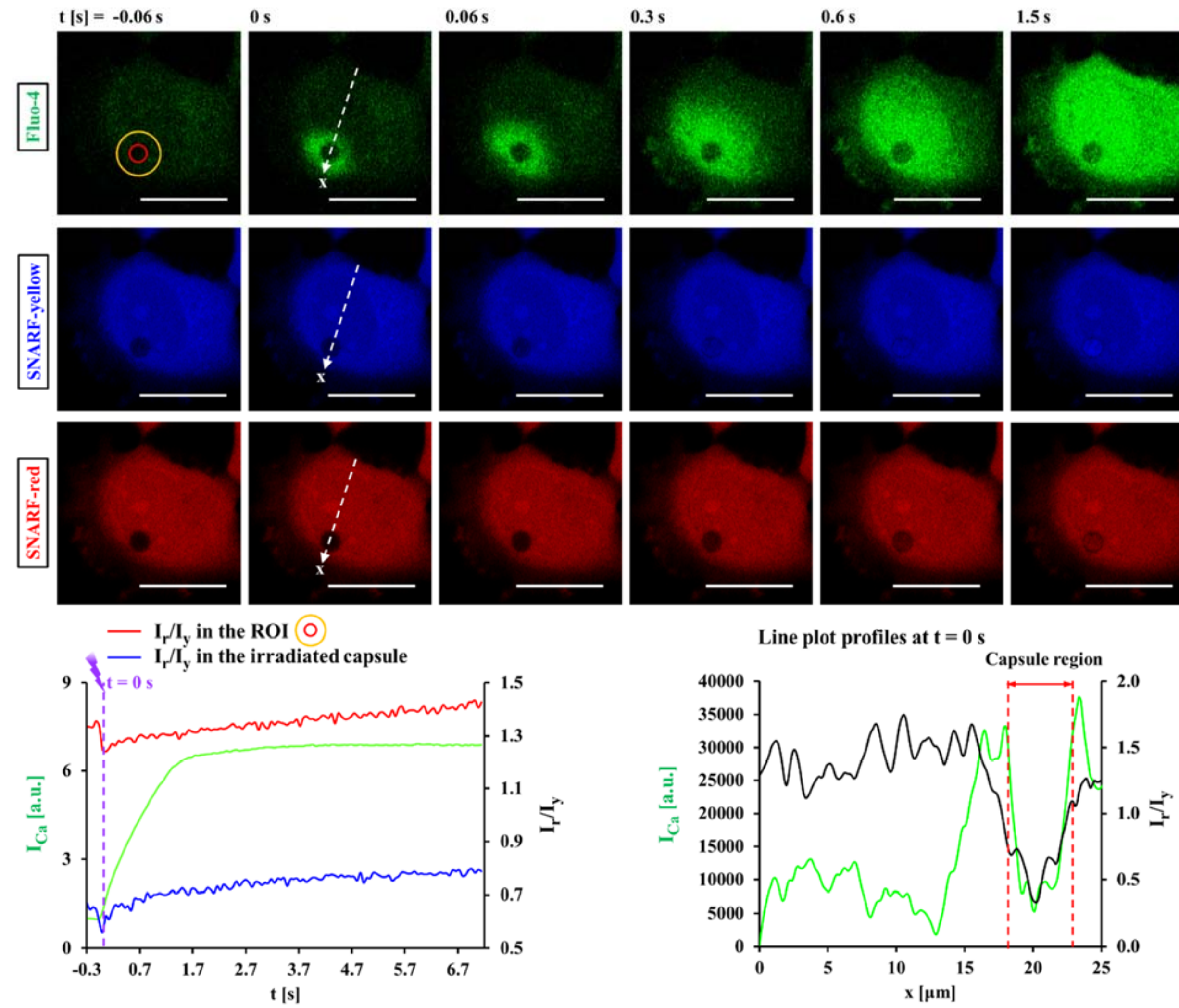

t [s] = -0.06 s
0 s
0.06 s
0.3 s
0.6 s
1.5 s
Fluo-4
SNARF-yellow
SNARF-red
x
I_r/I_y in the ROI
I_r/I_y in the irradiated capsule
t = 0 s
I_{Ca} [a.u.]
I_r/I_y
t [s]
Line plot profiles at t = 0 s
Capsule region
x [µm]

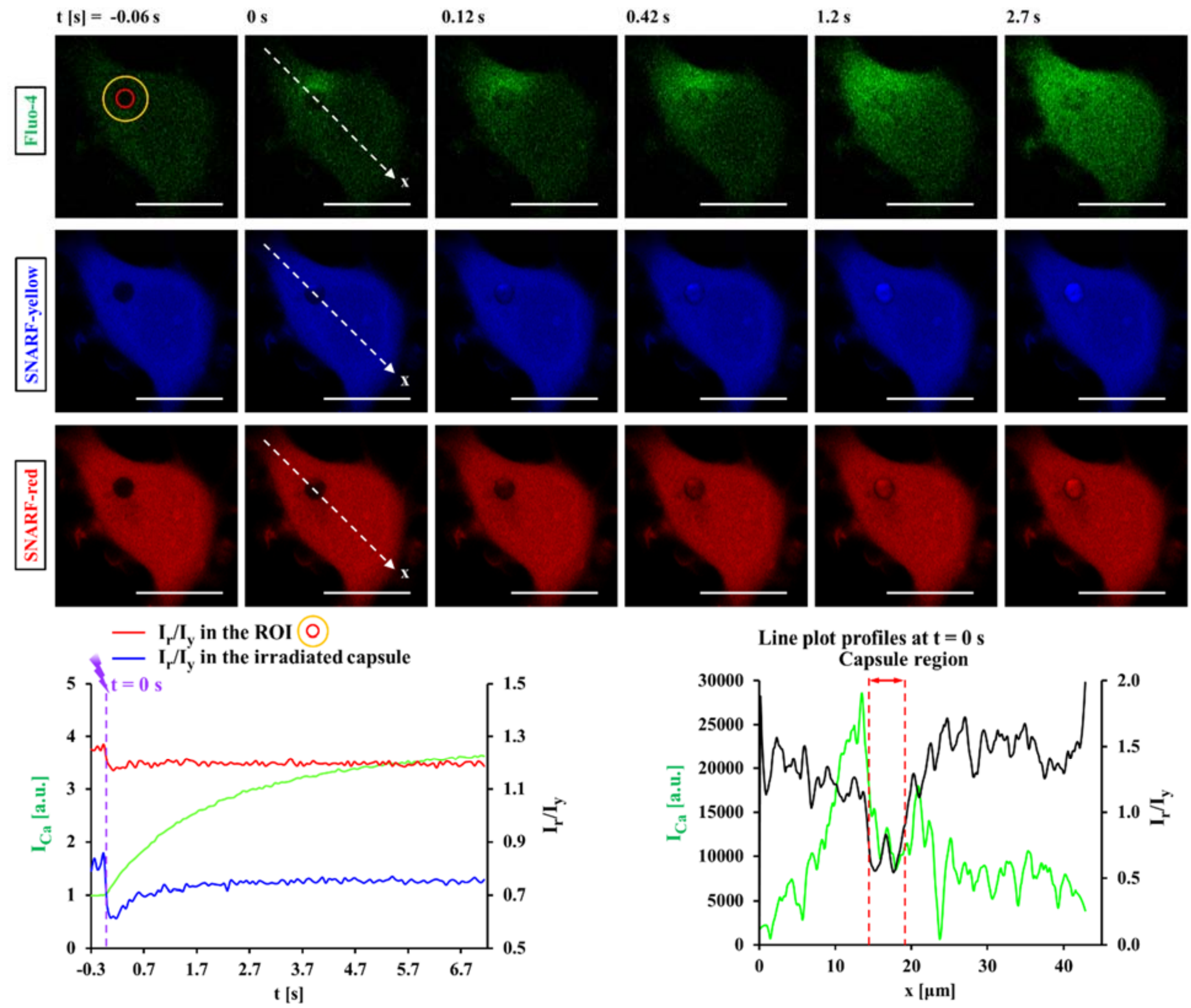

Figure SI.34. At time t = 0 s one capsule with embedded star-shaped Au NPs, as endocytosed by a MCF-7 cell and as indicated by the red circle was irradiated at 830 nm with an irradiation area of ca. 12.56 µm^2 (20× objective, LSM880) at P_{laser} = 57 mW (at the illumination spot) for Δt_{laser} = 0.004 s. Two examples are demonstrated here. Images were taken every 0.06 s. The scale bars represent 20 µm. The integrated fluorescence intensity of the calcium indicator Fluo-4 I_{Ca} (shown in green) over the cross section of the whole cell area was normalized to that before irradiation t = -0.06 s, and is plotted versus time t. This fluorescence intensity corresponds to the intracellular Ca^{2+} concentration $[Ca^{2+}]_i$. The red-to-yellow ratio of the SNARF-1 fluorescence signals I_r/I_y from the location of the irradiated capsule and the surround region of interest (ROI, the area between yellow and red circles) are also plotted versus time t. I_r/I_y indicate the local pH values. I_{Ca} and I_r/I_y are also plotted laterally resolved at t = 0 s along the white dashed line in x-direction.

6.3 Preliminary experiments for investigating the origin of cytosolic calcium increase upon photothermal heating

First it was investigated which role extracellular Ca^{2+} from the cell medium may have, i.e. if the Ca^{2+} wave after photoexcitation originates from Ca^{2+} entering via ion channels or damaged cell membrane. 150,000 MCF-7 cells were seeded in a petri dish (9.2 cm^2 seeding area) in 2 mL of cell culture medium containing 10% FBS and were incubated overnight. On the next day, hollow capsules embedded with star-shaped Au NPs were added at a density of 2 capsules/cell, and cells were incubated overnight. Cells were stained with Fluo-4, and incubated in DPBS (calcium-free PBS, #SH30028.02, Hyclone), which does not contain Ca^{2+} and Mg^{2+}. Cells were excited at 830 nm with an irradiation area of ca. 12.56 μm^2 (20× objective, LSM880) at P_{laser} = 34.2 mW (at the illumination spot) for Δt_{laser} = 0.039 s. Images were taken every 2 s for 60 times using a 20× objective in the LSM880 set-up. 3 examples are shown in **Figure SI.35**. Data show that the Fluo-4 signal after photoexcitation did not change in comparison to Ca^{2+} containing medium (cf. **Figure SI.29** and **Figure SI.31**). Thus, influx from extracellular Ca^{2+} is not the origin of the photoexcited Ca^{2+} wave.

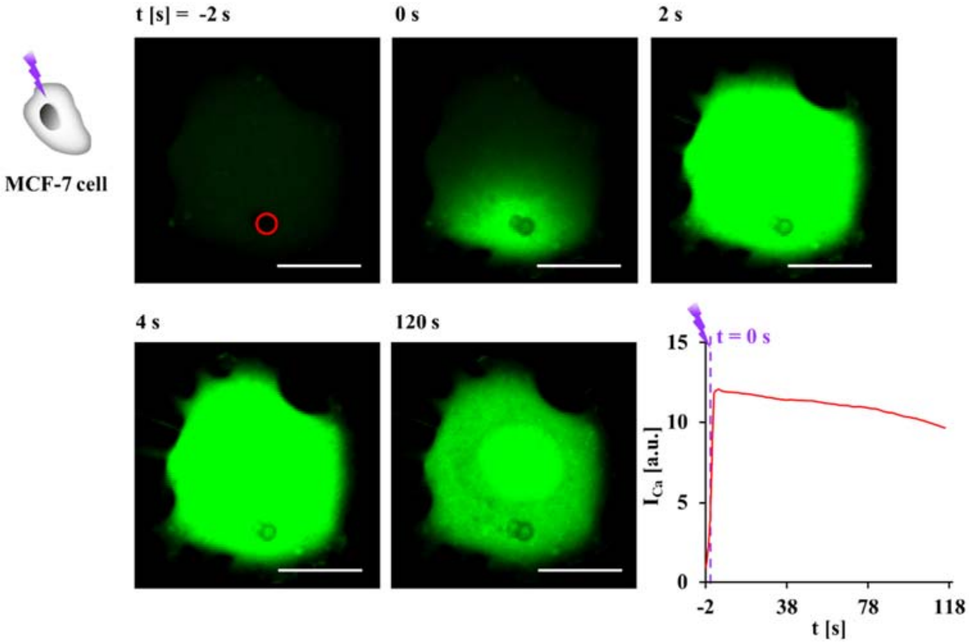

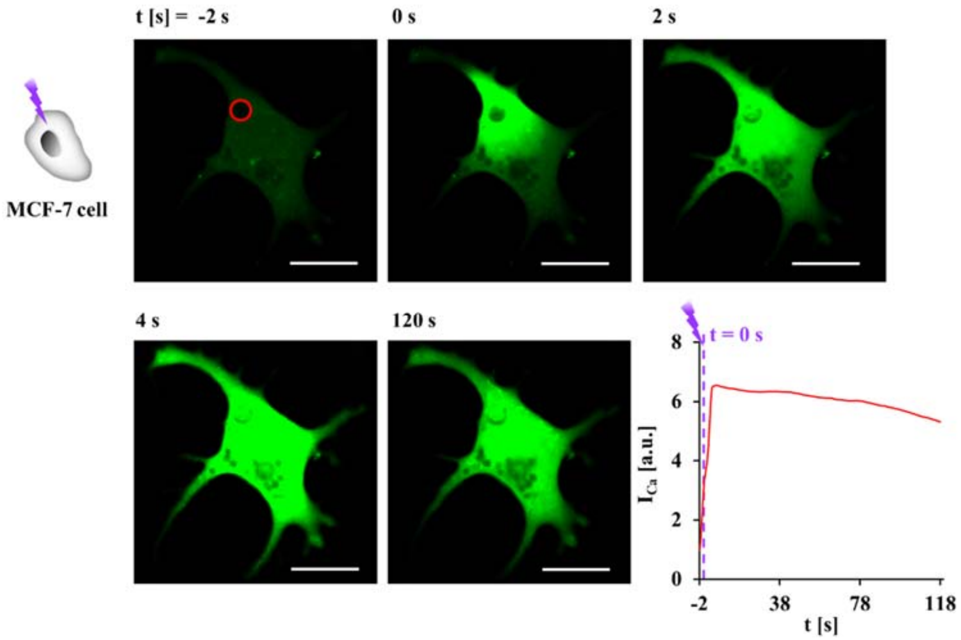

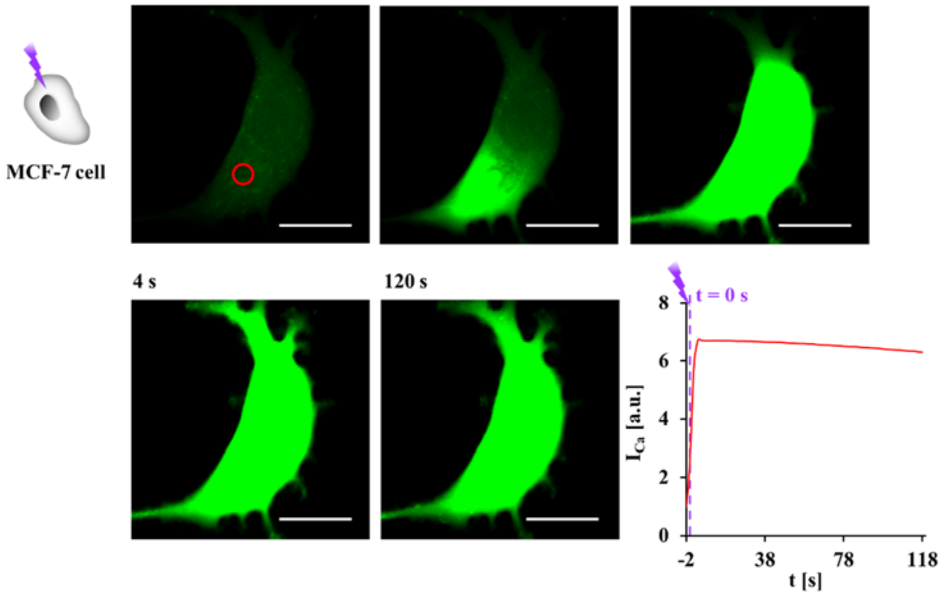

Figure SI.35. At time t = 0 s one capsule with embedded star-shaped Au NPs, as endocytosed by a MCF-7 cell and as indicated by the red circle was exited with an 830 nm laser spot of A_{laser} = 12.56 µm^2 spot size (20× objective, LSM880) at P_{laser} = 28.5 mW (at the illumination spot) for Δt_{laser} = 0.039 s. Cells were incubated in DPBS without Ca^{2+}. Three examples are demonstrated here. Images were taken every 2 s. The scale bars represent 20 µm. The integrated fluorescence intensity of the calcium indicator Fluo-4 I_{Ca} over the cross section of the whole cell area was normalized to that before irradiation (t = -2 s). I_{Ca} relates to the calcium concentration is plotted versus time t.

Next, the possibility of Ca^{2+} induced Ca^{2+} release from the endoplasmic reticulum (ER) was investigated. Here, first thapsigargin was added, which cause Ca^{2+} release from the ER [15]. 150,000 MCF-7 cells were seeded in a petri dish (9.2 cm^2 seeding area) in 2 mL of cell culture medium containing 10% FBS and were incubated overnight. On the next day, hollow capsules with integrated star-shaped Au NPs were added at a density of 2 capsules/cell and cells were incubated overnight to ensure capsule internalization. One vial of 5-(and-6)-carboxy SNARF™-1 acetoxymethyl ester (SNARF-1, #C1272, ThermoFisher) containing 50 µg SNARF-1 was dissolved in 29.3 µL of anhydrous DMSO to prepare a 3 mM stock solution. The stock solution was stored at -20 °C and was protected from light. Before microscopy measurements cells were washed with PBS for 2 times, and incubated with working solution at room temperature for 30 min, which contained 3 µL of 3 mM SNARF-1, 3.9 µL of 1.5 mM Fluo-4 AM and 900 µL of PBS. Afterwards, cells were washed with PBS for 2 times, and incubated at 37 °C in 2 mL of DPBS (i.e. Ca^{2+} free PBS buffer) for 15 min before use. To observe thapsigargin-induced rise in free cytosolic calcium ($[Ca^{2+}]_i$), cells were imaged every 4 s by the 488 nm laser. During the scanning 200 µL of 5.5 µM thapsigargin in DPBS was rapidly added to the cells at a final concentration of 500 nM. The free Ca^{2+} concentration in the cytosol $[Ca^{2+}]_i$ as indicated by the fluorescence of Fluo-4 increased gradually, and was restored to its baseline after 5-10 min, see **Figure SI.36**. This shows the thapsigargin-induced increase of $[Ca^{2+}]_i$.

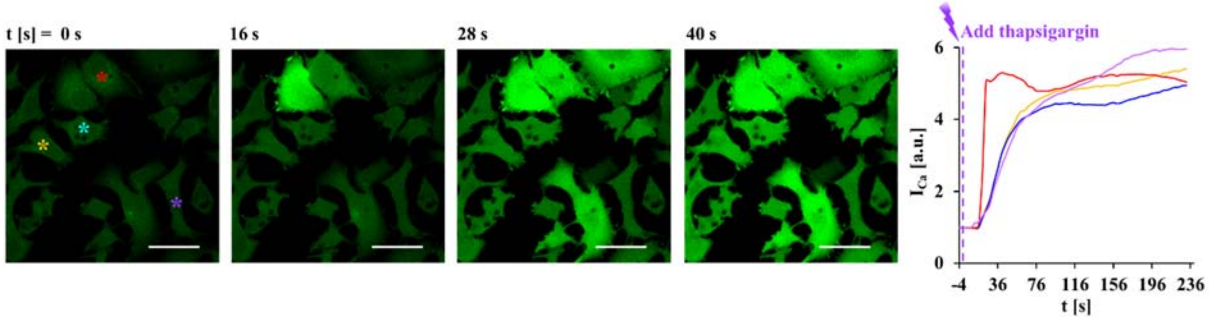

Figure SI.36. Thapsigargin triggered rise of free cytosolic calcium ($[Ca^{2+}]_i$) in MCF-7 cells. 200 µL of 5.5 µM thapsigargin in DPBS was rapidly added to the cultured cells at a final concentration of 500 nM. Images were taken every 4 s. The scale bars represent 50 µm. The integrated fluorescence intensity of the calcium indicator Fluo-4 I_{Ca} over the cross section of the whole cell area was normalized to that before irradiation (t = -4 s), and relates to the calcium concentration. The colors of the curves indicate the cells in which the Fluo-4 intensities were measured, as given by the color of the stars labelling the respective cells.

Afterwards (i.e. once cells were back at normal $[Ca^{2+}]_i$ level) individual cells were excited at 830 nm with an irradiation area of ca. 12.56 μm^2 (20× objective, LSM880) at P_{laser} = 57 mW (at the illumination spot) for Δt_{laser} = 0.004 s (E_{laser} = 0.228 mJ). Images were taken every 0.06 s for 125 times for the Fluo-4 and the 2 SNARF channels using a 20× objective in the LSM880 set-up. 3 examples are shown in **Figure SI.37**. Data indicate that after addition of thapsigargin it is no longer possible to photoexcite a global wave of increased $[Ca^{2+}]_i$, which indicates the role of Ca^{2+} stored in the ER for the propagation of $[Ca^{2+}]_i$. Additional data are shown in **Figure SI.38 - Figure SI.39**.

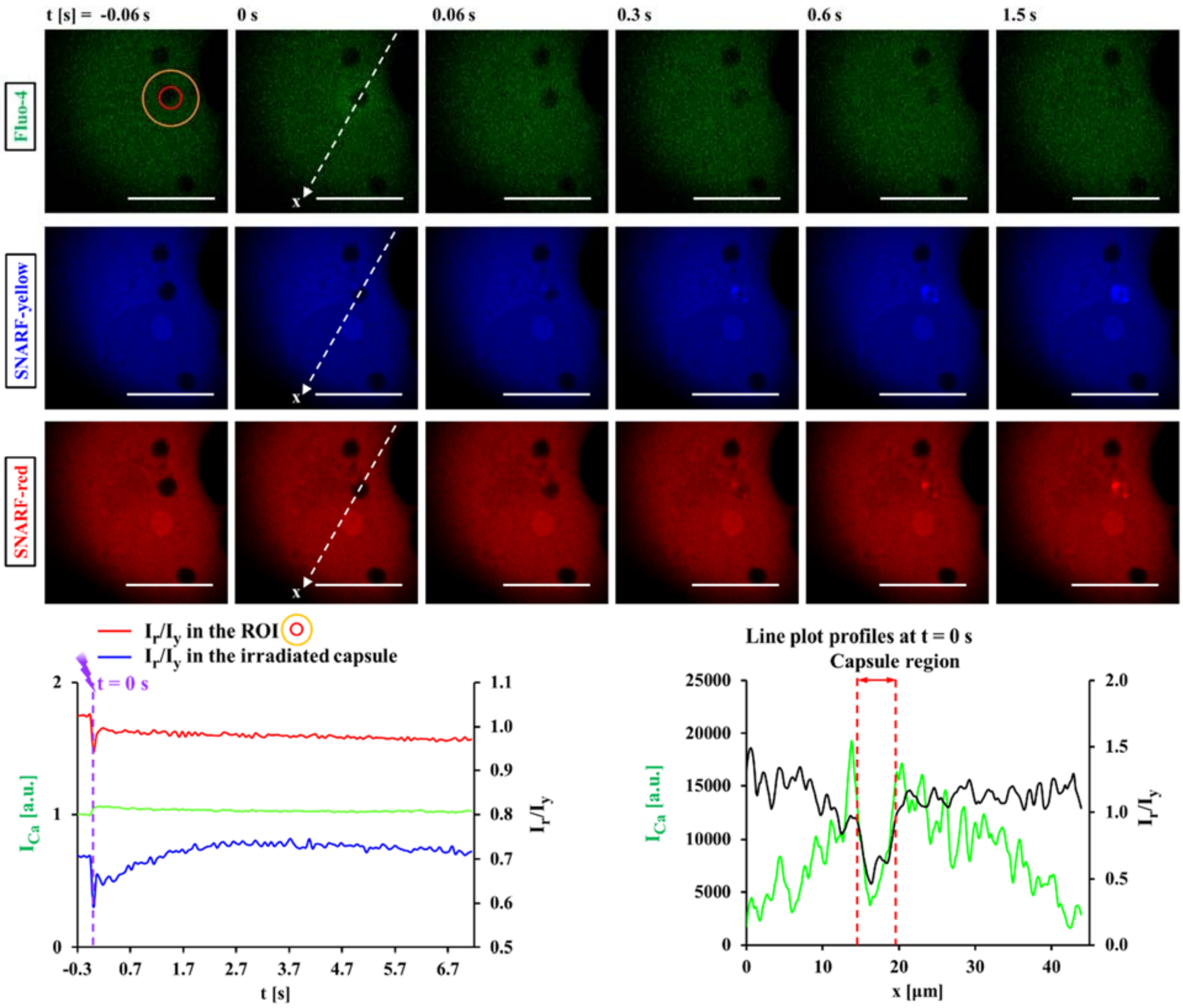

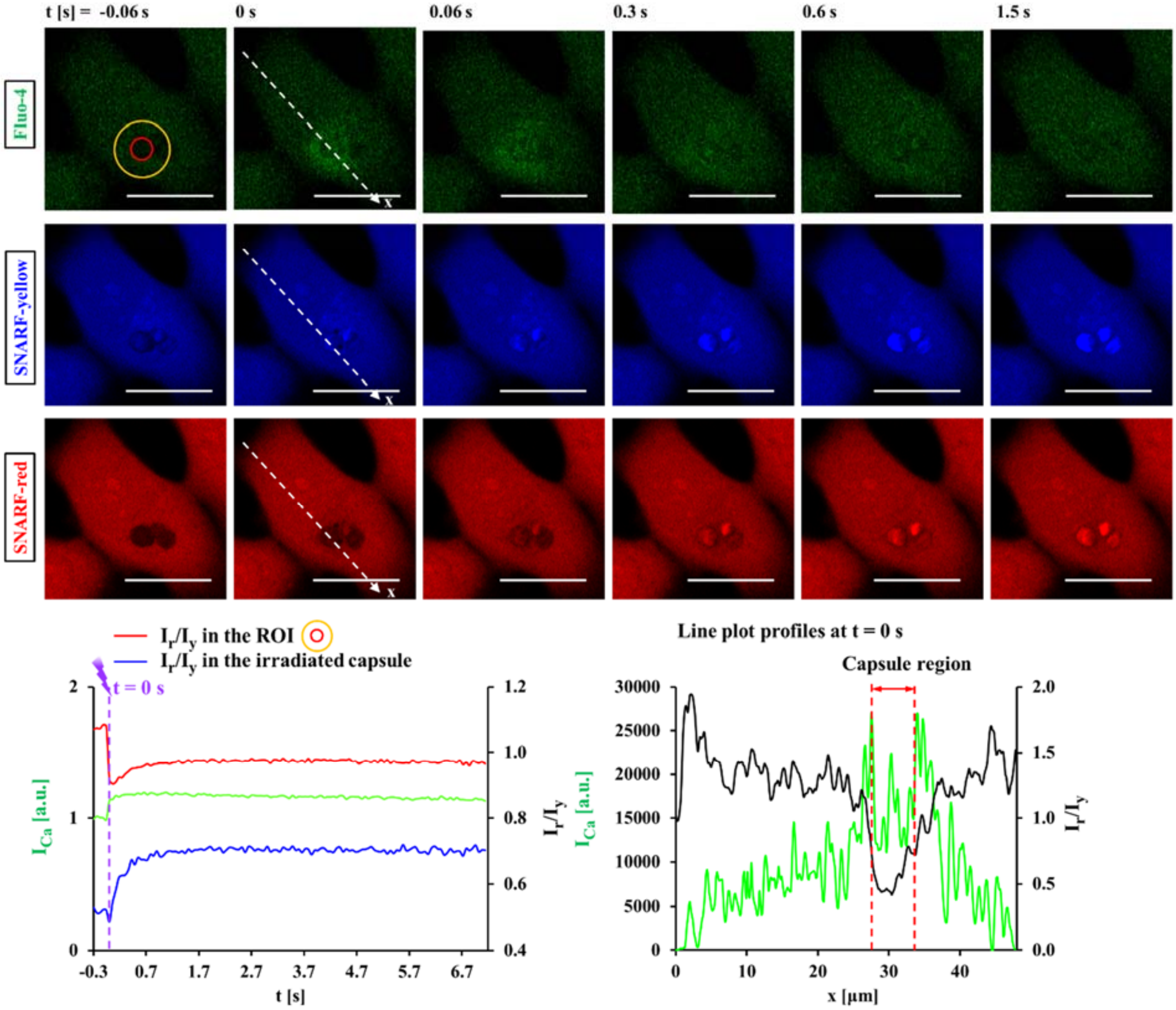

t [s] = -0.06 s
0 s
0.06 s
0.3 s
0.6 s
1.5 s
Fluo-4
SNARF-yellow
SNARF-red
x
I_r/I_y in the ROI
I_r/I_y in the irradiated capsule
t = 0 s
I_Ca [a.u.]
I_r/I_y
t [s]
Line plot profiles at t = 0 s
Capsule region
x [µm]

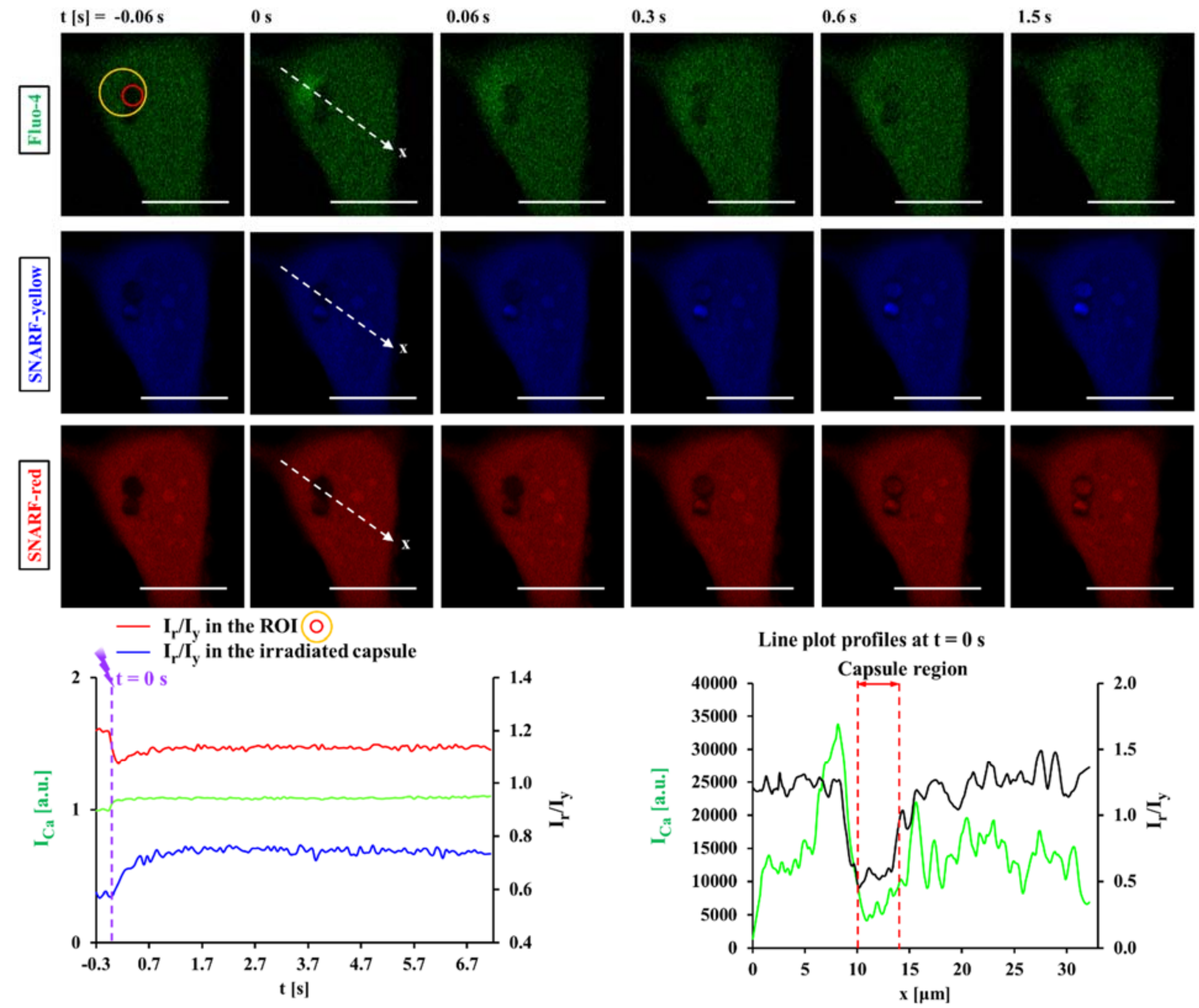

Figure SI.37. MCF-7 cells were first treated with 500 nM thapsigargin and it was waited until $[Ca^{2+}]_i$ restored to its baseline. At time t = 0 s one capsule with embedded star-shaped Au NPs, as endocytosed by a MCF-7 cell, and as indicated by the red circle, was irradiated at 830 nm with an irradiation area of ca. 12.56 μm^2 (20× objective, LSM880) at P_{laser} = 57 mW (at the illumination spot) for Δt_{laser} = 0.004 s. Images were taken every 0.06 s. Three examples are demonstrated here. The scale bars represent 20 µm. The integrated fluorescence intensity of the calcium indicator Fluo-4 I_{Ca} (shown in green) over the cross section of the whole cell area was normalized to that before irradiation t = -0.06 s, and is plotted versus time t. This fluorescence intensity corresponds to $[Ca^{2+}]_i$. The red-to-yellow ratio of the SNARF-1 fluorescence signals I_r/I_y from the location of the irradiated capsule and the surround region of interest (ROI, the area between the yellow and red circle) is also plotted versus time t. I_r/I_y indicates the local pH value. Line plots of the Fluo-4 intensity I_{Ca} and the ratio of the SNARF intensities I_r/I_y at t = 0 s along the while line in x-direction are also plotted.

Additional experiments in the same direction were carried out as follows: 150,000 MCF-7 cells were seeded in a petri dish (9.2 cm^2 seeding area) in 2 mL of cell culture medium containing 10% FBS and were incubated overnight. On the next day, hollow capsules with integrated star-shaped Au NPs were added at a density of 2 capsules/cell and cells were incubated overnight to ensure capsule internalization. Cells were stained with Fluo-4, and incubated in DPBS (#SH30028.02, Hyclone), which does not contain Ca^{2+} and Mg^{2+}. Experiments were now conducted in three steps: First, cells were excited at 830 nm with an irradiation area of ca. 12.56 μm^2 (20× objective, LSM880) at P_{laser} = 34.2 mW (at the illumination spot) for Δt_{laser} = 0.039 s. Images were taken every 2 s for 60 times using a 20× objective in the LSM880 set-up. Second, cells were imaged every 4 s by the 488 nm laser. During the scanning 200 µL thapsigargin in DPBS was rapidly added to the cells to reach a final concentration of 500 nM. The calcium concentration in the cytosol $[Ca^{2+}]_i$ indicated by the fluorescence of Fluo-4 increased gradually, and restored to its baseline after 5-10 min. Third, after this the same cells which had been excited in the first step (i.e. before adding thapsigargin) were excited again at 830 nm with an irradiation area of ca. 12.56 μm^2 (20× objective, LSM880) at P_{laser} = 57 mW (at the illumination spot) for Δt_{laser} = 0.004 s (E_{laser} = 0.228 mJ). Images were taken every 0.06 s for 60 times using a 20× objective in the LSM880 set-up. Examples are shown in **Figure 2** and **Figure SI.38**.

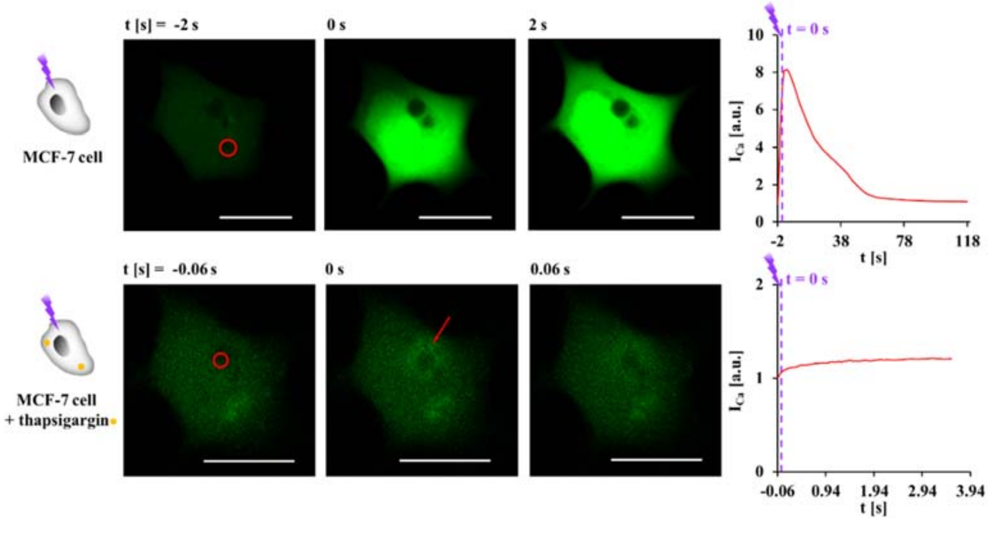

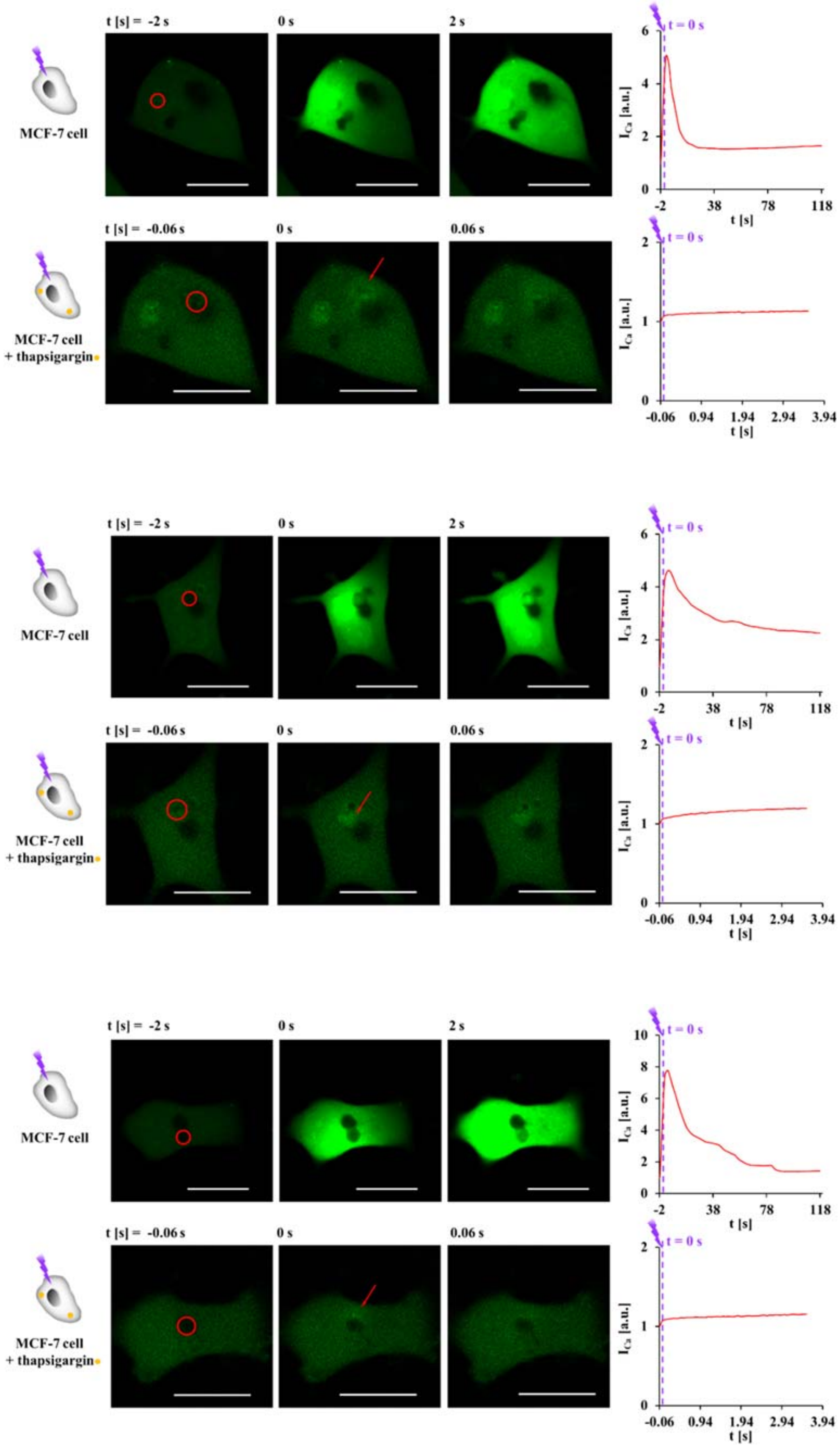

t [s] = -2 s
0 s
2 s
MCF-7 cell
t = 0 s
I_Ca [a.u.]
t [s]
-2
38
78
118
t [s] = -0.06 s
0.06 s
MCF-7 cell
+ thapsigargin
-0.06
0.94
1.94
2.94
3.94

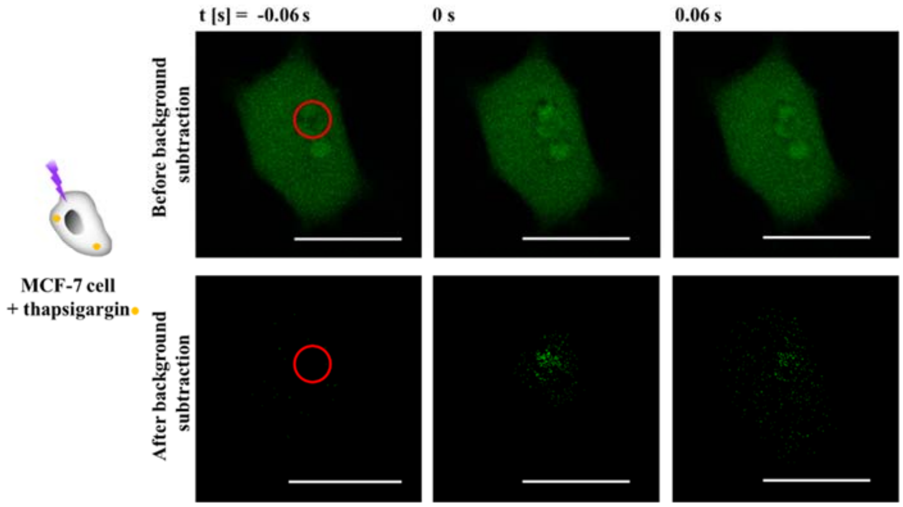

Figure SI.38. Data from the same experiment as shown in **Figure 2,** but recorded with different individual cells. The response to photothermal excitation before addition of thapsigargin (corresponding to **Figure 2a**) and after addition of thapsigargin after relaxation to the base value (corresponding to **Figure 2c**) is shown. Due to the heterogeneity of lysosomes and also in the extent of their transient rupture upon photoexcitation the level of the raise of $[Ca^{2+}]_i$ may vary. Under the presence of thapsigargin increase in $[Ca^{2+}]_i$ in general is small, as there is no subsequent amplification mechanism due to Ca^{2+} release from the ER. In order to better visualize the increase in $[Ca^{2+}]_i$, in the last panel the background fluorescence has been subtracted (more details can be found in §7.2).

6.4 Cytosolic calcium increase in MCF-7 or HeLa cells which were not in direct contact with each other

Widefield microscopy set-up: 15,000 MCF-7 cells or 7,500 HeLa cells were seeded in each well of a 8-well µ-slide in 300 µL of cell culture medium containing 10% FBS and were incubated overnight. Afterwards, hollow capsules with embedded star-shaped Au NPs were added at a density of 2 capsules/cell, and cells were incubated overnight. Cells were stained with Fluo-4, and excited with an 830 nm laser spot of ca. 2.34 μm^2 (63× objective, widefield micrscope) at 0.698 mW (at the illumination spot) for $\Delta t_{laser} = 2$ s. Cells were imaged every 4 s for 30 times using a 63× objective. Data are shown in **Figure SI.39**. Of note, compared to the results shown in **Figure SI.18**, it is rather difficult to determine whether direct excitation of Fluo-4 by the high laser power (i.e. increase in Fluo-4 fluorescence which is not due to increase in the free calcium level in the cytoso) contributed to the increased fluorescence of adjacent cells.

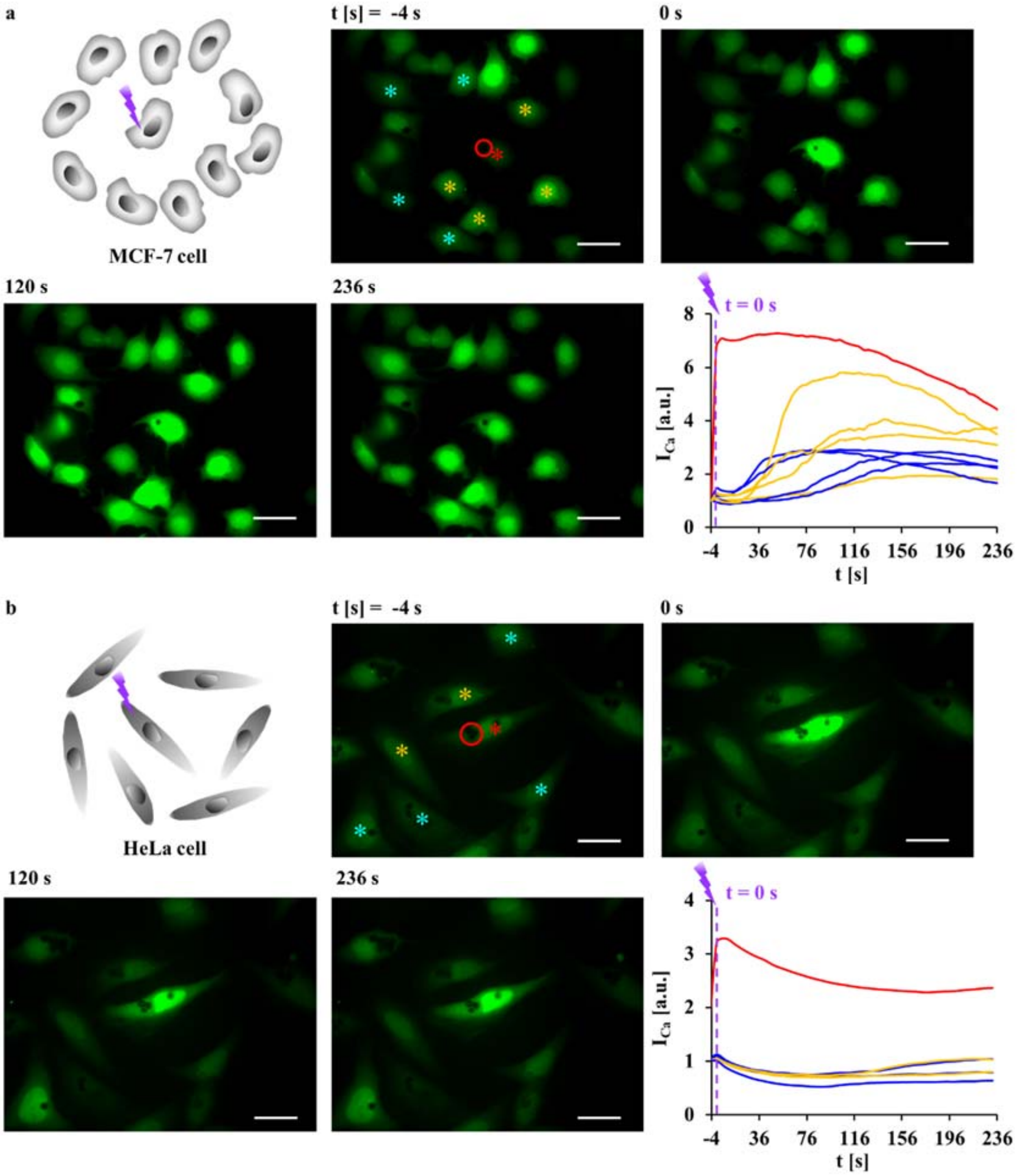

Figure SI.39. a) MCF-7 and b) HeLa cells were seeded at densities in which cells were not in direct contact with each other. At time t = 0 s one endocytosed capsule with embedded star-shaped Au NPs as indicated by the red circle was excited with an 830 nm laser spot of ca. 2.34 μm^2 size (63× objective, widefield microscope) at 0.698 mW (at the illumination spot) for Δt_{laser} = 2 s. Images were taken every 4 s. The scale bars represent 20 µm. The integrated fluorescence intensity of the calcium indicator Fluo-4 I_{Ca} over the cross section of the whole cell area was normalized to that before irradiation (t = -4s), and is plotted versus time t. The colors of the curves refer to the cells in which the Fluo-4 intensities were measured, as given by the color of the stars labelling the respective cells. Red stars indicate the irradiated cell. Yellow or blue stars indicate cells close to or far away from the irradiated cell.

Laser-scanning microscopy set-up (LSM880): 150,000 MCF-7 cells or 75,000 HeLa cells were seeded in a petri dish in 2 mL of cell culture medium containing 10% FBS and were incubated overnight. On the next day, hollow capsules modified with star-shaped Au NPs in their shells were added at a density of 2 capsules/seeded cell, and cells were incubated overnight. Cells were stained with Fluo-4, irradiated at P_{laser} = 34.2 mW (**Figure SI.40**) or P_{laser} = 71.25 mW (**Figure SI.41**) for Δt_{laser} = 0.039 s. Images were taken every 2 s for 120 times using the 20× objective in the LSM880 set-up.

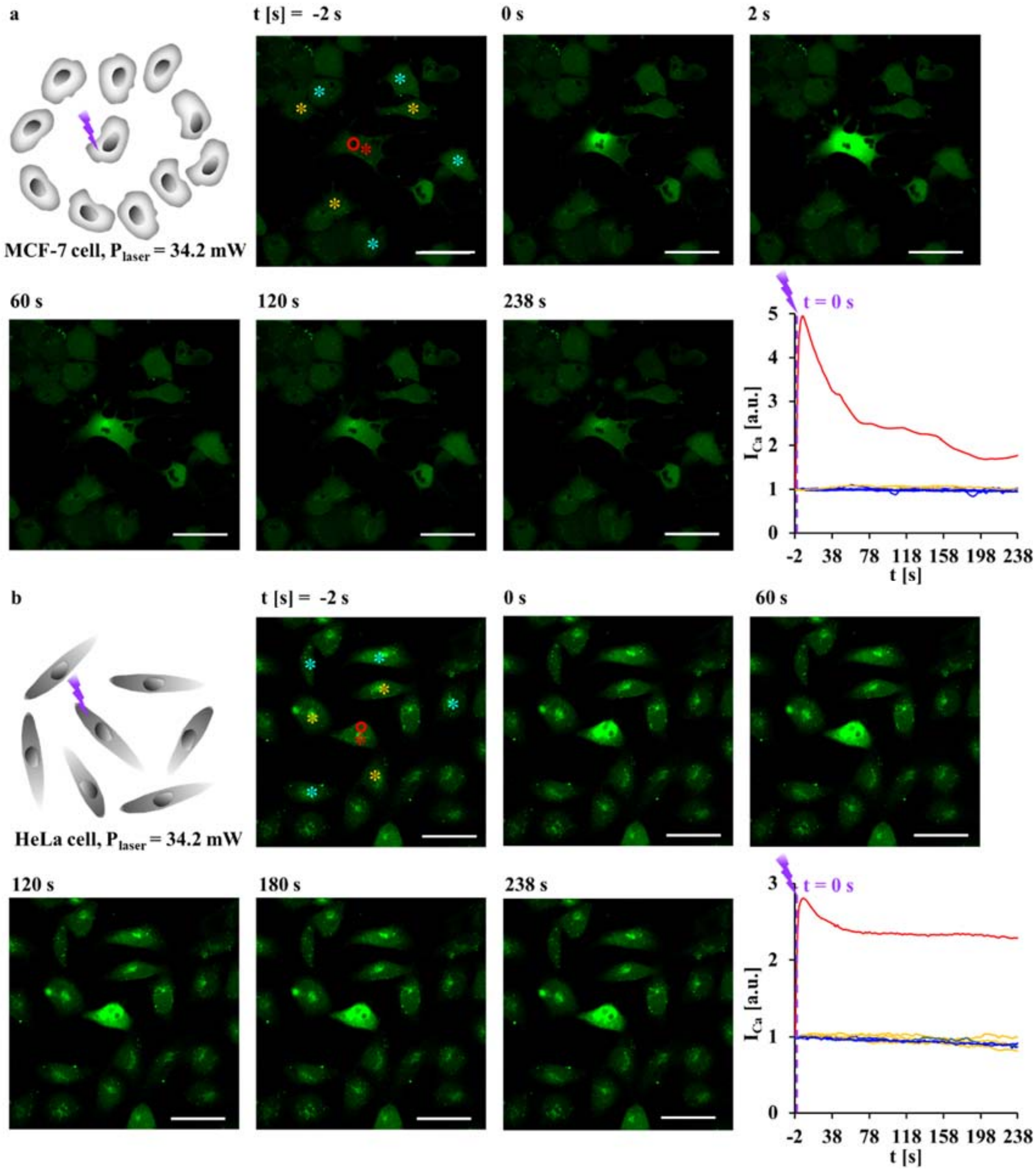

Figure SI.40. a) MCF-7 and b) HeLa cells were seeded at densities in which cells were not in direct contact with each other. At time t = 0 s one endocytosed capsule with embedded star-shaped Au NPs as indicated by the red circle was irradiated at 830 nm with an irradiation area

of A_{laser} = 12.56 μm^2 (20× objective, LSM880) at P_{laser} = 34.2 mW (at the illumination spot) for Δt_{laser} = 0.039 s. Images were taken every 2 s. The scale bars represent 50 μm. The integrated fluorescence intensity of the calcium indicator Fluo-4 I_{Ca} over the cross section of the whole cell area was normalized to that before irradiation (t = -2 s). This intensity relates to the calcium concentration and is plotted versus time t. The colors of the curves indicate the cells in which the Fluo-4 intensities were measured, as given by the color of the stars labelling the respective cells. Red stars indicated the irradiated cells. Yellow or blue stars indicate cells close or far away from the irradiated cell.

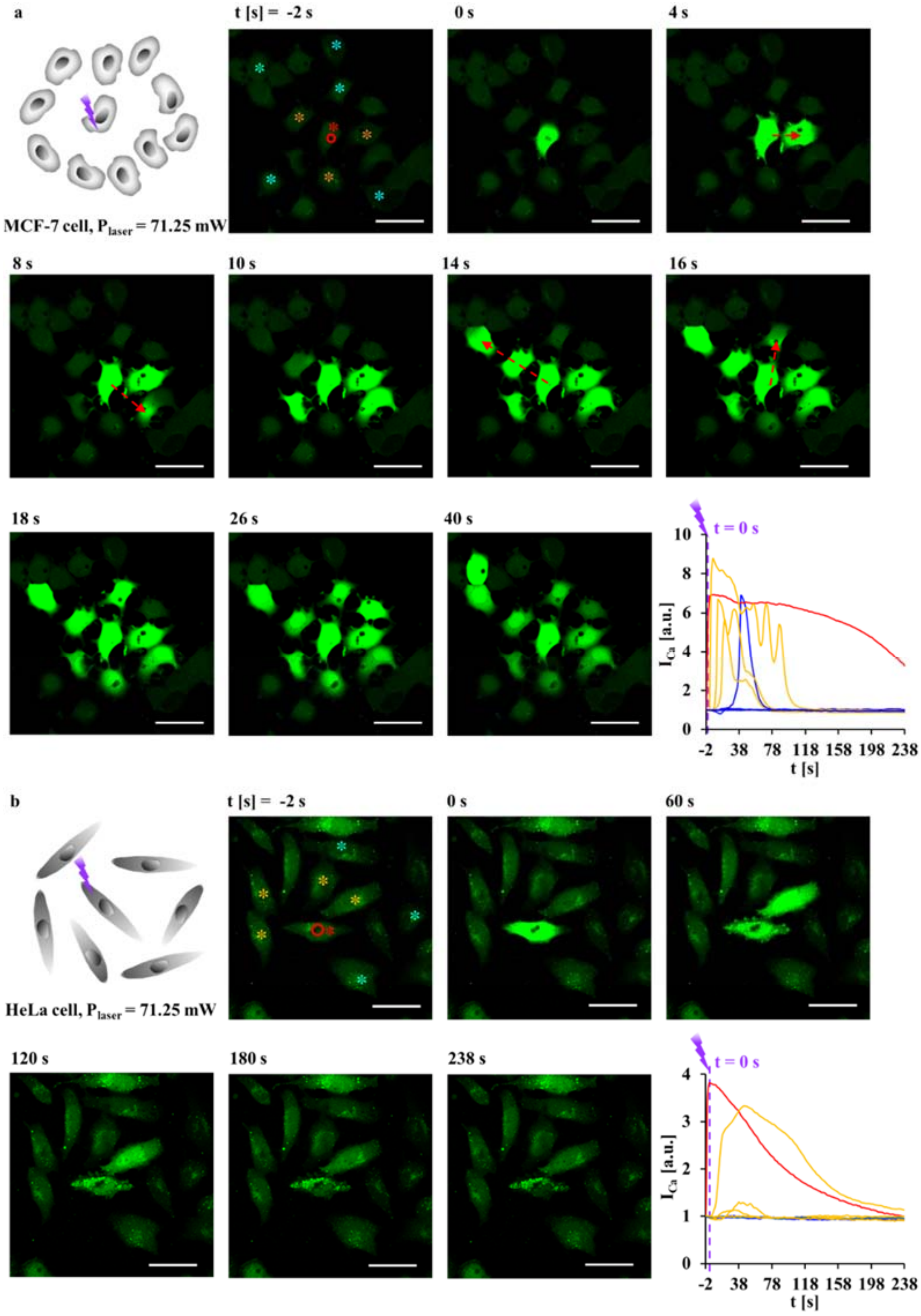

Figure SI.41. a) MCF-7 and b) HeLa cells were seeded at densities in which cells were not in direct contact with each other. At time t = 0 s one endocytosed capsule with embedded star-shaped Au NPs as indicated by the red circle was irradiated at 830 nm within an irradiation area of ca. 12.56 μm^2 (20× objective, LSM880) at P_{laser} = 71.25 mW (at the illumination spot) for Δt_{laser} = 0.039 s. Images were taken every 2 s. The scale bars represent 50 μm. The integrated fluorescence intensity of the calcium indicator Fluo-4 I_{Ca} over the cross section of the whole cell area was normalized to that before irradiation (t = -2 s). The colors of the curves indicate the cells in which the Fluo-4 intensities were measured, as given by the color of the stars

labelling the respective cells. Red stars indicate the irradiated cell. Yellow or blue stars indicate cells close to or far away from the irradiated cell. Red arrows indicate the calcium spread direction from the irradiated cells to adjacent cells.

In order to analyze whether secreted ATP from irradiated cells evokes the cytosolic calcium release in the adjacent cells as for example observed in **Figure SI.41**, the same experiment was conducted in the presence of apyrase by the LSM880 set-up. Apyrase is a calcium-activated plasma enzyme that catalyses hydrolysis of ATP, i.e. would inhibit ATP-mediated signaling between adjacent cells [16]. Briefly, after the Fluo-4 staining, MCF-7 cells were incubated with apyrase (#A7646-200UN, Sigma Aldrich) in the cell culture medium at a final concentration of 60 U/mL for 20 min before imaging. Cells were irradiated at P_{laser} = 71.25 mW for Δt_{laser} = 0.039 s, which is a condition at which paracrine signaling was observed (**Figure 3a**, **Figure SI.41a**). Images were taken every 2 s for 120 times using the 20× objective in the LSM880. After photothermal excitation of free Ca^{2+} in the cytosol of the irradiated cells either no adjacent cells responded, see **Figure SI.42**, or only few cells responded, see **Figure SI.43**.

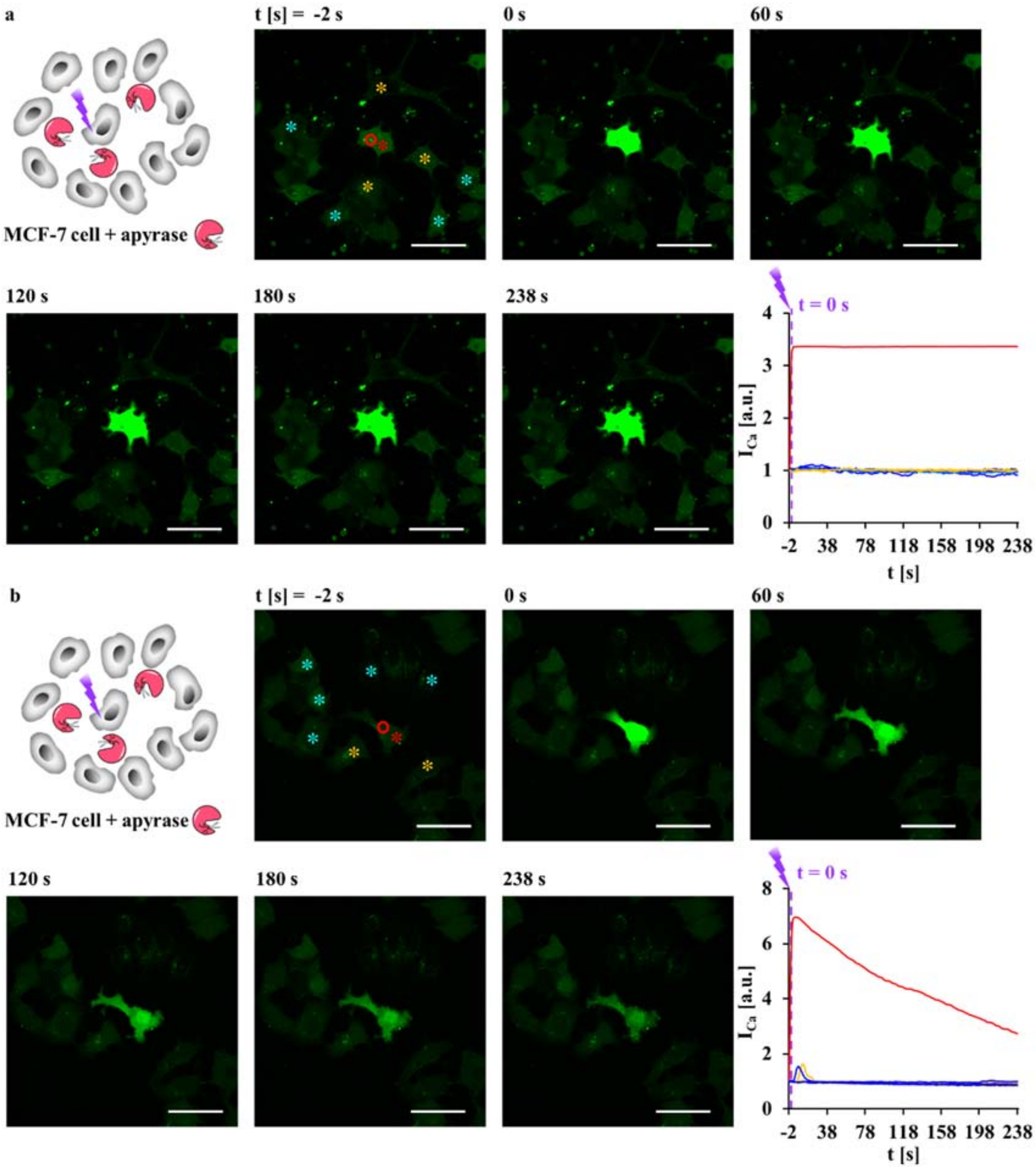

Figure SI.42. MCF-7 cells were seeded at densities in which cells were not in direct contact with each other. Cells were incubated with 60 U/mL apyrase during imaging. At time t = 0 s one endocytosed capsule with embedded star-shaped Au NPs as indicated by the red circle was irradiated at 830 nm with an irradiation area of ca. 12.56 μm^2 (20× objective, LSM880) at P_{laser} = 71.25 mW (at the illumination spot) for Δt_{laser} = 0.039 s. Images were taken every 2 s. Two examples are shown here. The scale bars represent 50 µm. The integrated fluorescence intensity of the calcium indicator Fluo-4 I_{Ca} over the cross section of the whole cell area was normalized to that before irradiation (t = -2 s). The colors of the curves indicate the cells in which the Fluo-4 intensities were measured, as given by the color of the stars labelling the respective cells. Red stars indicate the irradiated cells. Yellow or blue stars indicate cells close to or far away from the irradiated cell.

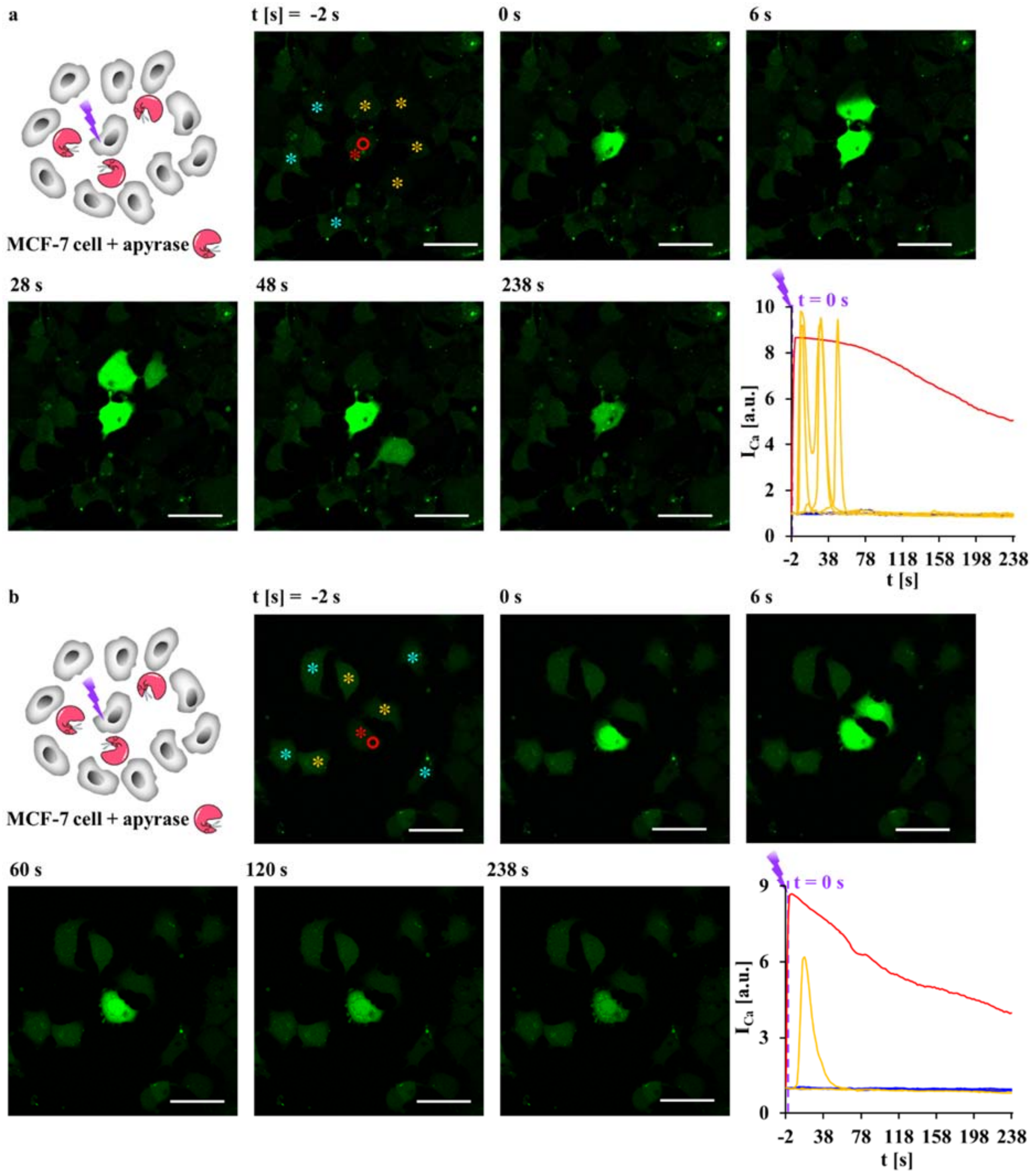

Figure SI.43. MCF-7 cells were seeded at densities in which cells were not in direct contact with each other. Cells were incubated with 60 U/mL apyrase during imaging. At time t = 0 s one endocytosed capsule with embedded star-shaped Au NPs as indicated by the red circle was irradiated at 830 nm with an irradiation area of ca. 12.56 μm^2 (20× objective, LSM880) at P_{laser} = 71.25 mW (at the illumination spot) for Δt_{laser} = 0.039 s. Images were taken every 2 s. Two examples are shown here. The scale bars represent 50 μm. The integrated fluorescence intensity of the calcium indicator Fluo-4 I_{Ca} over the cross section of the whole cell area was normalized to that before irradiation (t = -2 s). The colors of the curves indicate the cells in which the Fluo-

4 intensity were measured, as given by the color of the stars labelling the respective cells. Red star indicated the irradiated cell. Yellow or blue stars indicated cells close to or far away from the irradiated cell.

6.5 Cytosolic calcium increase in MCF-7 or HeLa cells which were physically in contact with adjacent cells

Widefield microscopy set-up: 35,000 MCF-7 cells or 15,000 HeLa cells were seeded in each well of a 8-well µ-slide in 300 µL of cell culture medium containing 10% FBS and were incubated overnight. Afterwards, hollow capsules with star-shaped Au NPs in their walls were added at a density of 2 capsules/cell, and cells were incubated overnight. Cells were stained with Fluo-4, and exited with an 830 nm laser spot of ca. 2.34 μm^2 (63× objective, widefield micrscope) at 0.698 mW (at the illumination spot) for Δt_{laser} = 2 s. Cells were imaged immediately every 4 s using a 63× objective for 60 times. Data are shown in **Figure SI.44.**

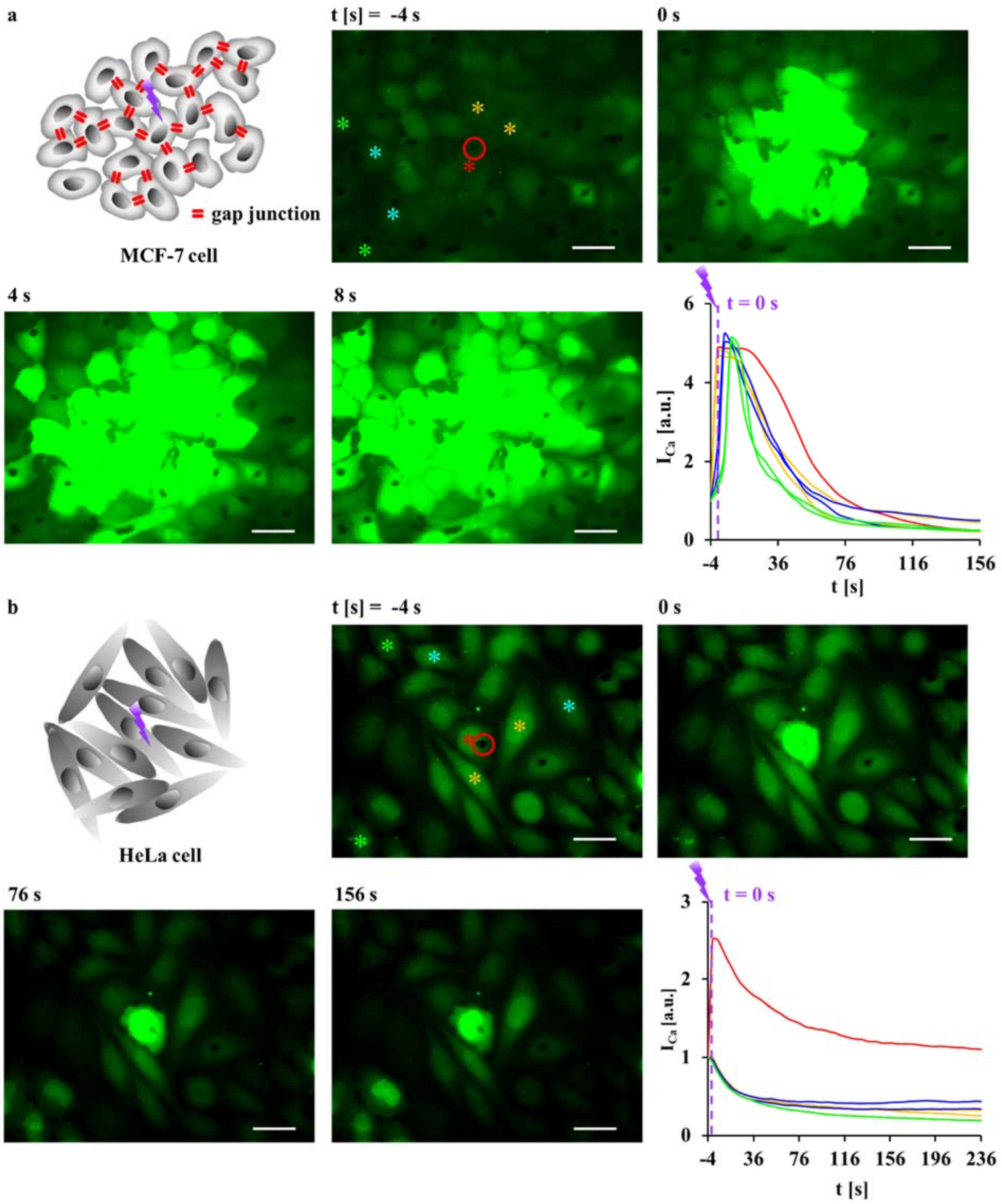

Figure SI.44. a) MCF-7 and b) HeLa cells were seeded at densities in which cells formed an almost confluent layer, enabling physical contact in-between adjacent cells. At time t = 0 s one endocytosed capsule with embedded star-shaped Au NPs as indicated by the red circle was exited with an 830 nm laser spot of A_{laser} = 2.34 μm^2 (63× objective, widefield micrscope) at Plaser = 0.698 mW (at the illumination spot) for Δt_{laser} = 2 s. The scale bars represent 20 μm. The integrated fluorescence intensity of the calcium indicator Fluo-4 I_{Ca} over the cross section of the whole cells labelled with stars was normalized to that before irradiation (t = -4 s). I_{Ca}

relates to the concentration of free calcium in the cytosol of these cells and is plotted versus time t after irradiaten. The colors of the curves indicate the cells in which the Fluo-4 intensities were measured, as given by the color of the stars labelling the respective cells. Red stars indicate the irradiated cells. Yellow, blue or green stars indicate cells with increasing distance from the irradiated cell.

To temporarily block the gap junctions between in-between adjacent MCF-7 cells in physical contact, the MCF-7 cells were incubated with 1 mM octanol (#95446-1ML-F, Sigma Aldrich), a blocker of gap junctions[17], in cell culture medium containing 10% FBS for 1 h at 37 °C. Octanol was diluted in DMSO to prepare a 500 mM stock solution. Afterwards, cells were stained with Fluo-4 in the presence of 1 mM octanol in PBS at room temperature for 30 min. The medium was replaced with fresh cell culture medium containing 10% FBS and 1 mM octanol. Cells were exited with an 830 nm laser spot of ca. 2.34 μm^2 (63× objective, widefield micrscope) at 0.698 mW (at the illumination spot) for $\Delta t_{laser} = 2$ s. Cells were imaged every 4 s for 60 times using a 63× objective. Data are shown in **Figure SI.45**.

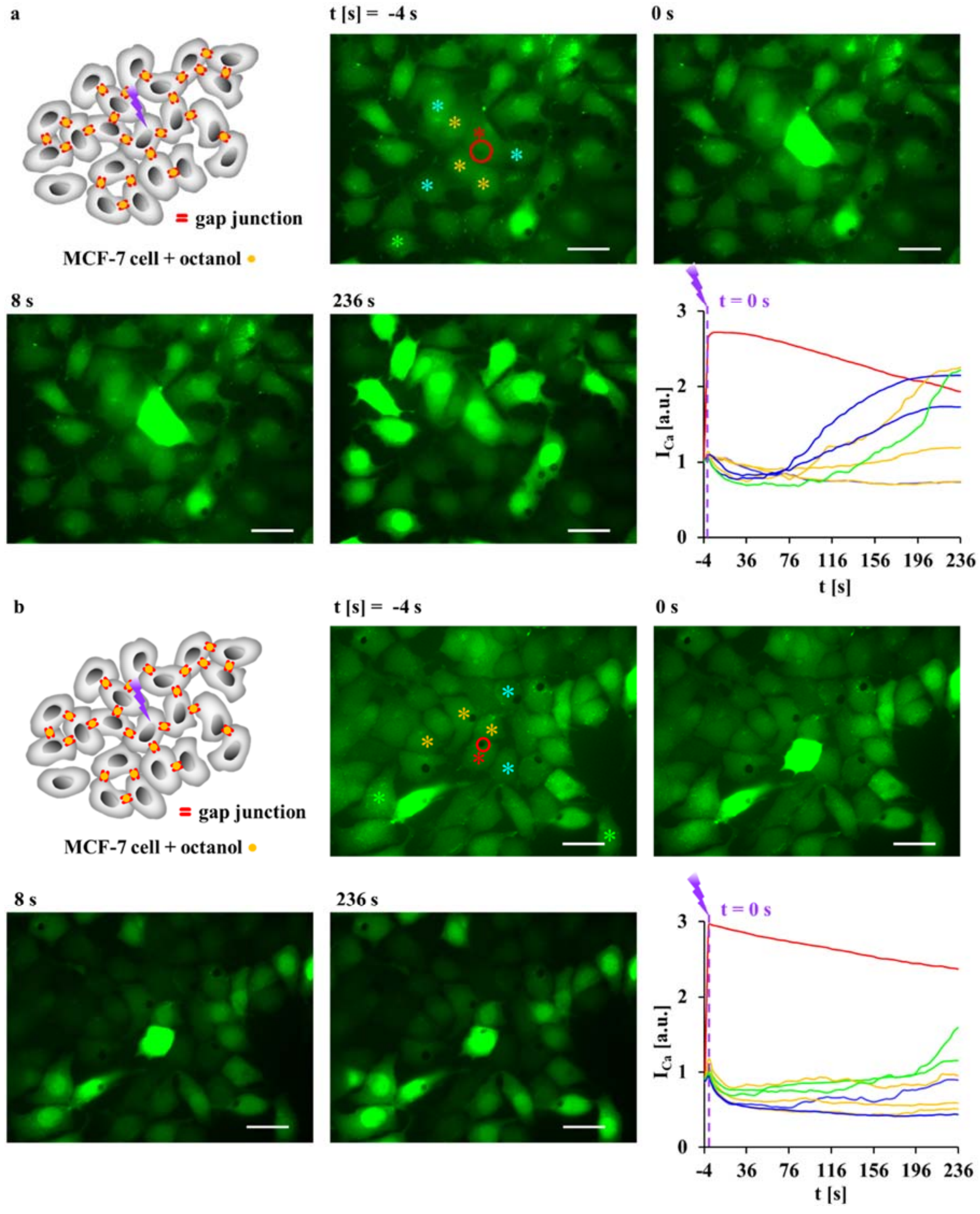

Figure SI.45. MCF-7 cells were seeded at densities in which cells formed an almost confluent layer, enabling physical contact in-between adjacent cells. Cells were incubated with 1 mM octanol. Two examples are demonstrated here. At time t = 0 s one endocytosed capsule with embedded star-shaped Au NPs as indicated by the red circle was exited with an 830 nm laser spot of ca. 2.34 μm^2 (63× objective, widefield microscope) at 0.698 mW (at the illumination spot) for Δt_{laser} = 2 s. The scale bars represent 20 μm. The integrated fluorescence intensity of the calcium indicator Fluo-4 I_{Ca} over the cross section of the whole cells labelled with stars was normalized to that before irradiation (t = -4 s), and is plotted versus time t. The colors of the

curves indicate the cells in which the Fluo-4 intensities were measured, as given by the color of the stars labelling the respective cells. Red stars indicate the irradiated cells. Yellow, blue or green stars indicate cells with increasing distance from the irradiated cell.

Laser-scanning microscopy set-up (LSM880): 350,000 MCF-7 cells or 150,000 HeLa cells were seeded in a petri dish in 2 mL of cell culture medium containing 10% FBS and were incubated overnight. Afterwards, hollow capsules with integrated star-shaped Au NPs were added at a density of 2 capsules/seeded cell, and cells were incubated overnight to ensure capsule internalization. Cells were stained with Fluo-4, and were exited with an 830 nm laser spot of ca. 12.56 μm^2 size (20× objective, LSM880) at 34.2 mW (at the illumination spot) for Δt_{laser} = 0.039 s (**Figure SI.46a,c**). Images were taken every 2 s for 120 times using a 20× objective in the LSM880 set-up. Data are shown in **Figure SI.46.**

To temporarily block the gap junctions between adjacent MCF-7 cells, the MCF-7 cells were incubated with 200 µM carbenoxolone (#C4790-1G, Sigma Aldrich) (**Figure SI.46b**) or 1 mM octanol (**Figure SI.47**) in cell culture medium containing 10% FBS for 1 h at 37°C [17-18]. Carbennoxolone was dissolved in
PBS to obtain 2 mM stock solution. Afterwards, cells were stained with Fluo-4 in the presence of 1 mM octanol or 200 µM carbenoxolone in PBS at room temperature for 30 min. The medium was replaced with fresh cell culture medium containing 10% FBS and 1 mM octanol or 200 µM carbenoxolone. Cells were exited with an 830 nm laser spot of ca. 12.56 μm^2 size (20× objective, LSM880) at 34.2 mW (at the illumination spot) for Δt_{laser} = 0.039 s. Images were taken every 2 s for 120 times using a 20× objective in the LSM880 set-up.

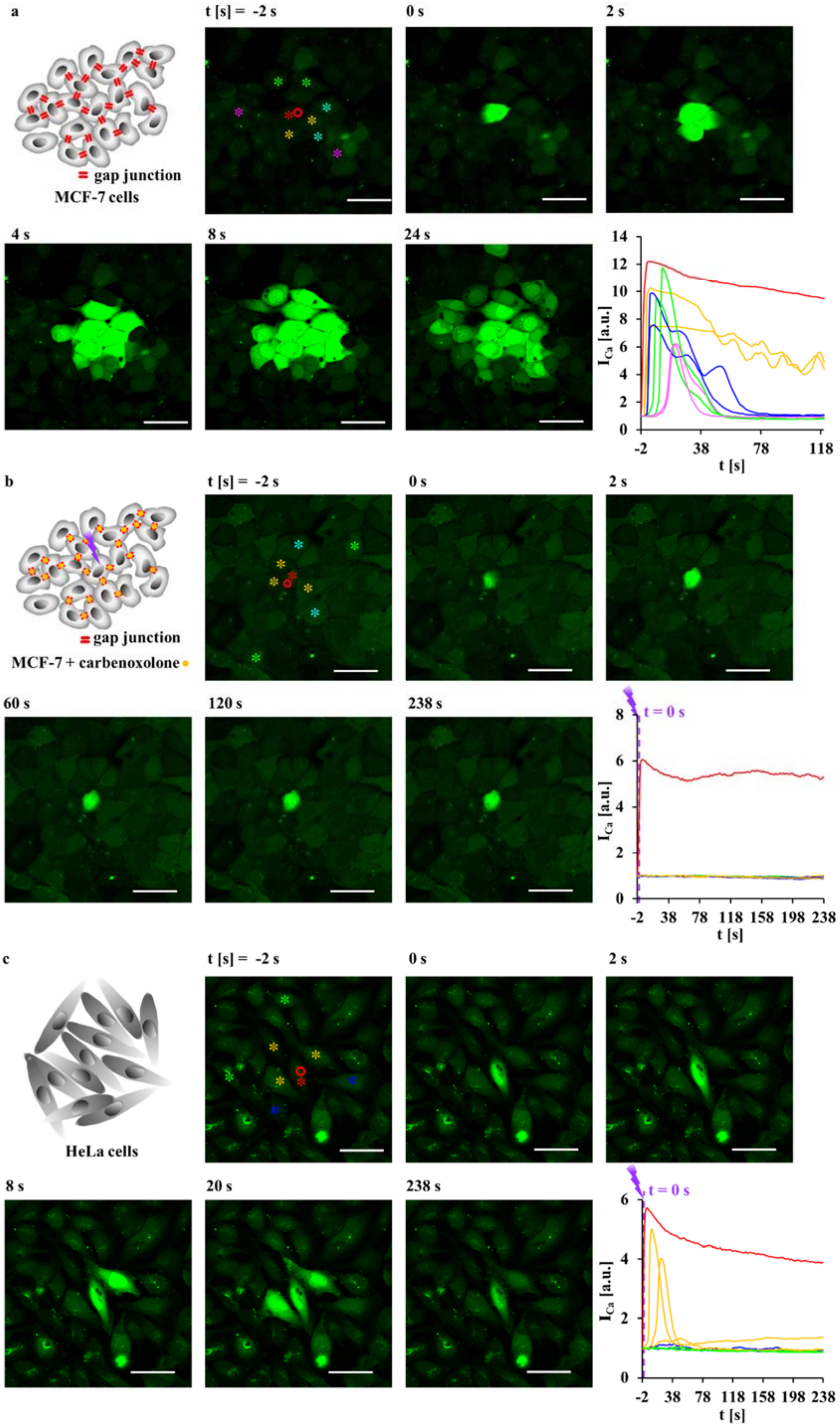
a
gap junction
MCF-7 cells
t [s] = -2 s
0 s
2 s
4 s
8 s
24 s
I_{Ca} [a.u.]
t [s]
b
gap junction
MCF-7 + carbenoxolone
t [s] = -2 s
0 s
2 s
60 s
120 s
238 s
t = 0 s
I_{Ca} [a.u.]
t [s]
c
HeLa cells
t [s] = -2 s
0 s
2 s
8 s
20 s
238 s
t = 0 s
I_{Ca} [a.u.]
t [s]

Figure SI.46. a,b) MCF-7 and c) HeLa cells were seeded at densities in which cells formed an almost confluent layer, enabling physical contact in-between adjacent cells. At time t = 0 s one endocytosed capsule with embedded star-shaped Au NPs as indicated by the red circle was exited with an 830 nm laser spot of ca. 12.56 μm^2 spot size (20× objective, LSM880) at 34.2 mW (at the illumination spot) for Δt_{laser} = 0.039 s. MCF-7 cells were imaged in the a) absence or b) presence of 200 µM carbenoxolone, which blocks gap junctions. The scale bars represent 50 µm. The integrated fluorescence intensity of the calcium indicator Fluo-4 I_{Ca} over the cross section of the whole cells labelled with stars was normalized to that before irradiation (t = -2 s). The colors of the curves indicate the cells in which the Fluo-4 intensity was measured, as given by the color of the stars labelling the respective cells. Red stars indicate the irradiated cells. Yellow, blue, green, and pink stars indicate cells with increasing distance from the irradiated cell.

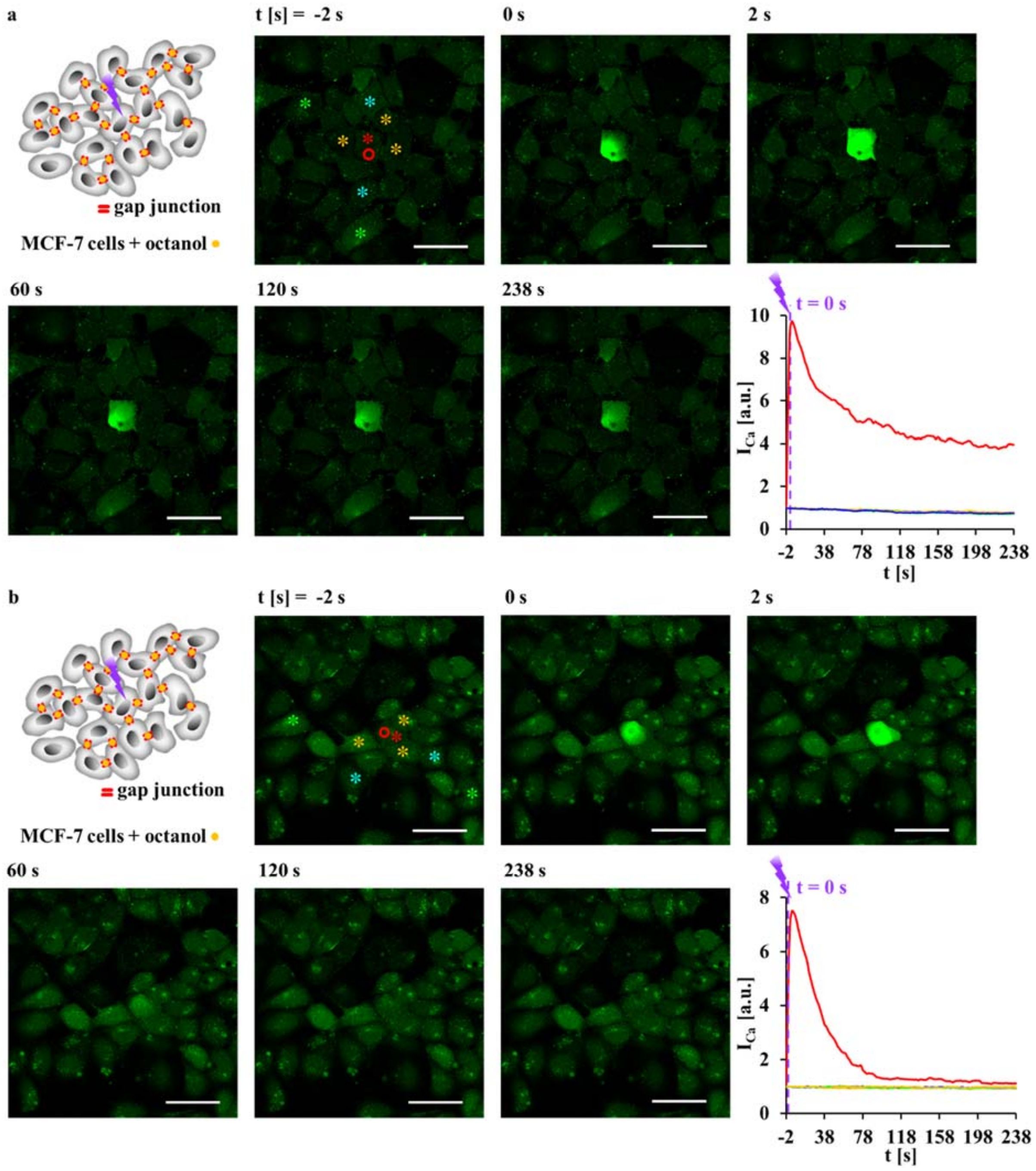

Figure SI.47. MCF-7 cells were seeded at densities in which cells formed an almost confluent layer, enabling physical contact in-between adjacent cells. Cells were incubated with 1 mM of the gap junction blocker octanol. At time t = 0 s one endocytosed capsule with embedded star-shaped Au NPs as indicated by the red circle was exited exited with an 830 nm laser spot of ca. 12.56 μm^2 (20× objective, LSM880) at 34.2 mW (at the illumination spot) for Δt_{laser} = 0.039 s. Two examples are shown here. The scale bars represent 50 µm. The integrated fluorescence intensity of the calcium indicator Fluo-4 I_{Ca} over the cross section of the whole cells labelled with stars was normalized to that before irradiation (t = -2 s). The colors of the curves indicate the cells in which the Fluo-4 intensity was measured, as given by the color of the stars labelling

the respective cells. Red stars indicate irradiated cells. Yellow, blue, and green stars indicate cells with increasing distance from the irradiated cell.

6.6 Cytosolic calcium increase in co-cultured MCF-7/HeLa or MCF-7/NIH 3T3 cells which were physically in contact with adjacent cells

To study if the raise of free calcium in the cytosol of one cell can be signaled to another cells of different type, different cell lines, e.g. dye-labelled HeLa or NIH 3T3 cells were co-cultured with MCF-7 cells. Briefly, 150,000 HeLa or NIH3T3 cells were seeded in each well of 6-well plates in 2.0 mL of cell culture medium containing 10% FBS and were incubated overnight. 1 μL of 1 mM CellTracker Deep Red (#C34565, Thermo Fisher Scientific) in DMSO solution was diluted in 1.5 mL of cell culture medium without serum to prepare the staining solution to stain the HeLa or NIH 3T3 cells. Cells were gently washed with PBS for 2 times, and were incubated with the staining solution at 37 °C for 35 min. Cells were then washed with PBS for 2 times, trypsinized by 0.05% (w/v) trypsin, centrifuged at 300 g for 5 min, and the supernatant was removed. Cells were resuspended in cell culture medium containing 10% FBS. For co-culture, HeLa and MCF-7 cells were mixed at a cell density ratio of 1:3-1:4, while NIH 3T3 and MCF-7 cells were mixed at 1:3 ratio. The cell seeding density was 35,000 cells in each well of 8-well μ-slides (widefield microscope set-up) and 350,000 cells in each petri dish (laser scanning microscope set-up).

Widefield microscopy set-up: 35,000 mixed cells were seeded in per well of an 8-well μ-slide in 300 μL of cell culture medium containing 10% FBS and were incubated overnight. Afterwards, hollow capsules containing star-shaped Au NPs were added at an amount of 2 capsules/cell, and cells were incubated overnight. Cells were stained with Fluo-4. Here only MCF-7 cells with endocytosed capsules were exited with an 830 nm laser spot of ca. 2.34 μm^2 (63× objective, widefield microscope) at 0.698 mW (at the illumination spot) for $\Delta t_{laser} = 2$ s. Cells were imaged every 4 s for 30 times using a 63× objective. The data of cytosolic free calcium increase in co-cultured HeLa and MCF-7 are shown in **Figure SI.48a**, and that in co-cultured NIH 3T3 and MCF-7 are shown in **Figure SI.48b**.

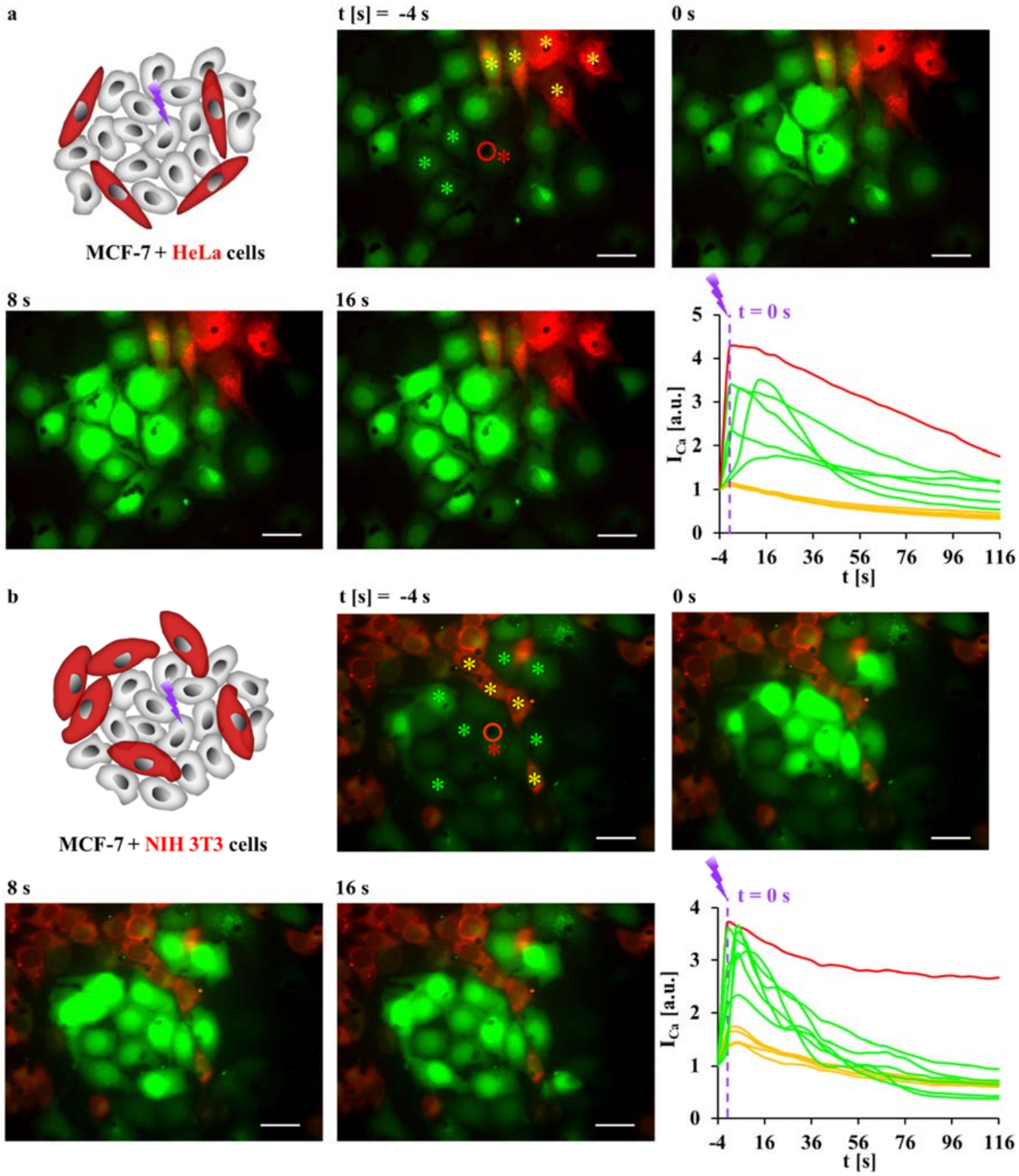

Figure SI.48. MCF-7 cells were seeded in culture with a) HeLa and b) NIH 3T3 cells at densities in which cells formed an almost confluent layer, enabling physical contact in-between adjacent cells. The HeLa and NIH 3T3 cells were labelled with CellTracker Deep Red, and thus they were red fluorescent and can be distinguished from the MCF-7 cells. All cells were loaded with Fluo-4. At time t = 0 s one endocytosed capsule with embedded star-shaped Au NPs in a MCF-7 cell as indicated by the red circle was exited with an 830 nm laser spot of ca. 2.34 µm^2 (63× objective, widefield microscope) at 0.698 mW (at the illumination spot) for Δt_{laser} = 2 s. Images were taken every 4 s. The scale bars represent 20 µm. The integrated fluorescence intensity of the calcium indicator Fluo-4 I_{Ca} over the cross section of the whole cells labelled with stars was normalized to that before irradiation (t = -4 s), and is plotted versus time t. The

colors of the curves indicate the cells in which the Fluo-4 intensity was measured, as given by the color of the stars labelling the respective cells. The red stars indicate the irradiated cells. Green stars indicate adjacent MCF-7 cells. Yellow stars indicate adjacent HeLa or NIH 3T3 cells.

Laser-scanning microscopy set-up (LSM880): 350,000 mixed cells were seeded in each petri dish in 2 mL of cell culture medium containing 10% FBS and incubated overnight. Afterwards, hollow capsules modified with star-shaped Au NPs were added at a concentration of 2 capsules/cell, and cells were incubated overnight. Cells with endocytosed capsules were exited at 830 nm with an irradiation area of A_{laser} = 12.56 μm^2 (20× objective, LSM880) at P_{laser} = 71.25 mW (at the illumination spot) for Δt_{laser} = 0.039 s. Data of free cytosolic calcium increase in co-cultured HeLa and MCF-7 cells are shown in **Figure SI.49** (corresponding to **Figure 5** in the main manuscript), and that in co-cultured NIH 3T3 and MCF-7 cells are shown in **Figure SI.50** (corresponding to **Figure 6** in the manuscript). Irradiation for photothermal heating was performed on capsules endocytosed by either MCF-7, HeLa, or NIH 3T3 cells.

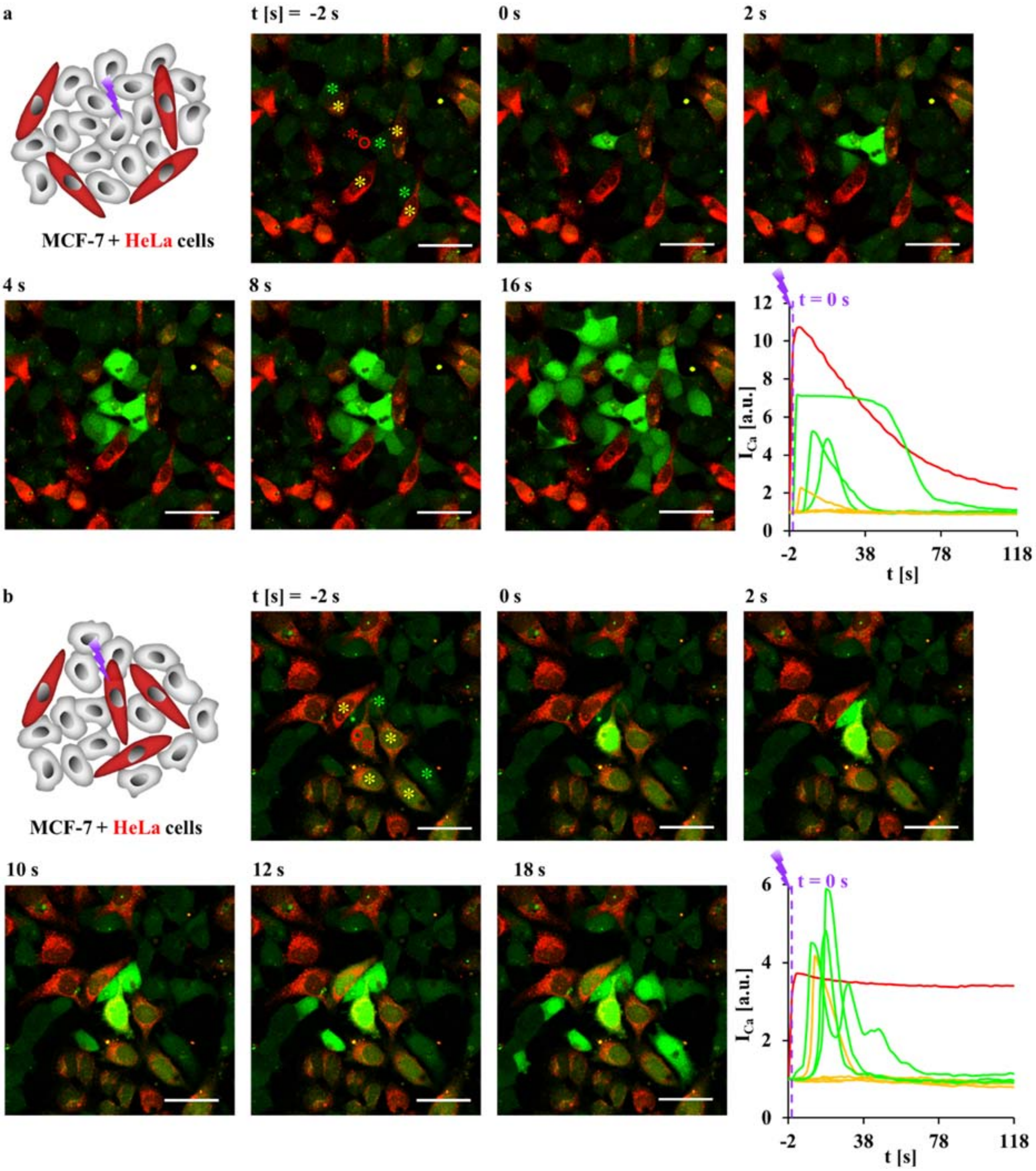

Figure SI.49. MCF-7 cells were seeded in culture with HeLa cells at densities in which cells formed an almost confluent layer, enabling physical contact in-between adjacent cells. The HeLa cells were labelled with CellTracker Deep Red, and thus they were red fluorescent and could be distinguished form MCF-7 cells. All cells were loaded with Fluo-4. At time t = 0 s one endocytosed capsule with embedded star-shaped Au NPs in a a) MCF-7 cell or b) HeLa cell as indicated by the red circles was exited at 830 nm with an irradiation area of ca. 12.56 µm^2 (20× objective, LSM880) at P_{laser} = 71.25 mW (at the illumination spot) for Δt_{laser} = 0.039 s. Images were taken every 2 s. The scale bars represent 50 µm. The integrated fluorescence intensity of the calcium indicator Fluo-4 I_{Ca} over the cross section of the whole cells labelled with stars was normalized to that before irradiation (t = -2 s), and relates to the free cytosolic calcium

concentrations in these cells t. The colors of the curves indicate the cells in which the Fluo-4 intensity was measured, as given by the color of the stars labelling the respective cells. The red stars indicate the irradiated cells. Green stars indicate adjacent MCF-7 cells. Yellow stars indicate adjacent HeLa cells.

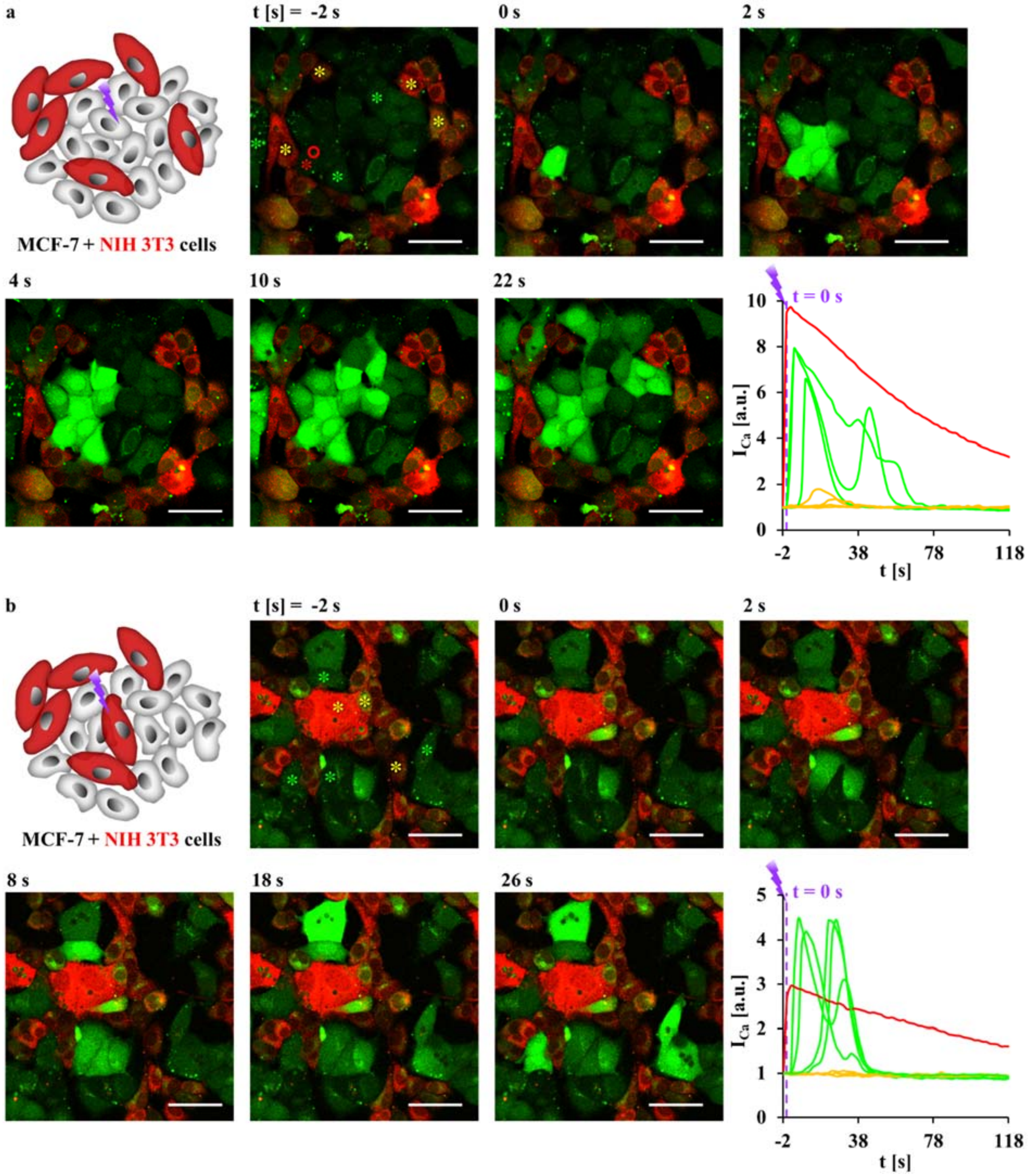

Figure SI.50. MCF-7 cells were seeded in co-culture with NIH 3T3 cells at densities in which cells formed an almost confluent layer, enabling physical contact in-between adjacent cells. The NIH 3T3 cells were labelled with CellTracker Deep Red, and thus they were red fluorescent. At time t = 0 s one endocytosed capsule with embedded star-shaped Au NPs in a a) MCF-7 cell

or b) NIH 3T3 cell as indicated by the red circles was exited at 830 nm with an irradiation area of ca. 12.56 μm^2 (20× objective, LSM880) at P_{laser} = 71.25 mW (at the illumination spot) for Δt_{laser} = 0.039 s. Images were taken every 2 s. The scale bars represent 50 μm. The integrated fluorescence intensity of the calcium indicator Fluo-4 I_{Ca} over the cross section of the whole cells labelled with stars was normalized to that before irradiation (t = -2 s), and is plotted versus time t. The colors of the curves indicate the cells in which the Fluo-4 intensity was measured, as given by the color of the stars labelling the respective cells. The red stars indicate the irradiated cells. Green stars indicate adjacent MCF-7 cells. Yellow stars indicate adjacent NIH 3T3 cells.

7. Measuring cytosolic calcium increase in three-dimensional tumor spheroids

7.1 Spheroid culture
7.2 Cytosolic calcium increase in tumor spheroids after photothermal heating

7.1 Spheroid culture

Two methods have been applied to prepare MCF-7 tumor spheroids in our study.

Widefield microscopy set-up: For studies with the widefield microscope the observed area under the 63× objective was small, and thus small spheroids (ca. 50-100 µm in diameter) were prepared by method I according to a previous report [19]. Briefly, 1% (w/v) agarose (#2267.4, Carl Roth) suspended in PBS was heated until melting in a microwave. After cooling to ca. 60 °C, 1.0 mL of agarose solution was added to each well in a 6-well plate, and the plate was sterilized under UV light for at least 30 min. 20,000 MCF-7 cells were seeded in 1.5 mL of culture medium containing 10% FBS. Cells were cultured for 3-4 days until the spheroidal assemblies had reached the size ca. 50-100 µm in diameter. For capsule internalization, the spheroid suspension was collected in a 2 mL tube, and 30-40 µL of 1.4×10^7/mL empty capsules were added. The mixture was gently shaken in an incubator for 6 h to help capsules associate on the surface of the spheroids. The spheroids were then further cultured overnight to ensure capsule internalization. The morphology of tumor spheroids prepared by method I is shown in **Figure SI.51a**.

Laser-scanning microscopy set-up (LSM880): For the LSM880 set-up observation under the 20× object allowed us to measure a large area, and thus large spheroids (ca. 300-350 µm in diameter) were prepared by method II according to a previous report [20]. 1.05×10^6 cells in 3 mL of cell culture medium were mixed with 0.4×10^6 capsules and 1 mL of 1.2% (w/v) methyl cellulose in water (viscosity: 4000 cp, #M0512-100G, Sigma Aldrich). 20 µL of cell suspension was added onto the lid of a petri dish by multichannel pipettes, e.g. the lid was then covered with several 20 µL droplets. MilliQ water was added to the bottom of the petri dish to avoid the evaporation of cell suspension. The lid was inverted, and cells were incubated for 24 h to assemble into spheroids. Subsequently, the spheroids were transferred to 24-well plates (1.9 cm^2 seeding area per well) with 1% (w/v) solidified agarose and 1 mL cell culture medium.

Spheroids were further cultured for 72 h before use. Compared to the method I, method II can generate larger and more homogeneous tumor spheroids, and the capsules can be incorporated both on the surface and deep inside the spheroids. The morphology of tumor spheroids (without endocytosed capsules) prepared by method II is shown in **Figure SI.51b**.

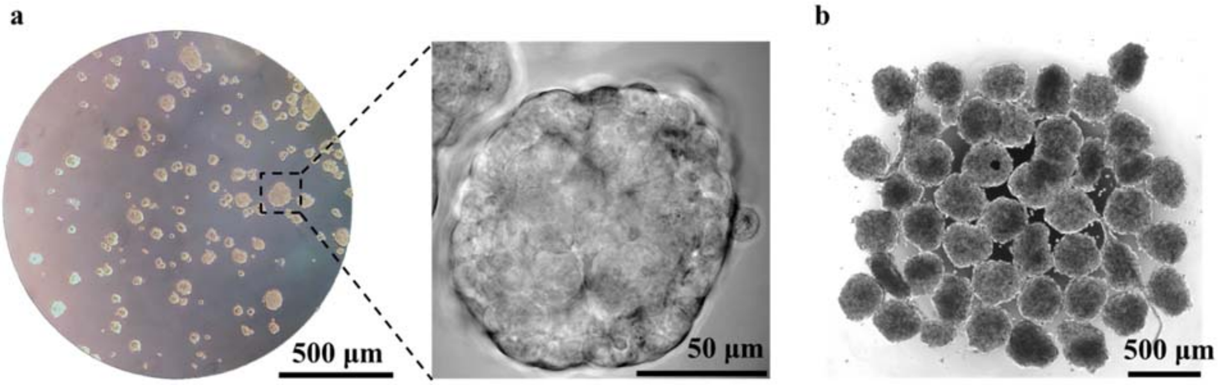

Figure SI.51. Morphology of tumor spheroids without capsules prepared from a) method I and b) method II.

7.2 Cytosolic calcium increase in tumor spheroids after photothermal heating

For staining, the cell culture medium was removed, and the spheroids were washed 3 times by PBS. For staining, spheroids were incubated in 350 µL of PBS containing 3-3.5 µL of 1.5 mM Fluo-4 AM at room temperature for 50-60 min. The medium was replaced by cell culture medium containing 10% FBS, and spheroids were incubated at 37 °C for 15 min before imaging. We noticed that the Fluo-4 cannot stain the cells in the center part of tumor spheroid. Therefore, cells close to surface were studied.

Different from cells in 2-dimensional culture, the Fluo-4 fluorescence in some cells in the spheroids was very bright before irradiation. One example measured by the LSM880 set-up is shown in **Figure SI.52a**. The increase in cytosolic Fluo-4 fluorescence upon photothermal stimulation in spheroids was thus not easy to be distinguished. To overcome this problem, the background fluorescent image (t = -4 s or -8 s) was subtracted from the images after photothermal excitation (t >=0 s) according to the suggestion of a previous publication [21]. The obtained signals were enhanced by multiplying a factor in MATLAB 2019a. The data after background subtraction are shown in **Figure SI.52b**.

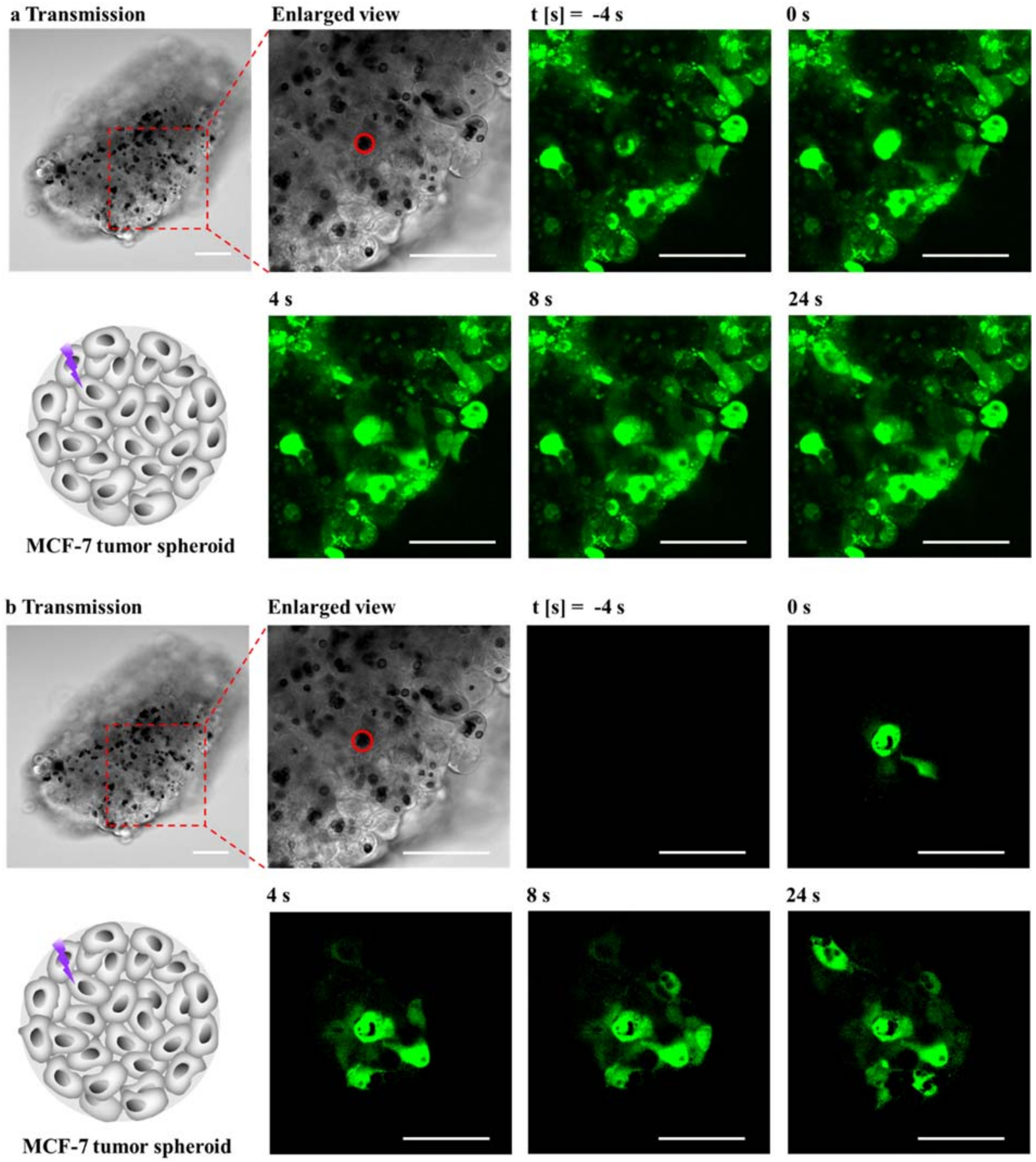

Figure SI.52. MCF-7 cells with capsules with embedded star-shaped Au NPs were seeded in culture to form three-dimensional cell spheroids. At time t = 0 s one capsule as indicated by the red circle was exited at 830 nm with an irradiation area of ca. 43.6 μm^2 (20× objective, LSM880 ste-up) at P_{laser} = 114 mW (at the illumination spot) for Δt_{laser} = 0.175 s. Images were taken every 4 s. The spread of the calcium wave to adjacent cells can be observed by the raise in Fluo-4 fluorescence of the respective cells. The data a) before or b) after background subtraction are compared. The scale bars represent 50 µm.

Widefield microscopy set-up: MCF-7 cells with endocytosed capsules were irradiated at P_{laser} = 2.65 mW (at the illumination spot) for Δt_{laser} = 5 s, and imaged immediately every 8 s using a 63× objective. High laser power was needed to achieve sufficient photothermal heating to excited the release of free calcium, probably due to light adsorption by the spheroid. Data are shown in **Figure SI.53.**

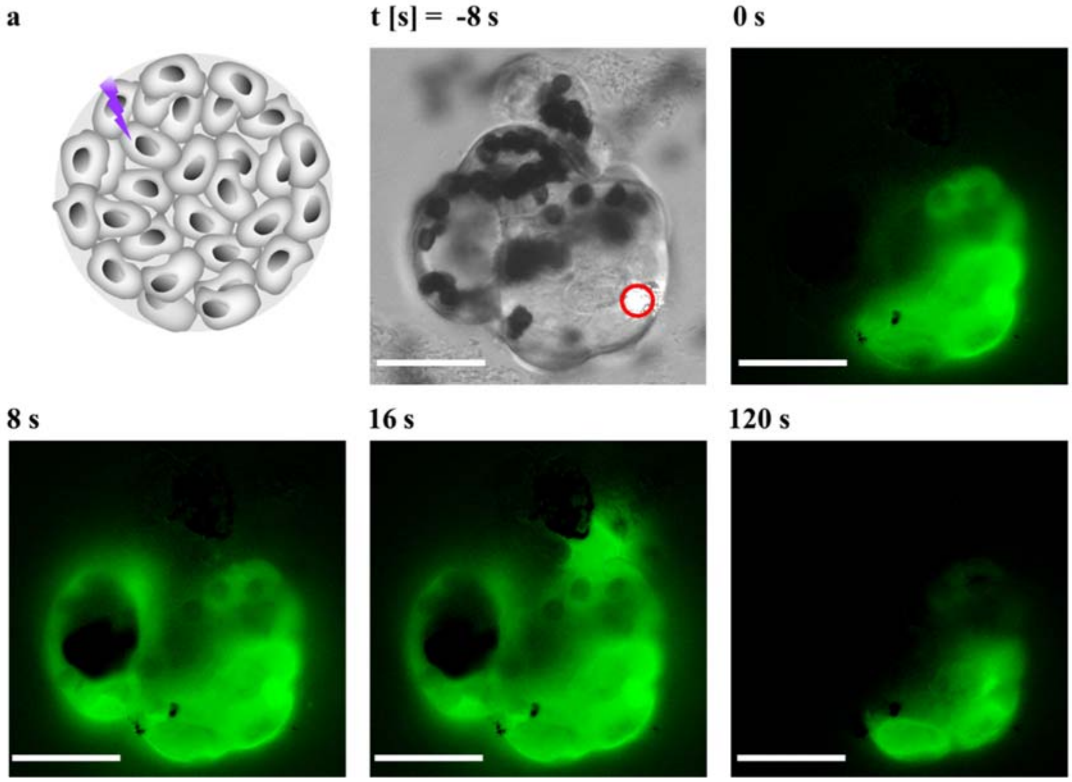

Figure SI.53. MCF-7 cells with capsules with embedded star-shaped Au NPs were seeded in culture to form three-dimensional cell spheroids. At time t = 0 s one capsule as indicated by the red circle was exited at 830 nm with an irradiation area of ca. 2.34 µm^2 (63× objective, widefiled micrscope) at P_{laser} = 2.65 mW (at the illumination spot) for Δt_{laser} = 5 s. Images were taken every 8 s. The spread of the calcium wave to adjacent cells can be observed by the raise in Fluo-4 fluorescence of the respective cells. The scale bars represent 50 µm.

Laser-scanning microscopy set-up (LSM880): MCF-7 cells with endocytosed capsules were exited at 830 nm with irradiation area of ca. 43.6 µm^2 (20× objective, LSM880) at P_{laser} = 114 mW (at the illumination spot) for Δt_{laser} = 0.175 s. Tumor spheroids were imaged immediately every 4 s using a 20× objective. The data are shown in **Figure SI.54.**

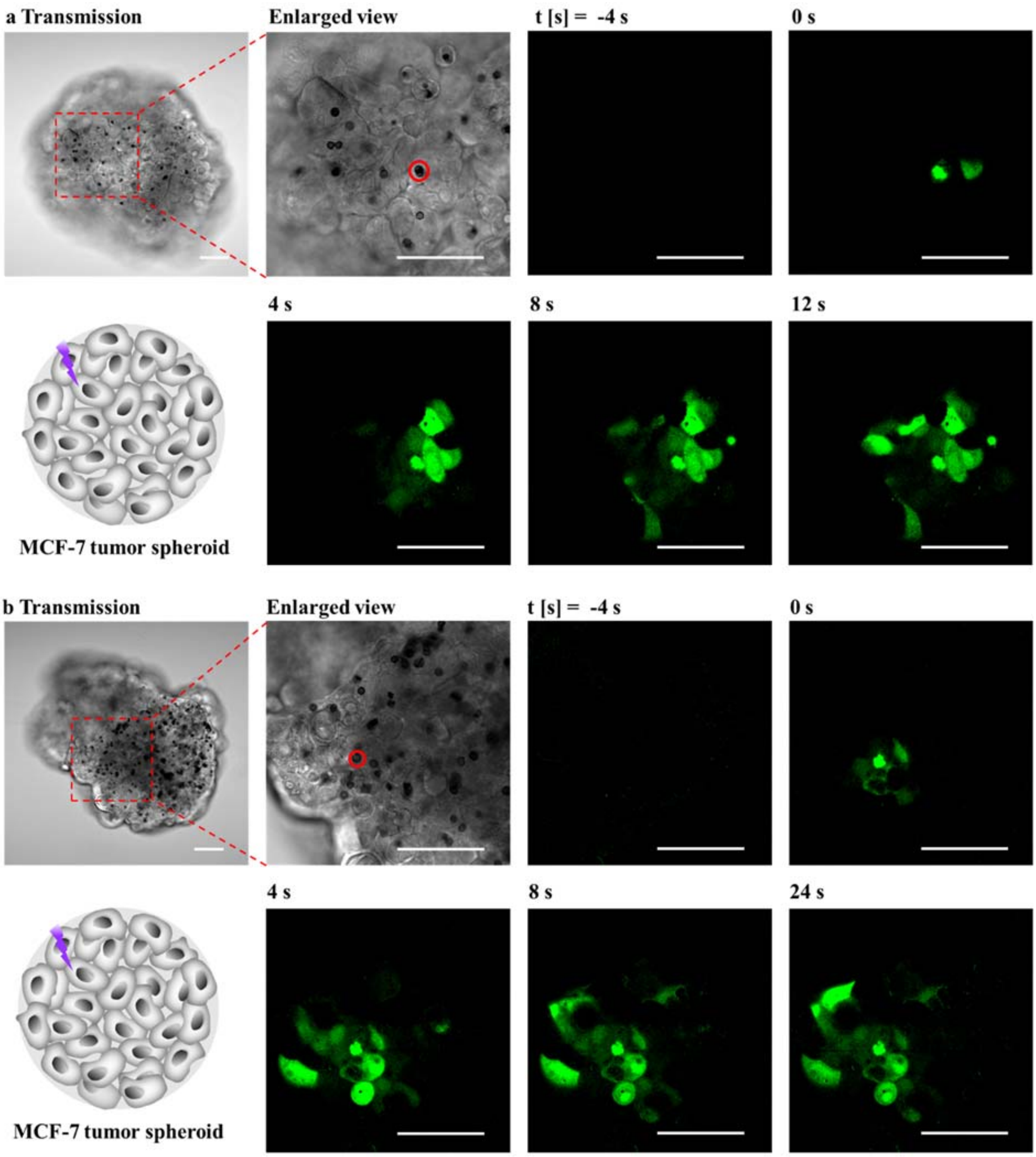

Figure SI.54. MCF-7 cells with capsules with embedded star-shaped Au NPs were seeded in culture to form three-dimensional cell spheroids. At time t = 0 s one capsule as indicated by the red circle was exited at 830 nm with an irradiation area of ca. 43.6 μm^2 (20× objective, LSM880) at P_{laser} = 114 mW (at the illumination spot) for Δt_{laser} = 0.175 s. Two examples are demonstrated here. Images were taken every 4 s. The spread of the calcium wave to adjacent cells can be observed by the raise in Fluo-4 fluorescence of the respective cells. The scale bars represent 50 µm.

With the LSM880 set-up it was also possible to image the Ca^{2+} spread in three dimensions (3d). For this, the formation of spheroids with internalized capsules and their staining was conducted according to the procedure described in §7.1 and §7.2. For imaging, the procedure in §4.1 was modified to ensure both, spatial and temporal resolutions. Scanning at 5 z stacks (i.e., z-axis positions at 16, 8, 0, -8, -16 μm) was conducted. The resolution of the images was decreased from 400 × 400 to 300 × 300 pixels, and the zoom value was increased from 3 to 5, so that the scanning time for each image decreased from 3.85 s to 1.06 s. The time interval between adjacent images was also set as 1.06 s. At t = -0.175 s, only one capsule in the 3rd stack was irradiated at 830 nm with an irradiation area of A_{laser} = 43.6 μm^2 (20× objective, LSM880) at the power P_{laser} = 114 mW (at the illumination spot) for Δt_{laser} = 0.175 s. Scanning started at 1st stack immediately at t = 0 s. For data analysis, the background before irradiation (an averaged matrix from 5 images) was subtracted, and only the increased intensity is shown, as described in §7.2. Because of the much shorter scanning time that was used, more noise was observed in this case after subtracting the background. To compensate for that we used the "ReduceNoise" module in the Cellprofiler V3.1.9 software to remove the noise. To build the three-dimensional model of the increased intensity, the images were processed by the "3D Viewer" plugin in the Fiji software. Results are shown in **Figure SI.55**.

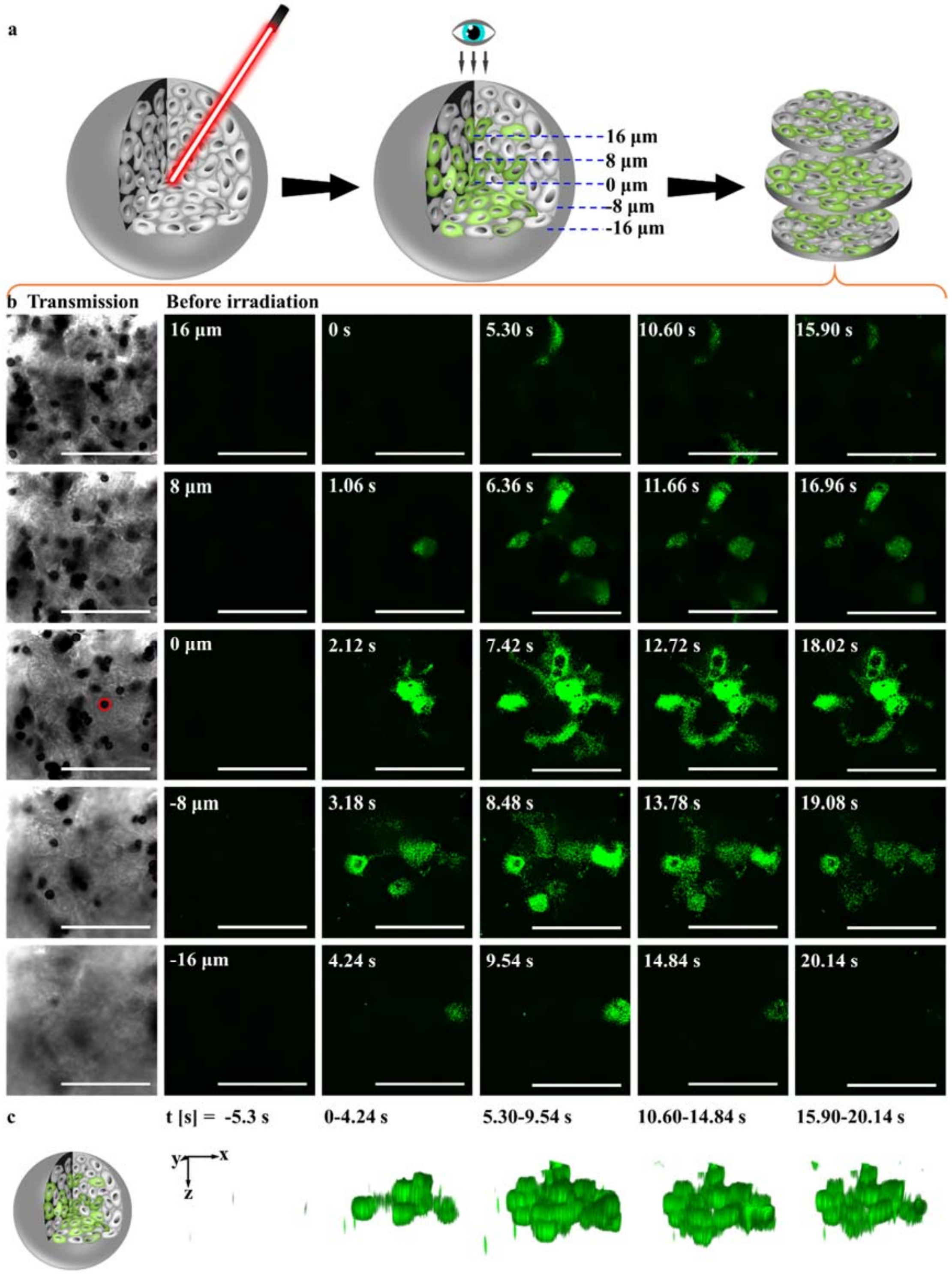

Figure SI.55. MCF-7 cells containing capsules with embedded star-shaped Au NPs were seeded in culture to form three-dimensional cell spheroids. a) Schematic illustration for triggering a calcium wave in a spheroid. b) At time t = 0 s one capsule as indicated by the red circle at a z-axis position of 0 µm was exited at 830 nm with an irradiation area of A_{laser} = 43.6 µm^2 (20× objective, LSM880) at the power P_{laser} = 114 mW (at the illumination spot) for Δt_{laser} = 0.175 s. Images were taken every 1.06 s. The spread of the calcium wave to adjacent cells at

different z-axis positions (i.e., 16, 8, 0, -8, -16 μm) can be observed by the raise in Fluo-4 fluorescence of the respective cells. The scale bars represent 50 μm. c) Three-dimensional modelling of increased Fluo-4 fluorescence at different observation according to the data shown in b).

8. MATLAB code

The imaging processing and data analysis is conducted on MATLAB2019a. Scripts are custom-made according to the different acquirements. One typical script which is designed for demonstrating Fluo-4 increase in tumor spheroid (Section 7.2) after subtracting the background is demonstrated as below. The fluorescence intensity calculation part is also included, even though this function is not used in the case of tumor spheroids.

```
clear;clc;close all;
imtool close all;
clear; workspace;

tic
name_trans = 'trans';
name_green = 'green';
name_ROI = 'ROI';
green_enhance = 2;
trans_enhance = 1;
time = [-20:4:128];

Save_folder = 'Sample 06.tumor spheorid';
folder = uigetdir;
mkdir(Save_folder);

subfolders = genpath(folder);
subfolders = strsplit(subfolders, ';');

files = dir(fullfile(subfolders{1:length(subfolders)}, ['*',name_trans,'*.tif']));

for f = 1:length(subfolders)-1

    disp(['-----> Working in folder ', num2str(f), ' from ', num2str(length(subfolders)-1)])

    files = dir(fullfile(subfolders{f}, ['*',name_trans,'*.tif']));

    for i = 1:5

disp(['--> Analyze the stack ', num2str(i), ' from ', num2str(length(files)), ' stacks'])

im_trans_filename = fullfile(subfolders{f}, files(i).name);
trans_raw = double(imread(im_trans_filename));
green_raw = double(imread(strrep(im_trans_filename, name_trans, name_green)));


im_ROI_raw = fullfile(subfolders{f}, 'ROI.tif');
ROI_raw = imread(im_ROI_raw);

ROI = ROI_raw(:,:,1);
green= green_raw(:,:,2);
trans = trans_raw(:,:,1);

greenlib.data{i} = green*green_enhance/65535;
trans = trans*trans_enhance/65535;

    end
```

```
end

Background =
(greenlib.data{1,1}+greenlib.data{1,2}+greenlib.data{1,3}+greenlib.data{1,4}+greenlib.data{1,5})/5+0.015;


for f = 1:length(subfolders)-1

    disp(['-----> Working in folder ', num2str(f), ' from ', num2str(length(subfolders)-1)])

    files = dir(fullfile(subfolders{f}, ['*',name_trans,'*.tif']));

    for i = 1:length(files)

disp(['--> Analyze the stack ', num2str(i), ' from ', num2str(length(files)), ' stacks'])

im_trans_filename = fullfile(subfolders{f}, files(i).name);
trans_raw = double(imread(im_trans_filename));
green_raw = double(imread(strrep(im_trans_filename, name_trans, name_green)));


im_ROI_raw = fullfile(subfolders{f}, 'ROI.tif');
ROI_raw = imread(im_ROI_raw);

ROI = ROI_raw(:,:,1);

green= green_raw(:,:,2);
trans = trans_raw(:,:,1);

green = green*green_enhance/65535;
trans = trans*trans_enhance/65535;

[labeledImage, numberOfBlobs] = bwlabel(ROI);

green_Blob_Measure = regionprops(labeledImage, green,'Area','MeanIntensity','Centroid');

disp(['Warning! ' num2str(length(green_Blob_Measure)) ' samples detected!!!'])


result(i,1) = time(i);

for j= 1:length(green_Blob_Measure)

    result(i,1+j) = green_Blob_Measure(j).Area * green_Blob_Measure(j).MeanIntensity;

end


green = green - Background;
green(green<0) =0;
green_crop = green(:,8:end);
trans_crop = trans(:,8:end);

RGB_green(:,:,2) = green_crop;
```

```
RGB_green(:,:,1) = 0;
RGB_green(:,:,3) = 0;

RGB_trans(:,:,1) = trans_crop;
RGB_trans(:,:,2) = trans_crop;
RGB_trans(:,:,3) = trans_crop;

RGB_trans_Green(:,:,1) = trans_crop;
RGB_trans_Green(:,:,2) = trans_crop + green_crop;
RGB_trans_Green(:,:,3) = trans_crop;

imwrite(RGB_green,fullfile(Save_folder, [files(i).name, '_green.tif']), 'tif');
imwrite(RGB_trans,fullfile(Save_folder, [files(i).name, '_trans.tif']), 'tif');
imwrite(RGB_trans_Green,fullfile(Save_folder, [files(i).name, '_Merge.tif']), 'tif');

    end

toc

disp(['The running time of folder No', num2str(f), ' is ', num2str(round(toc)), ' s.'])
disp(' ')

end

h = figure;
h_ax = axes('Parent',h);
im_blank = zeros(size(green,1),size(green,2));
imshow(RGB_green);

for jj= 1:length(green_Blob_Measure)

No = num2str(jj);

text(green_Blob_Measure(jj).Centroid(1,1),green_Blob_Measure(jj).Centroid(1,2),No, 'Color', 'r', 'FontSize',
24);

end

LabeledImg = getframe(h_ax);
imwrite(LabeledImg.cdata,fullfile(Save_folder, ['No_on_Img.tif']), 'tif');

close all;


for pp = 1:length(green_Blob_Measure)

result(:,length(green_Blob_Measure)+1+pp) =
5*result(:,1+pp)./(result(1,1+pp)+result(2,1+pp)+result(3,1+pp)+result(4,1+pp)+result(5,1+pp));

end
```

9. References

[1] H. Yuan, C. Khoury, H. Hwang, C. Wilson, G. Grant, T. Vo-Dinh, *Nanotechnology* **2012**, *23*.

[2] K. Kantner, J. Rejman, K. V. L. Kraf, M. G. Soliman, M. V. Zyuzin, A. Escudero, P. d. Pino, W. J. Parak, *Chem. Eur. J* **2018**, *24*, 2098-2102.

[3] aN. Brkovic, L. Zhang, J. N. Peters, S. Kleine-Doepke, W. J. Parak, D. Zhu, *SMALL* **2020**, *16*, 2003639; bM. Ochs, S. Carregal Romero, J. Rejman, K. Braeckmans, S. C. De Smedt, W. J. Parak, *Angew. Chem. Int. Ed.* **2013**, *52*, 695-699.

[4] aM. Xu, M. G. Soliman, X. Sun, B. Pelaz, N. Feliu, W. J. Parak, S. Liu, *ACS Nano* **2018**, *12*, 10104-10113; bX. Sun, M. Gamal, P. Nold, A. Said, I. Chakraborty, B. Pelaz, F. Schmied, K. v. Pückler, J. Figiel, Y. Zhao, C. Brendel, M. Hassan, W. J. Parak, N. Feliu, *Applied Materials Today* **2019**, *15*, 267-279.

[5] X. Li, Z. Che, K. Mazhar, T. J. Price, Z. Qin, *Adv. Funct. Mater.* **2017**, *27*, 1605778.

[6] R. Hartmann, M. Weidenbach, M. Neubauer, A. Fery, W. J. Parak, *Angew. Chem. Int. Ed.* **2015**, *54*, 1365-1368.

[7] S. Roy, D. Zhu, W. J. Parak, N. Feliu, *ACS Nano* **2020**, *14*, 8012-8023.

[8] aT. J. Rink, T. Pozzan, *Cell Calcium* **1985**, *6*, 133-144; bE. Murphy, R. Jacob, M. Lieberman, *J. Mol. Cell. Cardiol.* **1985**, *17*, 221-231.

[9] aS. Carregal-Romero, M. Ochs, P. Rivera Gil, C. Ganas, A. M. Pavlov, G. B. Sukhorukov, W. J. Parak, *J. Control. Release* **2012**, *159*, 120-127; bA. Ott, X. Yu, R. Hartmann, J. Rejman, A. Schütz, M. Ochs, W. J. Parak, S. Carregal Romero, *Chem. Mater.* **2015**, *27*, 1929−1942.

[10] J. n. Vincze, N. Geyer, G. Diszházi, L. J. Csernoch, T. Bíró, I. Jóna, B. Dienes, J. Almássy, *Gen. Physiol. Biophys.* **2018**, *37*, 253-261.

[11] aX. Li, J. F. Lovell, J. Yoon, X. Chen, *Nature Reviews Clinical Oncology* **2020**, 1-18; bY. Liu, P. Bhattarai, Z. Dai, X. Chen, *Chem. Soc. Rev.* **2019**, *48*, 2053-2108.

[12] A. Muñoz Javier, P. del Pino, M. F. Bedard, A. G. Skirtach, D. Ho, G. B. Sukhorukov, C. Plank, W. J. Parak, *Langmuir* **2008**, *24*, 12517-12520.

[13] G. J. Block, G. D. DiMattia, D. J. Prockop, *PLoS One* **2010**, *5*, e10237.

[14] aS. Kalies, G. C. Antonopoulos, M. S. Rakoski, D. Heinemann, M. Schomaker, T. Ripken, H. Meyer, *PLoS One* **2015**, *10*, e0124052; bV. Tseeb, M. Suzuki, K. Oyama, K. Iwai, S. i. Ishiwata, *HFSP journal* **2009**, *3*, 117-123.

[15] aO. Thastrup, P. J. Cullen, B. K. Drøbak, M. R. Hanley, A. P. Dawson, *Proc. Natl. Acad. Sci.* **1990**, *87*, 2466-2470; bY. Kaneko, A. Tsukamoto, *Cancer Lett.* **1994**, *79*, 147-155.

[16] P. B. Guthrie, J. Knappenberger, M. Segal, M. V. Bennett, A. C. Charles, S. B. Kater, *J. Neurosci.* **1999**, *19*, 520-528.

[17] M. L. Cotrina, J. H.-C. Lin, A. Alves-Rodrigues, S. Liu, J. Li, H. Azmi-Ghadimi, J. Kang, C. C. Naus, M. Nedergaard, *Proc. Natl. Acad. Sci.* **1998**, *95*, 15735-15740.

[18] aR. Rozental, M. Srinivas, D. C. Spray, in *Connexin Methods and Protocols*, Springer, **2001**, pp. 447-476; bY. Fujii, S. Maekawa, M. Morita, *Sci. Rep.* **2017**, *7*, 1-15.

[19] J. Friedrich, C. Seidel, R. Ebner, L. A. Kunz-Schughart, *Nat. Protoc.* **2009**, *4*, 309.

[20] H. Liu, Y. Wang, M. Wang, J. Xiao, Y. Cheng, *Biomaterials* **2014**, *35*, 5407-5413.

[21] G. V. Orsinger, J. D. Williams, M. Romanowski, *ACS Nano* **2014**, *8*, 6151-6162.
